# Efferocyte‐Derived MCTRs Metabolically Prime Macrophages for Continual Efferocytosis via Rac1‐Mediated Activation of Glycolysis

**DOI:** 10.1002/advs.202304690

**Published:** 2023-12-08

**Authors:** Duco Steven Koenis, Roberta de Matteis, Vinothini Rajeeve, Pedro Cutillas, Jesmond Dalli

**Affiliations:** ^1^ Centre for Biochemical Pharmacology William Harvey Research Institute Barts and The London School of Medicine and Dentistry Queen Mary University of London London EC1M 6BQ UK; ^2^ Centre for Genomics and Computational Biology Barts Cancer Institute Barts and the London School of Medicine and Dentistry Queen Mary University of London London EC1M 6BQ UK; ^3^ Centre for Inflammation and Therapeutic Innovation Queen Mary University of London London EC1M 6BQ UK

**Keywords:** efferocytosis, macrophages, metabolism, specialized pro‐resolving mediators (SPM), tissue repair

## Abstract

Clearance of multiple rounds of apoptotic cells (ACs) through continual efferocytosis is critical in the maintenance of organ function, the resolution of acute inflammation, and tissue repair. To date, little is known about the nature of mechanisms and factors that govern this fundamental process. Herein, the authors reported that breakdown of ACs leads to upregulation of 12‐lipoxygenase in macrophages. This enzyme converts docosahexaenoic acid to maresin conjugates in tissue regeneration (MCTRs). The levels of these autacoids are elevated at sites of high apoptotic burden in vivo and in efferocytosing macrophages in vitro. Abrogation of MCTR production using genetic approaches limits the ability of macrophages to perform continual efferocytosis both in vivo and in vitro, an effect that is rescued by add‐back of MCTRs. Mechanistically, MCTR‐mediated priming of macrophages for continual efferocytosis is dependent on alterations in Rac1 signalling and glycolytic metabolism. Inhibition of Rac1 abolishes the ability of MCTRs to increase glucose uptake and efferocytosis in vitro, whereas inhibition of glycolysis limits the MCTR‐mediated increases in efferocytosis and tissue repair. Together, these findings demonstrate that upregulation of MCTRs by efferocytosing macrophages plays a central role in the regulation of continual efferocytosis via the autocrine and paracrine modulation of metabolic pathways.

## Graphical abstract

Apoptotic cell uptake by macrophages triggers 12‐lipoxygenase induction and concomitant maresin conjugates in tissue regeneration production via toll‐like receptor 9 and Aryl hydrocarbon receptor signaling. In turn, MCTRs prime macrophages for continual efferocytosis in an autocrine and paracrine manner that is dependent on Rac1 signaling and glycolysis.

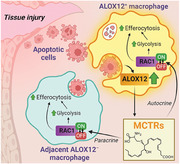

## 1 Introduction

Cell turnover in multicellular organisms is central for maintenance of organ integrity and function.^[1]^ In healthy adult humans, billions of cells undergo apoptosis every day as part of this process.^[^ ^1^ ^]^ Apoptotic cells (ACs) need to be promptly cleared to avert their accumulation and ensuing secondary necrosis, which would release damage‐associated molecular patterns and propagate inflammation.^[1,^ ^2^ ^]^ Disruptions in the ability of macrophages to clear ACs are linked with the onset and perpetuation of multiple inflammatory processes, which in turn contribute to a wide range of pathological conditions.^[^ ^3^, ^4^, ^5^, ^6^ ^]^ Intriguingly, in both physiological and pathological states the number of ACs far exceeds the number of macrophages available to clear them. Therefore, each macrophage needs to clear multiple rounds of ACs, a process termed as continual efferocytosis.^[^ ^7^ ^]^

Uptake of multiple ACs requires a coordinated cellular response to ensure the identification and clearance of the target cell, the efficient breakdown of the metabolic load, and the availability of sufficient energy to maintain this process.^[^ ^7^ ^]^ Recent studies have begun to unravel the cellular and molecular pathways engaged by macrophages to ensure the efficient disposal of multiple cells. These include the non‐canonical engagement of glycolytic pathways,^[^ ^8^ ^]^ mitochondrial fission,^[^ ^9^ ^]^ and the upregulation of polyamine production.^[^ ^7^ ^]^ Furthermore, these studies suggest that factors released during the uptake of the first AC, such as lactic acid, prime macrophages for the uptake of subsequent cells.^[^ ^8^, ^10^ ^]^

Specialized pro‐resolving mediators (SPM) are a class of lipid mediators produced via the metabolic conversion of omega‐3 and omega‐6 polyunsaturated fatty acids (PUFA).^[^ ^11^ ^]^ SPM are potent and stereo‐selective agonists of inflammatory resolution by reprogramming phagocyte responses to enhance bacterial phagocytosis, dampen pro‐inflammatory mediator production, and limit recruitment of inflammatory leukocyte populations.^[^ ^11^ ^]^ Within the SPM family, the maresins (MaR) and maresin conjugates in tissue regeneration (MCTRs) enhance the ability of macrophages to uptake the first round of ACs,^[^ ^12^, ^13^ ^]^ as well as promote tissue regeneration in planarian flatworms.^[^ ^13^, ^14^ ^]^ However, the mechanisms that regulate SPM production in efferocytosing macrophages, as well as whether SPM play a role in the regulation of continual efferocytosis remain unknown.

Herein, we address this fundamental knowledge gap demonstrating that AC‐derived DNA upregulated 12‐lipoxygenase (ALOX12) expression in efferocytosing macrophages. Human ALOX12 (and mouse Alox12/15) converted docosahexaenoic acid (DHA) to MCTRs. These mediators in turn rapidly primed macrophages by modulating Rac1 signaling and glycolytic metabolism, two pathways crucial for effective efferocytosis.^[^ ^8^, ^10^, ^15^ ^]^ Rac1 inhibition abrogated the ability of MCTRs to enhance glucose uptake and efferocytosis in vitro. Functionally, inhibition of either Rac1 activity or glycolysis and the associated reduction in continual efferocytosis was linked with the abrogation of the tissue‐regenerative activities of MCTRs. Taken together, our findings suggest MCTRs are endogenous enhancers of continual efferocytosis and tissue repair responses by macrophages.

## 2 Results

### 2.1 AC‐Derived DNA Upregulates ALOX12 Expression in Efferocytosing Macrophages

**Figure 1:**
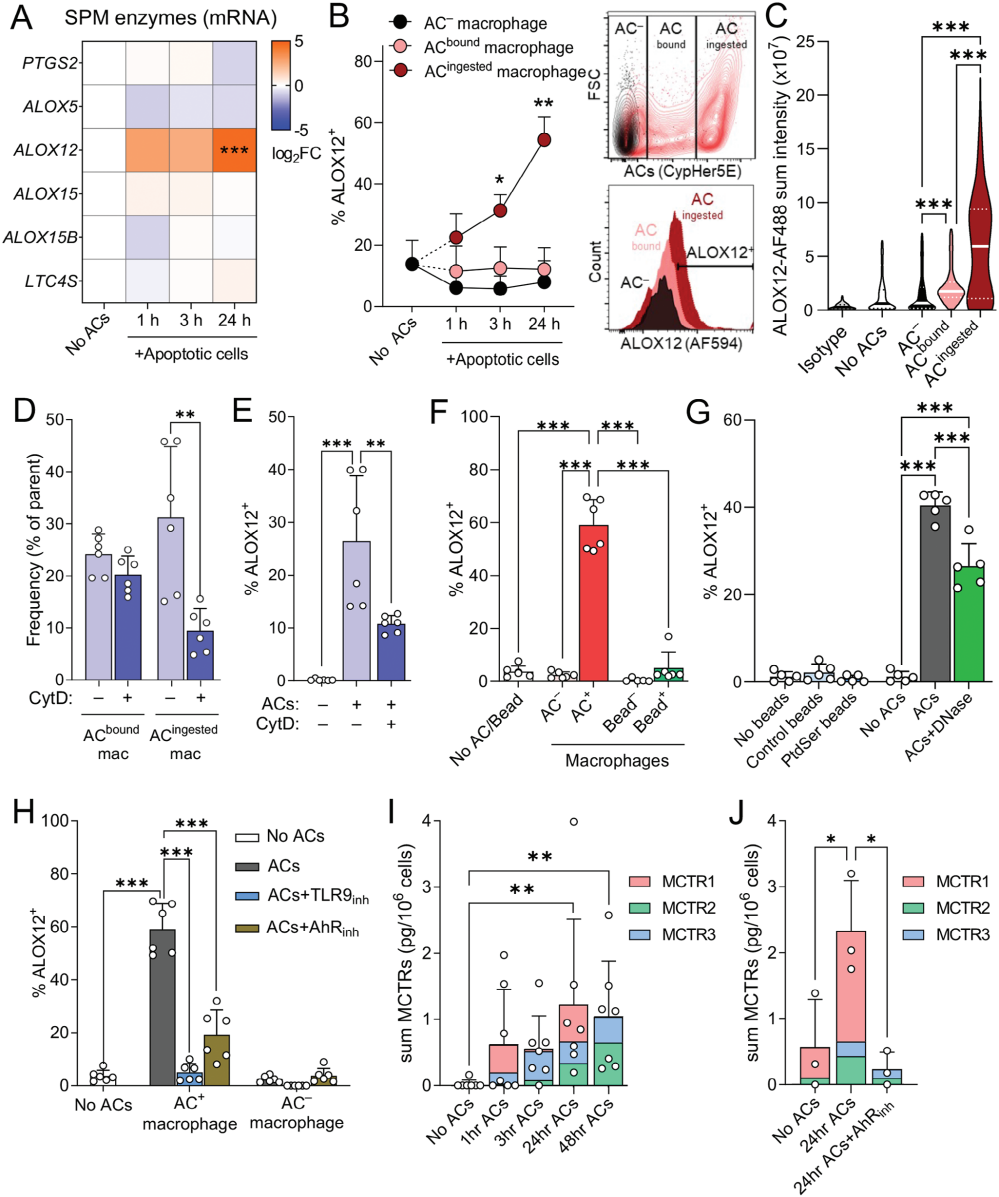
AC‐derived DNA upregulates ALOX12 expression in efferocytosing macrophages. A) Gene expression analysis for specialized pro‐resolving mediator (SPM) biosynthetic enzymes in human primary monocyte‐derived macrophages (MoDMs) incubated with ACsat 3:1 AC:macrophage ratio for indicated times as determined by RT‐qPCR. N = 6 human donors. B) Flow cytometry analysis of the percentage of ALOX12^+^ cells in AC‐negative macrophages (“AC^–^ macrophage”; negative for CypHer5E‐ACs), compared to macrophages that had either: bound but not internalized ACs (“AC^bound^ macrophage”; low intensity for CypHer5E‐ACs) or macrophages that had internalized ACs (“AC^ingested^ macrophage”; high intensity for CypHer5E‐ACs) after efferocytosis of CypHer5E‐labeled ACs (3:1 AC:macrophage ratio) by human MoDM for indicated amounts of time. Insets show representative contour plot for AC^–^, AC^bound^, and AC^ingested^ macrophage gating and corresponding histograms for ALOX12 MFI. N = 3 human donors. C) Fluorescence microscopy analysis of human MoDM stained with ALOX12 antibody followed by Alexa Fluor 488‐conjugated secondary antibody. Macrophages were classified into “AC‐“ (no associated AC), “AC^bound^” (AC signal within a 5 µm zone of interest around a macrophage), or “AC^ingested^” (high intensity for CypHer5E that overlaps with macrophage). Horizontal lines inside violin plots indicate median (solid) and quartiles (dashed); N = 5 human donors. D) Flow cytometry analysis of the percentage of AC^bound^ and AC^ingested^ macrophages in human MoDM cultures pre‐treated with or without 10 µm cytochalasin D for 2 h followed by efferocytosis of CypHer5E‐labelled ACs (3:1 AC:macrophage ratio) for 24 h. E) Flow cytometry analysis of the percentage of ALOX12^+^ macrophages in human MoDM cultures pre‐treated with or without 10 µm cytochalasin D for 2 h followed by efferocytosis of CypHer5E‐labelled ACs (3:1 AC:macrophage ratio) for 24 h. (F,G) Flow cytometry analysis of the percentage ALOX12^+^ cells in human MoDM; F) Comparison of ALOX12 frequency in macrophages efferocytosing CypHer5E‐labelled ACs (3:1 AC:macrophage ratio) versus macrophages phagocytosing 4 µm FluoSpheres Y/G polystyrene beads; G) Comparison of ALOX12 frequency in macrophages that have phagocytosed 5 µm uncoated Control polystyrene beads or phosphatidylserine (PtdSer)‐coated polystyrene beads, versus macrophages that have efferocytosed CypHer5E‐labelled ACs (3:1 AC:macrophage ratio) that were either untreated or pre‐treated with 100 µg mL DNase I for 1 hr prior to the assay. N = 6 human donors. H) Flow cytometry analysis of the percentage ALOX12^+^ cells in human MoDM after 24 h of efferocytosis of CypHer5E‐labelled ACs by macrophages pre‐treated with DMSO vehicle, TLR9 inhibitor (1 µg mL^−1^ ODN INH‐18), or Aryl hydrocarbon receptor (AhR) inhibitor (5 µm CH‐223191) for 1 h. N = 6 human donors. I) Quantification of endogenous MCTR levels using LC‐MS/MS in human MoDMs incubated with ACs at 3:1 AC:macrophage ratio for indicated intervals. N = 7 human donors. J) Quantification of endogenous MCTR levels using LC‐MS/MS in human MoDMs pre‐treated with AhR inhibitor (5 µm CH‐223191) for 1 h, followed by incubation with ACs at 3:1 AC:macrophage ratio for 24 hrs. N = 3 human donors. Data are shown as mean log_2_ fold change (for A) or as mean ± SD (B,D‐J). Statistical significance was determined using one‐way ANOVA (E,I,J) or two‐way ANOVA (A,C,D,F,G,H) with Holm‐Sidak multiple comparison correction, or Kruskal‐Wallis test with Dunn's post hoc multiple comparison correction (B). *p <0.05, **p <0.01, ***p <0.001 versus “No ACs” control (for A,B) or as indicated in the graphs.

To determine whether AC uptake regulates SPM formation we first screened for the expression of key biosynthetic enzymes responsible for SPM production in human monocyte‐derived macrophages incubated with ACs. At the transcript level, only *Alox12* expression was significantly upregulated in cells incubated with ACs (**Figure** **1**A). Flow cytometric evaluation corroborated these findings demonstrating that ALOX12 expression was rapidly upregulated in efferocytosing macrophages, reaching statistical significance within 3 h of AC addition (Figure 1B). Notably, this induction of ALOX12 was specific for macrophages that had ingested an AC, as increased ALOX12 positivity was not observed in macrophages that had bound an AC but not ingested it (Figure 1B; “AC^bound^ macrophage”), nor in macrophages that had neither bound or ingested an AC (Figure 1B; “AC^–^ macrophage”). Similar results were obtained when evaluating ALOX12 expression in efferocytosing macrophages using immunofluorescence microscopy, with the highest expression of ALOX12 being found in those macrophages that had ingested an AC (Figure 1C). Furthermore, ALOX12 expression was not detected in the ACs themselves (Figure S1A, Supporting Information).

During efferocytosis, macrophages use a variety of cell surface and intracellular receptors to recognize AC‐derived signals and activate appropriate downstream processes.^[^ ^2^ ^]^ To determine which signaling pathways are involved in ALOX12 upregulation during efferocytosis, we first blocked actin polymerization in macrophages using cytochalasin D to prevent AC ingestion.^[^ ^16^ ^]^ Cytochalasin D pre‐treatment prior to AC addition significantly reduced the frequency of macrophages that had ingested an AC, while leaving the frequency of macrophages that had bound but not ingested an AC unchanged (Figure 1D). Concomitantly, blocking AC ingestion in this manner significantly reduced the number of ALOX12 positive macrophages (Figure 1E). On the other hand, uptake of inert polystyrene beads did not upregulate ALOX12 expression to the extent that AC uptake did (Figure 1F; Figure S1B,C, Supporting Information). Moreover, coating polystyrene beads with phosphatidylserine to mimic AC membrane composition and activate cell surface efferocytosis receptors on macrophages failed to induce ALOX12 expression (Figure 1G). Together, these data show that complete AC ingestion is required for ALOX12 induction during efferocytosis.

Recent studies observed that DNA complexes coming from ingested ACs are recognized by toll‐like receptor 9 (TLR9) in the phagolysosome of efferocytosing macrophages.^[^ ^17^ ^]^ AC DNA recognition by TLR9 in turn leads to activation of the nuclear receptor Aryl hydrocarbon receptor (AhR) , ^[^ ^17^ ^]^ which functions as a transcription factor that can drive the expression of ALOX12 in other cell types.^[^ ^18^ ^]^ We therefore assessed the potential role of the TLR9‐AhR signaling axis in ALOX12 induction in efferocytosing macrophages. Pre‐incubation of ACs with DNase I, to remove exposed AC‐derived DNA complexes (Figure S1D, Supporting Information), resulted in reduced ALOX12 induction in efferocytosing macrophages (Figure 1G). Similarly, pre‐treatment of macrophages with inhibitors of either TLR9 or AhR significantly decreased ALOX12 induction in efferocytosing macrophages (Figure 1H). Taken together, these results support a role for AC‐derived DNA complexes in the upregulation of ALOX12 via activation of TLR9 and AhR signaling.

### 2.2 ALOX12 Metabolizes Docosahexaenoic Acid to Produce MCTRs in Efferocytosing Macrophages

ALOX12 catalyzes the formation of the MaR and MCTRs via the conversion of DHA.^[^ ^12^, ^19^, ^20^ ^]^ To evaluate the link between these mediators and the regulation of continual efferocytosis, we assessed their levels in efferocytosing macrophages using liquid chromatography coupled to tandem mass spectrometry (LC‐MS/MS) comparing signals obtained with standard material (Figure S2A–C, Supporting Information) with that from biological samples (Figure S2D,E, Supporting Information). Incubation of human monocyte‐derived macrophages with ACs led to a biphasic increase in MCTR1 at 1 and 24 h (Figure 1I). MCTR1 can subsequently be converted into MCTR2 and MCTR3 by successive enzymatic reactions (Figure S3A, Supporting Information).^[^ ^19^ ^]^ Consistent with this notion we found increased MCTR2 and MCTR3 levels at 3 and 48 h after AC addition (Figure 1I). Moreover, blocking the AC‐induced upregulation of ALOX12 by inhibiting AhR downregulated MCTR production in incubations of macrophage with AC (Figure 1J). Intriguingly, we observed that MCTRs were selectively upregulated in efferocytosing macrophages, since evaluation of Maresin (MaR)1 and MaR2 – additional pro‐efferocytic products of human ALOX12 (Figure S3A, Supporting Information) – demonstrated a downward trend with longer AC incubation times for MaR1 (Figure S3B, Supporting Information), while MaR2 was not identified in these experiments. These results suggest that human monocyte‐derived macrophages upregulate ALOX12 expression via AhR to preferentially increase MCTR production during efferocytosis.

**Figure 2:**
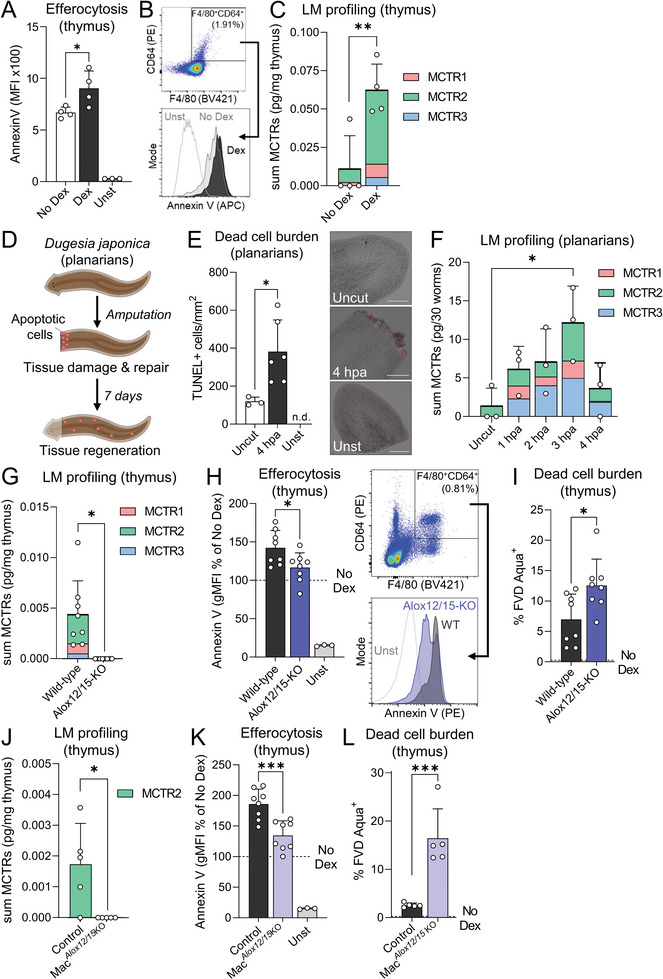
ALOX12 metabolizes docosahexaenoic acid to produce MCTRs in efferocytosing macrophages, while loss of MCTR formation abrogates the efferocytic ability of macrophages at sites of high AC burden. A–C) In vivo dexamethasone‐induced thymus apoptosis model with *i.p*. injection of 250 µg dexamethasone (Dex) or DMSO Vehicle (Veh) control for 18 h; (A) Flow cytometry analysis of Annexin V+ AC median fluorescence intensity (MFI) in F4/80^+^CD64^+^ thymic macrophages. Unst = unstained control; (B) Representative dot plots for F4/80^+^CD64^+^ thymic macrophage gating and histograms of Annexin V median fluorescence intensity (MFI); (C) Quantification of endogenous MCTR levels using LC‐MS/MS in Dex‐injured thymus. N = 4 mice per group. (D–F) Surgical injury‐induced tissue regeneration model using the planarian flatworm *Dugesia japonica;* (D) Schematic overview of experimental model; (E) Quantification of ACs by TUNEL assay in *D. japonica* planarians uninjured or 4 h post‐amputation (hpa). Unst = unstained control. Representative images of TUNEL signal (red) overlayed on brightfield images of planarians are shown in inset micrographs, where scale bar = 200 µm. N = 3 (Uncut) or 6 (4 hpa) planarians per group; (F) Quantification of endogenous MCTR levels using LC‐MS/MS in *D. japonica* planarians uninjured or surgically‐injured tissue sections at 1 to 4 h post‐amputation (hpa). N = 3 independent replicates per group comprised of 30 planarians per replicate. (G–I) In vivo dexamethasone‐induced thymus apoptosis model with *i.p*. injection of 250 µg dexamethasone or DMSO Vehicle (No Dex) control in Alox12/15‐KO or wild‐type C56BL/6J mice for 18 h; (G) Quantification of endogenous MCTR levels using LC‐MS/MS. (H) Flow cytometry analysis of Annexin V+ AC geometric mean fluorescence intensity (gMFI) in F4/80^+^CD64^+^ thymic macrophages, with mice that were not treated with dexamethasone indicated by “No Dex” horizontal dashed line. Unst = unstained control sample. Insets show representative dot plots for F4/80^+^CD64^+^ thymic macrophage gating and histograms of Annexin V MFI; (I) Flow cytometry analysis of the poportion of FVD Aqua^+^ dead cells in the thymus, with mice that were not treated with dexamethasone indicated by “No Dex” horizontal dashed line. The “No Dex” and “Unst” groups are shared between panels H & K and I & L. N = 8 mice per group. J‐L) In vivo dexamethasone‐induced thymus apoptosis model with *i.p*. injection of 250 µg dexamethasone or DMSO Vehicle (No Dex) control in Mac^Alox12/15 KO^ or Alox12/15‐floxed (Control) littermates for 18 h; (J) Quantification of endogenous MCTR levels using LC‐MS/MS. MCTR1 and MCTR3 were below limits of quantitation; (K) Flow cytometry analysis of Annexin V+ AC geometric mean fluorescence intensity (gMFI) in F4/80^+^CD64^+^ thymic macrophages, with mice that were not treated with dexamethasone indicated by “No Dex” horizontal dashed line. Unst = unstained control sample; (L) Flow cytometry analysis of the proportion of FVD Aqua^+^ dead cells in the thymus, with mice that were not treated with dexamethasone indicated by “No Dex” horizontal dashed line. The “No Dex” and “Unst” groups are shared between panels H & K and I & L. N = 8 mice per group. Data are shown as mean ± SD. Statistical significance was determined using two‐tailed Student's *T*‐test (A,C,E,H,I,K,L), one sample Wilcoxon signed‐rank test (G,J), or one‐way ANOVA with Holm‐Sidak multiple comparison correction (F). *p <0.05, **p <0.01, ***p <0.001 as indicated.

Continual efferocytosis is known to play a central role in the maintenance of tissue homeostasis during periods of high AC burden.^[^ ^7^ ^]^ To determine whether MCTR formation was upregulated in such settings, we used an established thymus injury mouse model in which extensive thymocyte apoptosis is induced by intraperitoneal dexamethasone (Dex) injection.^[^ ^7^ ^]^ In line with published studies,^[^ ^7^ ^]^ we observed that Dex treatment significantly increased the number of efferocytosing macrophages in the thymus (**Figure** **2**A,B). Using LC‐MS/MS, we identified all three MCTRs in these tissues (Figure S2E, Supporting Information). Furthermore, we found that MCTR levels were significantly increased in tissues from mice treated with Dex when compared with those from vehicle control‐treated mice (Figure 2C). To explore whether such MCTR production at sites of high AC burden was retained in other injury settings, we identified and evaluated the production of these mediators in surgically injured planarian flatworms (Figure 2D; Figure S2F, Supporting Information). In this model organism, complete tissue regeneration is dependent on successive waves of AC generation and clearance, with the first wave of ACs peaking 3 to 4 h post‐amputation (Figure 2E).^[^ ^21^ ^]^ Here, we found a time‐dependent increase in MCTRs, with peak levels observed 3 h post‐amputation that then declined by 4 h post‐amputation (Figure 2F). This observation is consistent with the time frame of post‐injury AC generation and clearance in planarians (peaking 3–4 hpa),^[^ ^21^ ^]^ as well as with the notion that MCTR, like other autacoids, are locally‐acting signaling molecules that are synthesized and degraded in a timely manner according to tissue needs.^[^ ^22^ ^]^ These findings also suggest that MCTRs are evolutionarily conserved signals produced at sites of high AC burden.

### 2.3 Loss of MCTR Formation Abrogates the Ability of Macrophages to Uptake ACs at Sites of High AC Burden

To detail the link between MCTR production and the efferocytic ability of macrophages at sites of high AC burden, we evaluated AC clearance in mice lacking the initiating enzyme in the MCTR biosynthetic pathway, Alox12/15 (Figure S3A, Supporting Information).^[^ ^19^ ^]^ In thymi from Dex‐treated Alox12/15‐knockout (KO) mice, we observed a significant downregulation in MCTR production (Figure 2G), which was linked with a significant decrease in efferocytosis by thymus macrophages (Figure 2H), as well as a significant increase in dead cell burden (Figure 2I) when compared to Alox12/15‐expressing wild‐type (WT) mice.

To establish the role of macrophage‐derived MCTRs in AC clearance in vivo, we next generated a transgenic mouse line in which expression of Alox12/15 is specifically abrogated in macrophages by crossing Alox12/15 floxed mice with mice expressing Cre‐recombinase under the control of the *Cx3cr1* gene promotor (Mac*^Alox12/15^* ^KO^), which resulted in reduced Alox12/15 expression (Figure S3C,D, Supporting Information). In these Mac*^Alox12/15^* ^KO^ mice, we observed a significant reduction in MCTR levels following Dex administration(Figure 2J), as well as a significantly reduced ability of thymic macrophages to clear ACs compared to control mice (Figure 2K,L), replicating the observations made in global Alox12/15‐KO mice (Figure 2G–I). Together, these findings demonstrate that MCTR production in macrophages is upregulated at sites of high AC burden, and loss of their production is linked with a reduced ability of macrophages to clear ACs.

### 2.4 MCTRs Promote Continual Efferocytosis in Macrophages

**Figure 3:**
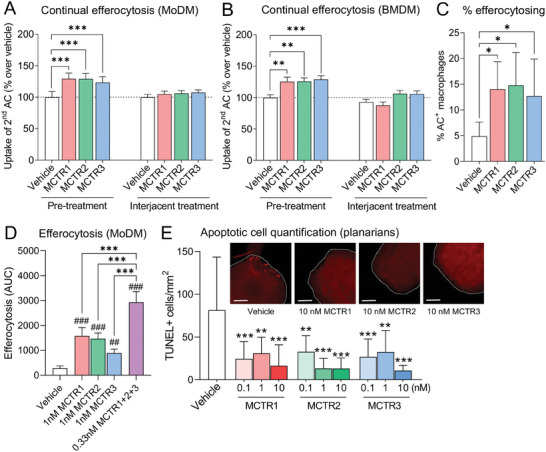
MCTRs promote continual efferocytosis in macrophages. A,B) Continual efferocytosis of successive rounds of ACs by (A) human MoDMs, or (B) mouse BMDM. Macrophages were loaded with phrodo Red‐labeled ACs (3:1 ratio) for 1 h followed by washing and a 2 h rest period prior to incubation with CypHer5E‐labeled ACs (3:1 ratio) for 3 h. MoDMs and BMDMs were incubated with 1 nm MCTR1, MCTR2, MCTR3, or Vehicle (0.05% EtOH) for 15 min prior to first round of pHrodo Red‐labeled ACs (“Pre‐treatment”) or 15 min prior to the second round of CypHer5E‐labeled ACs (“Interjacent treatment”). N = 5 human donors (for A), or N = 3 mice (for B). C) Percentage of AC‐positive macrophages 45 min after addition of phrodo Red‐labeled ACs (3:1 ratio) to MoDMs incubated with 1 nm MCTR1, MCTR2, MCTR3, or Vehicle (0.05% EtOH) for 15 min prior to AC addition. N = 7 human donors. D) Efferocytosis of phrodo Red‐labeled ACs (3:1 ratio) by MoDMs incubated with Vehicle (0.05% EtOH), 1 nm MCTR1, MCTR2, or MCTR3 individually, or 0.33 nm of MCTR1, MCTR2, and MCTR3 combined for 15 min. The Vehicle, MCTR1, MCTR2, and MCTR3 groups are shared with Figure 6J. N = 3 human donors. E) Quantification of ACs by TUNEL assay in surgically injured *D. japonica* planarians incubated with 0.1‐10 nm MCTR1, MCTR2, MCTR3, or Vehicle (Planaria water containing 0.05% EtOH) for 4 h. Representative images are displayed below bar graphs, where white dotted lines delineate Planaria. Scale bar = 200 µm. N = 8 planarians per group. Data are shown as mean ± SD. p‐values were calculated using two‐way ANOVA with Holm‐Sidak multiple comparison correction. *p <0.05, ^##/^**p <0.01, ^###/^***p <0.001 as indicated or versus Vehicle control (for D and E).

As MCTRs were upregulated at sites of high AC burden and in efferocytosing macrophages, we next sought to determine whether MCTRs regulated continual efferocytosis. Using high‐content imaging, we found that incubation of human macrophages with each of the MCTRs prior to the addition of ACs (“Pre‐treatment”) significantly enhanced their ability to ingest subsequent rounds of ACs (**Figure** **3**A). In these studies, we incubated macrophages with 1 nmole L^−1^ of each of the MCTRs separately. The identity and concentration of each MCTR was validated by LC‐MS/MS (Figure S2A–C, Supporting Information) and UV spectroscopy (Figure S3E, Supporting Information). Moreover, the concentration used is in line with the quantities of these mediators produced by efferocytosing macrophages, whereby in a 2 mL culture volume containing 1 × 10^6^ cells we detected 0.2–0.5 pg of each MCTR (Figure 1I). Given a molecular weight of 650 g/mol for MCTR1, 521 g/mol for MCTR2, and 464 g/mol for MCTR3 (Figure S2A–C, Supporting Information), such quantities equate to ≈0.15–0.54 nmole L^−1^of each MCTR in these cultures. Intriguingly, addition of MCTRs after the first round of ACs, but before the addition of the second round of cells (“Interjacent treatment”) had no significant effect (Figure 3A). Similar results were obtained using mouse bone marrow‐derived macrophages (BMDM), where MCTR pre‐treatment significantly enhanced continual efferocytosis, while interjacent treatment did not (Figure 3B). MCTR pre‐treatment also significantly enhanced the proportion of macrophages performing efferocytosis (Figure 3C), while pre‐treatment with a combination of all three MCTRs synergistically enhanced efferocytosis beyond levels seen for each of the MCTRs individually (Figure 3D). Together, these findings suggest that MCTRs upregulate continual efferocytosis by priming macrophages for initial AC uptake.

To determine whether the ability of MCTRs to induce continual efferocytosis was retained in vivo, we used the planarian flatworm surgical injury model. Here, flatworms were first injured to induce apoptosis at the surgical site, ^[^ ^21^ ^]^ then treated with one of the MCTRs at an interval where AC numbers are known to reach a maximum (3 h post injury^[^ ^21^ ^]^), and the number of TUNEL‐positive cells enumerated as a measure of free ACs. In these experiments we observed a significant reduction in the number of TUNEL‐positive cells in flatworms treated with MCTR1, MCTR2, or MCTR3 when compared with those that were incubated in vehicle control (Figure 3E). These findings, together with the observations that AC uptake leads to enhanced MCTR production (Figure 1I), suggest that macrophages upregulate MCTRs in response to initial AC uptake to rapidly and transiently boost continual efferocytosis capability.

### 2.5 12‐Lipoxygenase‐Expressing Macrophages Upregulate Efferocytosis in Neighboring Cells

**Figure 4:**
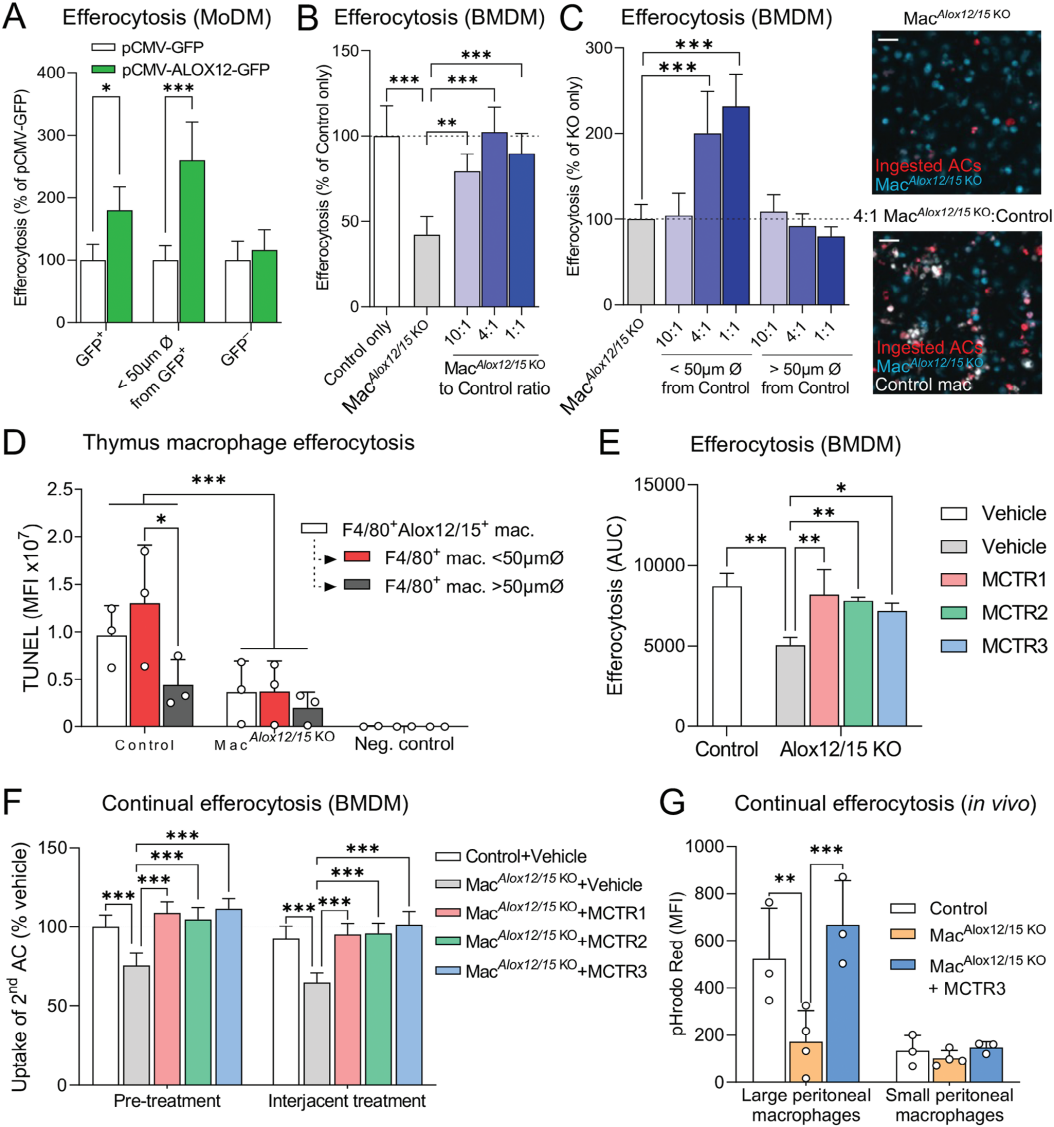
12‐lipoxygenase‐expressing macrophages upregulate efferocytosis in neighboring cells, while defective continual efferocytosis in macrophages lacking 12‐lipoxygenase activity is rescued by MCTR treatment. A) Efferocytosis of phrodo Red‐labeled ACs (3:1 AC:macrophage ratio) by MoDMs transfected with plasmids encoding for either human ALOX12 C‐terminally fused to green fluorescent protein (pCMV‐ALOX12‐GFP) or GFP control vector. Efferocytosis was measured over time in GFP^+^ cells, in cells that were within a 50 µm diameter around GFP^+^ cells (“Within 50 µm Ø around GFP^+^”), or in cells that were neither GFP^+^ or near GFP^+^ cells (GFP^–^) within the same well using high‐content imaging. N = 3 human donors. B) Efferocytosis of phrodo Red‐labeled ACs (3:1 AC:macrophage ratio) by mouse BMDM from macrophage‐specific (Cx3cr1:cre‐driven) *Alox12/15*‐deficient mice (Mac*^Alox12/15^* ^KO^) or *Alox12/15*‐floxed littermates (Control) either by themselves or co‐cultured at indicated Mac*^Alox12/15^* ^KO^:Control ratios measured over time using high‐content imaging. N = 4 mice. C) Efferocytosis of CypHer5E‐labeled ACs (3:1 AC:macrophage ratio) by CellBrite Green‐labeled Mac*^Alox12/15^* ^KO^ BMDM co‐cultured with PKH26‐labeled Control BMDM at indicated Mac*^Alox12/15^* ^KO^:Control cell ratios, where efferocytosis by CellBrite Green^+^ KO BMDMs was assessed for cells that were within a 50 µm diameter around PKH26^+^ Control BMDMs (“<50 µm from Control”) versus cells that were further away (“>50 µm from Control”). N = 3 mice. Micrographs on the right show representative fluorescence microscopy images from “Mac*^Alox12/15^* ^KO^” (top) and “4:1 Mac*^Alox12/15^* ^KO^ to Control” groups at 3 h post‐AC addition. Scale bar = 50 µm. D) Fluorescence microscopy analysis of thymi from Mac*^Alox12/15^* ^KO^ or *Alox12/15*‐floxed littermates (Control) that were *i.p*. injected with 250 µg dexamethasone for 18 h. TUNEL^+^ AC efferocytosis by F4/80^+^Alox12/15^+^ thymic macrophages were compared to F4/80^+^ macrophages within a 50 µm diameter area around F4/80^+^Alox12/15^+^ thymic macrophages (<50 µmØ) versus F4/80^+^ macrophages outside of such an area (>50 µmØ). TUNEL‐only stained sections included as negative control (“Neg. control”). N = 3 mice per group. E) Efferocytosis of phrodo Red‐labeled ACs 3:1 AC:macrophage ratio) by BMDM pre‐incubated 15 min with 1 nm MCTR1, MCTR2, MCTR3, or Vehicle (0.05% EtOH). N = 3 mice per group. F) Continual efferocytosis of successive rounds of ACs by mouse BMDM. BMDMs from Mac*^Alox12/15^* ^KO^ mice or Alox12/15‐floxed littermates (Control) were loaded with phrodo Red‐labeled ACs (3:1 ratio) for 1 h followed by washing and a 2 h rest period prior to loading with CypHer5E‐labeled ACs (3:1 ratio) for 3 h. BMDMs were incubated with 1 nm MCTR1, MCTR2, MCTR3, or Vehicle (0.05% EtOH) for 15 min prior to first round of phrodo Red‐labeled ACs (“Pre‐treatment”) or after the first round of ACs but prior to the second round of CypHer5E‐labeled ACs (“Interjacent treatment”). N = 3 mice per group. G) In vivo continual efferocytosis of phrodo Red‐labeled ACs by CD3^−^CD19^−^SiglecF^−^CD102^+^CD11b^hi^F4/80^hi^ large peritoneal macrophages or CD3^−^CD19^−^SiglecF^−^CD102^−^CD11b^med^F4/80^lo^ small peritoneal macrophages in Mac*^Alox12/15^* ^KO^ mice or Alox12/15‐floxed littermates (Control). Mice were *i.p*. injected with 3.33 × 10^6^ unlabeled ACs in 100 µL PBS, followed 2 h later by 10 ng MCTR3 and 3.33 × 10^6^ phrodo Red‐labeled ACs in 100 µL PBS each. Peritoneal lavages were harvested 45 min after injection of MCTR3 and the second round of ACs, stained with fluorescently conjugated antibodies against the peritoneal macrophage markers mentioned above, and analysed by flow cytometry. N = 3 mice for Control and Mac^Alox12/15 KO^ groups and N = 4 mice for Mac^Alox12/15 KO^ +MCTR3 group. Data are shown as mean ± SD. Statistical significance was determined using two‐way ANOVA with Holm‐Sidak multiple comparison correction. *p <0.05, **p <0.01, ***p <0.001 as indicated in the graphs.

Since we observed that MCTRs increased the number of efferocytosing cells (Figure 3C), we queried whether efferocytosing ALOX12‐positive cells instruct surrounding macrophages, via the formation of soluble mediators including MCTRs, to take up ACs. To test this hypothesis, we transfected human macrophages with either a vector expressing ALOX12 tagged with green fluorescent protein (GFP), or a control vector expressing GFP only. Evaluation of efferocytosis in these transfected cells demonstrated that cells overexpressing ALOX12 were significantly better at efferocytosis than cells transfected with the control vector (**Figure** **4**A; “GFP^+^” group). Notably, this efferocytosis‐boosting effect was also observed in macrophages that were not transfected with the ALOX12‐GFP vector, but were in close proximity —, i.e., within the one average macrophage cell length of 21 µm — to ALOX12‐GFP positive macrophages (Figure 4A; “<50 µm Ø from GFP^+^” group). On the other hand, macrophages that were neither ALOX12‐GFP positive nor in the vicinity of such a cell did not show enhanced efferocytosis (Figure 4A; “GFP^–^” group).

To further explore the role of ALOX12 in coordinating macrophage efferocytosis, we obtained BMDM from Mac*^Alox12/15^* ^KO^ mice. These cells displayed a defect in their ability to perform efferocytosis, an effect that was rescued when these cells were co‐cultured with Control macrophages from Alox12/15‐expressing mice (Figure 4B). Moreover, this beneficial effect of Alox12/15‐expressing Control macrophages only extended to those Mac*^Alox12/15^* ^KO^ macrophages that were within close proximity to a Control macrophage (Figure 4C; “<50 µm Ø from Control”). Results that were in line with those obtained with ALOX12‐overexpressing human macrophages.

Next, we validated the above in vitro findings by assessing AC clearance in vivo using the Dex‐induced thymus injury mouse model (summarized in Figure 1A). We observed increased thymus cellularity (Figure S4A, Supporting Information) and reduced intracellular Annexin V staining in F4/80^+^CD64^+^ thymic macrophages from Mac*^Alox12/15^* ^KO^ mice (Figure 2K) — two established markers of impaired efferocytosis — as well as reduced Alox12/15 expression (Figure S4B, Supporting Information). These observations were further corroborated by reduced TUNEL staining associated with F4/80^+^ macrophages in Mac*^Alox12/15^* ^KO^ mice (Figure 4D), another indicator of reduced efferocytosis.^[^ ^9^ ^]^ Furthermore, this analysis showed that, in *Alox12/15*‐expressing Control mice, efferocytosis was significantly higher in macrophages that were either positive for Alox12/15 (Figure 4D; “F4/80^+^Alox12/15^+^ mac”) or within close proximity of such a cell (Figure 4D; “F4/80^+^ mac <50 µm Ø”) — an effect that was completely absent in Mac*^Alox12/15^* ^KO^ mice or negative control samples in which macrophages were left unstained (Figure 4D). Taken together, these results demonstrate that human monocyte‐derived macrophages expressing ALOX12 and mouse monocyte‐derived or thymic macrophages expressing Alox12/15 are competent efferocytes that positively regulate the ability of surrounding macrophages to clear ACs.

### 2.6 MCTR Treatment Rescues Defective Continual Efferocytosis in Macrophages Lacking 12‐Lipoxygenase Activity

Given our observations that ALOX12‐expressing human macrophages boost efferocytosis in surrounding macrophages, and that mouse macrophages lacking Alox12/15 have a reduced efferocytosis capacity that can be rescued by co‐culture with Alox12/15‐expressing macrophages, we hypothesized that ALOX12‐derived MCTRs might be responsible for the above effects. To that end, we assessed the effects of MCTRs on efferocytosis in mouse BMDM lacking Alox12/15. These experiments showed that reduced efferocytosis in Alox12/15‐deficient macrophages was rescued by incubation with each of the MCTRs to an extent that was comparable to that observed in Control Alox12/15‐expressing cells (Figure 4E). MCTR treatment also rescued defective continual efferocytosis in Alox12/15‐deficient macrophages, both when applied as a pre‐treatment prior to first contact with ACs (Figure 4F; “Pre‐treatment”), as well as when applied in between successive rounds of ACs (Figure 4F; “Interjacent treatment”).

Defective continual efferocytosis in Alox12/15‐deficient macrophages was also observed in vivo, as we found a significant reduction in the ability of large peritoneal macrophages from Mac*^Alox12/15^* ^KO^ mice to uptake multiple rounds of ACs when compared to Alox12/15‐expressing Control mice (Figure 4G). Intriguingly, add‐back of MCTR3 rescued the defect in continual efferocytosis by large peritoneal macrophages from Mac*^Alox12/15^* ^KO^ mice (Figure 4G). Notably, these effects were absent in small peritoneal macrophages, which have previously been reported to be poor efferocytes that express low levels of Alox12/15.^[^ ^23^ ^]^ These observations suggest that the pro‐efferocytic activities of MCTR3, and potentially other MCTRs, may be linked with the biology of the specific macrophage subset.

Taken together, these results corroborate our in vitro observations, demonstrating a role for MCTRs in the localized continual efferocytosis‐boosting effects of human ALOX12‐positive and mouse Alox12/15‐positive macrophages. Moreover, the above findings, together with our previous observation that interjacent MCTR treatment failed to upregulate continual efferocytosis (Figure 3A,B), support the hypothesis that efferocytosis in a subset of macrophages triggers the endogenous production of MCTRs, which then prime nearby macrophages to clear additional ACs. Once this pathway is initiated, it becomes self‐perpetuating and is not sensitive to subsequent addition of MCTRs.

### 2.7 Phosphoproteomics of MCTR‐Treated Macrophages Highlights Changes in Rho GTPase and Glycolysis Pathways

**Figure 5:**
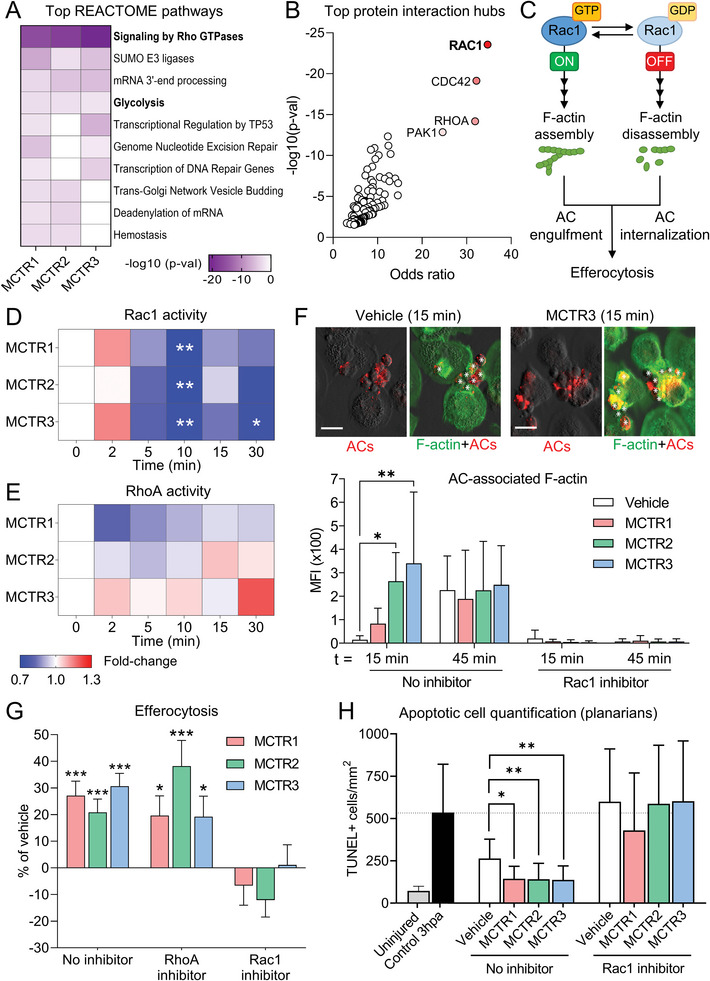
MCTRs regulate macrophage Rac1 activity and enhance initial AC uptake in a Rac1‐dependent manner. A,B) Phosphoproteomic profiling of human MoDMs treated with 1 nm MCTR1, MCTR2, MCTR3, or Vehicle (0.05% EtOH) for 5 min or 15 min. N = 4 human donors. Datasets for 5 min and 15 min MCTR treatment were combined prior to downstream analyses. (A) REACTOME pathway analysis showing top pathways associated with significantly differentially phosphorylated peptides. (B) Scatter plot of p‐values and odd ratios from Enrichr protein‐protein interaction hub analysis of significantly differentially phosphorylated peptides associated with the REACTOME pathway “Signaling by Rho GTPases”. C) Schematic overview of Rac1 activity cycling required for successful efferocytosis. (D‐E) Activity heatmap of the Rho GTPases Rac1 D) and RhoA (E) as measured by G‐LISA in human MoDMs incubated with 10 nm MCTR1, MCTR2, MCTR3, or Vehicle (0.05% EtOH) for indicated time periods. N = 6 human donors (for Rac1) or 2 human donors (for RhoA). F) (*Top*) Representative micrographs of Phalloidin‐stained macrophage F‐actin and phrodo Red‐labeled ACs. *(Bottom)* Quantification of AC‐associated F‐actin in Phalloidin‐stained MoDMs pre‐incubated with Rac1 inhibitor (100 µm NSC23766) or DMSO control (0.05% DMSO) for 20 h, followed by 1 nm MCTR1, MCTR2, MCTR3, or Vehicle (0.05% EtOH) for 15 min. MoDMs were then loaded with phrodo Red‐labeled ACs (3:1 ratio) for 15‐ or 45‐min. Scale bar = 20 µm. N = 3 human donors for 15 min time point; N = 5 human donors for 45 min time point. G) Efferocytosis of phrodo Red‐labeled ACs (3:1 ratio) by MoDMs pre‐incubated with Rac1 inhibitor (100 µm NSC23766), RhoA inhibitor (0.5 µg mL^−1^ CT04), or DMSO control (0.05% DMSO) for 20 h followed by 1 nm MCTR1, MCTR2, MCTR3, or Vehicle (0.05% EtOH) for 15 min. N = 7 human donors. H) Quantification of ACs by TUNEL assay in surgically injured *D. japonica* planarians incubated in Planaria water for 3 h followed by Rac1 inhibitor (100 µm NSC23766) or DMSO control (0.1% DMSO), combined with 10 nm MCTR1, MCTR2, MCTR3, or Vehicle (Planaria water containing 0.05% EtOH) for 1 h. N = 10‐11 planarians per group. Data are shown as mean –log_10_ p‐values (for A), as geometric mean fold change (for D‐E), or as mean ± SD (F‐H). p‐values were calculated using two‐way ANOVA with Holm‐Sidak multiple comparison correction. *p <0.05, **p <0.01, ***p <0.001 versus time point 0 (for D,E), Vehicle control (for G), or as indicated.

Recent studies demonstrate that priming events during or immediately after the uptake of the first ACs are central in facilitating continual efferocytosis.^[^ ^8^ ^]^ As we observed that MCTRs prime macrophages to facilitate this process, we next sought to determine the intracellular pathways activated by these mediators that lead to their efferocytosis‐enhancing effects. Given the observation that MCTRs rapidly upregulated the efferocytic capability of macrophages (within 15 min; Figure 3C), we speculated that their effects might be mediated by rapid changes in protein post‐translational modifications. To that end, we measured global protein phosphorylation changes in human macrophages treated with MCTR1, MCTR2, or MCTR3 using label‐free mass spectrometry as previously described.^[^ ^24^ ^]^ We then analyzed the data using Partial Least Squares Discriminant analysis (PLS‐DA), a type of multivariate analysis that reduces dataset dimensions and identifies the relationship between samples based on differences in phosphopeptide abundance. This analysis showed a clear separation between Vehicle control versus MCTR‐treated macrophage groups with a coefficient‐of‐determination (*R* ^2^) of 0.89 (Figure S5A, Supporting Information), indicating a strong ability of the model to explain the differences between these four groups. Moreover, there was a partial overlap between MCTR1, MCTR2, and MCTR3‐treated macrophages (Figure S5A, Supporting Information), indicative of both overlapping and unique actions of the three MCTRs. Closer inspection of this overlap identified 268 peptides that were significantly differentially‐phosphorylated compared to Vehicle control for at least two out of three MCTRs (Figure S5B, Supporting Information). Pathway analysis on this set of 268 overlapping phosphopeptides showed an enrichment for proteins involved in “signaling by Rho GTPases” (**Figure** **5**A). While further predictive analysis of the differentially‐phosphorylated peptides that fall under this pathway specifically identified the Rho GTPase Rac1 as the top activated hub protein in our dataset (Figure 5B). These results are in accord with published findings establishing a role for Rac1 in cytoskeletal remodeling required for macrophage chemotaxis^[^ ^25^ ^]^ and ACs engulfment, ^[^ ^15^ ^]^ and in counteracting efferocytosis‐inhibiting RhoA activation during efferocytosis.^[^ ^26^, ^27^ ^]^

Additionally, all three MCTRs significantly regulated phosphorylation of proteins involved in glycolysis (Figure 5A), another pathway previously shown to be critical for effective efferocytosis.^[^ ^10^ ^]^ Together, these results identify two pathways, Rac1 signaling and glycolysis, that are potentially required for MCTR‐mediated enhancement of efferocytosis.

### 2.8 MCTRs Regulate Macrophage Rac1 Activity and Enhance Initial AC Uptake in a Rac1‐Dependent Manner

During efferocytosis, Rac1 GTPase activity is switched on and off at distinct intervals to allow for build‐up and break‐down of actin filaments (F‐actin) to engulf ACs and subsequently internalize them (Figure 5C).^[^ ^15^ ^]^ Thus, we next investigated the effects of MCTRs on Rac1 signaling. Measuring Rac1 GTPase activity in the absence of ACs, we found that MCTRs caused a small initial increase in Rac1 activity that was swiftly followed by a strong decrease in activity, reaching statistical significance at 10 min after addition of each MCTR (Figure 5D). This pattern of Rac1 activity regulation by MCTRs was mirrored by changes in F‐actin formation, which initially increased and then trended down in MCTR‐treated macrophages when compared to Vehicle control‐treated cells (Figure S5C, Supporting Information). Intriguingly, we also observed that MCTRs did not directly regulate activity of the Rho GTPase family member RhoA (Figure 5E), which is described as a negative regulator of efferocytosis.^[^ ^26^, ^27^ ^]^

Next, we assessed the requirement for Rac1 activity in MCTR‐enhanced efferocytosis. A detailed investigation of AC uptake kinetics revealed that MCTR pre‐treatment increased the amount of F‐actin associated with bound ACs at early time points (Figure 5F), indicative of an increased readiness of MCTR‐treated macrophages to engulf ACs and consistent with the hypothesis that MCTRs rapidly prime macrophages for efferocytosis. Related to this observation, we found that MCTR treatment had no effect on the average number of ACs bound by each macrophage (Figure S5D, Supporting Information), but significantly increased the proportion of ACs that were ingested (Figure S5E, Supporting Information). These results suggest that MCTRs prime the internal efferocytic machinery of macrophages and not their ability to bind ACs in the first instance. Notably, all of the effects of MCTRs described above were absent upon Rac1 inhibition (Figure S5D,E, Supporting Information). Moreover, pre‐treatment of human macrophages with a Rac1 inhibitor completely abrogated the ability of MCTRs to enhance efferocytosis, while pharmacological RhoA inhibition had no such effect (Figure 5G).

Previous studies have shown that phosphoinositide 3‐kinase (PI3K)‐mediated regulation of Rac1 activity via the Rho GTPase activating proteins ARHGAP12 and ARHGAP25 is crucial for ingestion of large particles by phagocytes.^[^ ^28^ ^]^ Notably, ARHGAP12, ARHGAP25, and the PI3Kγ regulatory subunit PIK3R5 were found to be significantly differentially phosphorylated in our phosphoproteomic screen (Table S1, Supporting Information). Consistent with this observation, pre‐treatment of macrophages with a PI3Kγ‐specific inhibitor completely abrogated the ability of MCTRs to upregulate efferocytosis (Figure S5F, Supporting Information).

Given that the planarian flatworm species used in these studies (*Dugesia japonica*) express a well‐conserved Rac1 homolog,^[^ ^29^ ^]^ we next evaluated the role of Rac1 in mediating the activities of MCTRs on AC clearance in vivo. For this purpose, we evaluated AC clearance in surgically‐injured planarians treated with MCTRs in the presence or absence of a Rac1 inhibitor. These studies demonstrated that MCTR‐mediated decreases in AC burden were abrogated by Rac1 inhibition (Figure 5H). Together, these results point towards a critical role for Rac1 in mediating the efferocytosis‐priming effects of MCTRs.

### 2.9 Enhancement of Initial AC Uptake and Tissue Regeneration by MCTRs Depends on Increased Glycolytic Metabolism and Rac1‐Driven Glucose Uptake

**Figure 6:**
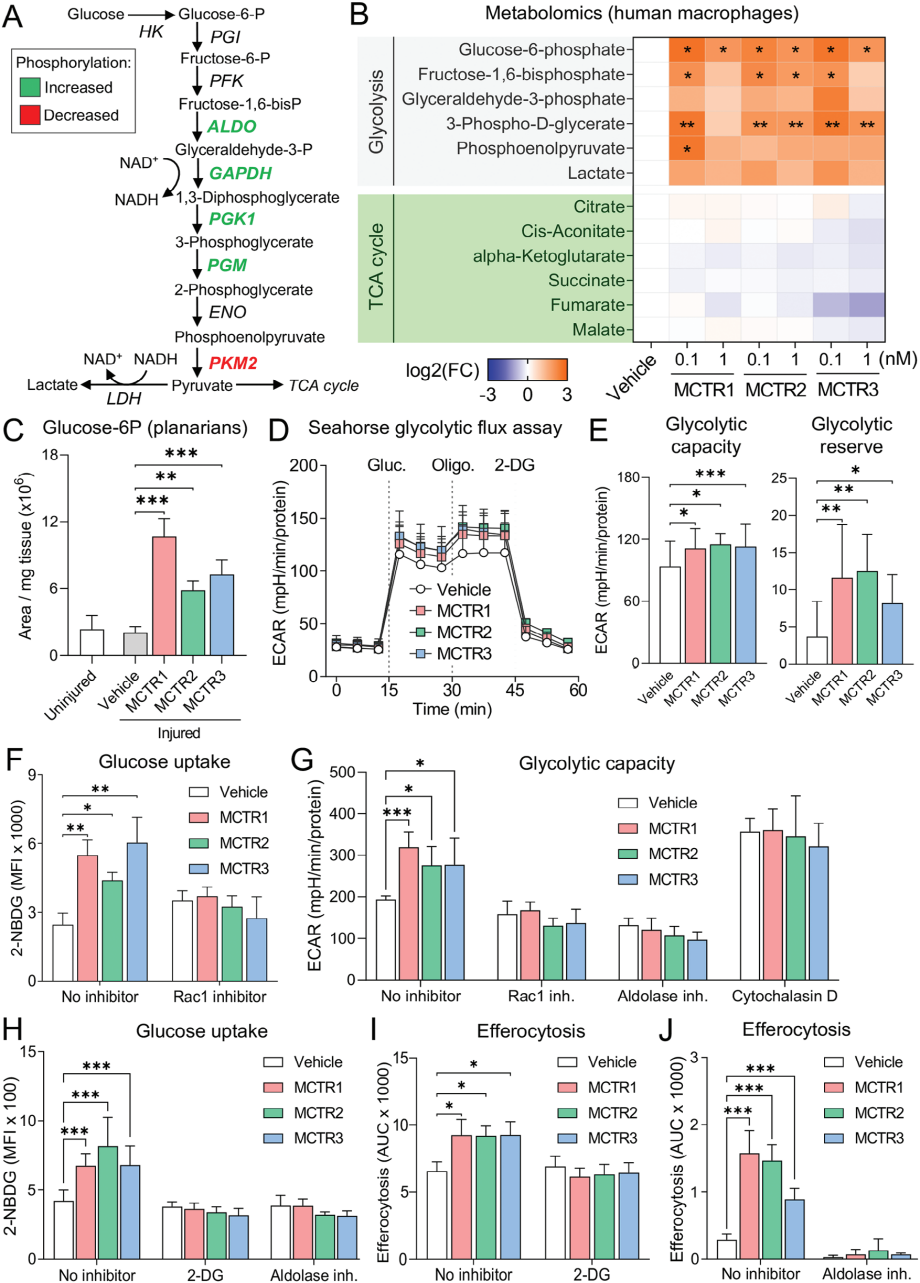
Enhancement of initial AC uptake by MCTRs depends on increased glycolytic metabolism and Rac1‐driven glucose uptake. A) Schematic representation of glycolysis pathway, with differentially phosphorylated enzymes from phosphoproteomic screen in described in Figure 5 and Figure S5 (Supporting Information) indicated in green (phosphorylation increased by MCTRs) or red (phosphorylation decreased by MCTRs). B) Heatmap of steady‐state levels of glycolysis and TCA cycle metabolites in MoDMs incubated with 0.1 or 1 nm MCTR1, MCTR2, MCTR3, or Vehicle (0.05% EtOH) for 1 h as determined by LC‐MS/MS. N = 4 human donors for glycolysis metabolites and N = 3 human donors for TCA cycle metabolites. C) Steady‐state levels of glucose‐6‐phosphate as determined by LC‐MS/MS in uninjured or surgically injured Planaria incubated with 10 nm MCTR1, MCTR2, MCTR3, or Vehicle (0.05% EtOH) for 3 h. N = 3 independent replicates per group (with 20 worms per replicate). D,E) Seahorse flux analysis of glycolytic activity in human MoDMs incubated with 1 nm MCTR1, MCTR2, MCTR3, or Vehicle (0.05% EtOH) for 1 h. N = 8 human donors. (D) Extracellular acidification rate (ECAR) in response to MCTR pre‐treatment followed by sequential glucose (Gluc), oligomycin (Oligo), and 2‐deoxyglucose (2‐DG) injections; (E) Glycolytic capacity and reserve parameters calculated from ECAR curves shown in D. F) Glucose uptake as measured by 2‐NBDG accumulation in glucose‐starved human MoDMs pre‐incubated with Rac1 inhibitor (100 µm NSC23766) for 20 h followed by 1 nm MCTR1, MCTR2, MCTR3, or Vehicle (0.05% EtOH) for 15 min. N = 6 human donors. G) Glycolytic capacity of human MoDMs pre‐treated with Rac1 inhibitor (100 µm NSC23766) for 18 h, or aldolase inhibitor (10 µm Aldometanib) or cytochalasin D (10 µm) for 2 h, followed by incubation with 1 nm MCTR1, MCTR2, MCTR3, or Vehicle (0.05% EtOH) for 1 h. Values were calculated from Seahorse flux analysis‐derived ECAR curves shown in Figure S6C (Supporting Information). N = 5 human donors. H) Glucose uptake as measured by 2‐NBDG accumulation in glucose‐starved human MoDMs pre‐incubated with 2‐DG (10 mm) or aldolase inhibitor (10 µm Aldometanib) for 2 h followed by 1 nm MCTR1, MCTR2, MCTR3, or Vehicle (0.05% EtOH) for 15 min. N = 5 human donors. I) Efferocytosis of phrodo Red‐labeled ACs (3:1 ratio) by MoDMs pre‐incubated with 2‐DG (20 mm) for 15 min followed by 1 nmMCTR1, MCTR2, MCTR3, or Vehicle (0.05% EtOH) for 15 min. N = 4 human donors. (J) Efferocytosis of phrodo Red‐labeled ACs (3:1 ratio) by MoDMs pre‐incubated with aldolase inhibitor (10 µmAldometanib) for 1 h followed by 1 nm MCTR1, MCTR2, MCTR3, or Vehicle (0.05% EtOH) for 15 min. The Vehicle, MCTR1, MCTR2, and MCTR3 groups are shared with Figure 3D. N = 3 human donors. For (B), data are shown as mean log_2_ fold‐change and q‐values were calculated by two‐way ANOVA with multiple testing correction using the two‐stage Benjamini‐Krieger‐Yekutieli FDR procedure, where *q <0.05, **q <0.01. For (C‐J), data are shown as mean ± SD and p‐values were calculated using one‐way ANOVA (C,E) or two‐way ANOVA (F‐I) with Holm‐Sidak multiple comparison correction, where *p <0.05, **p <0.01, ***p <0.001 as indicated.

Since the phosphoproteomic screen identified a number of differentially phosphorylated glycolytic enzymes in MCTR‐treated macrophages (Figure 5A and **Figure** **6**A), we next investigated the effects of MCTRs on glycolytic metabolism. To that end, we profiled the steady‐state levels of various glycolytic and TCA cycle metabolites in MCTR‐treated human macrophages, in the absence of ACs. These experiments demonstrated that MCTR treatment increased levels of glucose‐6‐phosphate, fructose‐1,6‐bisphosphate, 3‐phospho‐D‐glycerate, and phosphoenolpyruvate — indicative of increased glycolysis (Figure 6B). On the other hand, levels of TCA cycle intermediates were not significantly affected (Figure 6B). Consistent with these observations, MCTR treatment also significantly increased levels of glucose‐6‐phosphate (Figure 6C) and phosphoenolpyruvate (Figure S6A, Supporting Information) in surgically injured planarians.

We further investigated MCTR‐mediated effects on macrophage glycolysis using Seahorse flux assays. These experiments revealed an increased glycolytic reserve and glycolytic capacity in MCTR‐treated macrophages (Figure 6D,E), while basal glycolysis was only marginally affected (Figure S6B, Supporting Information). Additionally, we found that MCTR treatment significantly increased glucose uptake by macrophages (Figure 6F), suggestive of increased glycolysis and consistent with the observed MCTR‐induced increases in steady‐state levels of glycolytic intermediates and glycolytic flux. Notably, this MCTR‐induced increase in glucose uptake was abrogated in Rac1 inhibitor‐treated macrophages (Figure 6F).

Rac1 has previously been shown to regulate glycolytic flux by enabling release of aldolase (a key glycolytic enzyme) from the cytoskeleton.^[^ ^30^ ^]^ To test the hypothesis that MCTRs enhance glycolysis by activating aldolase, we performed Seahorse flux assays using macrophages pre‐treated with Rac1 or aldolase inhibitors, which showed that both Rac1 and aldolase activity are required for MCTR‐mediated increases in macrophage glycolytic capacity (Figure 6G; Figure S6C, Supporting Information). On the other hand, blocking actin polymerization using cytochalasin D has previously been shown to increase both the release of aldolase from the cytoskeleton as well as its activity.^[^ ^30^ ^]^ Consistent with these observations, cytochalasin D pre‐treatment by itself significantly increased macrophage glycolytic capacity and prevented further MCTR‐mediated increases in this parameter (Figure 6G). Furthermore, reducing glycolytic flux using either the hexokinase inhibitor 2‐deoxyglucose (2‐DG) or an inhibitor of aldolase prevented MCTR‐mediated increases in both glucose uptake (Figure 6H) and efferocytosis (Figure 6J–l). Taken together, the above results point towards a Rac1 and aldolase‐dependent increase in glycolytic activity induced by MCTRs that primes macrophages for increased efferocytosis.

**Figure 7:**
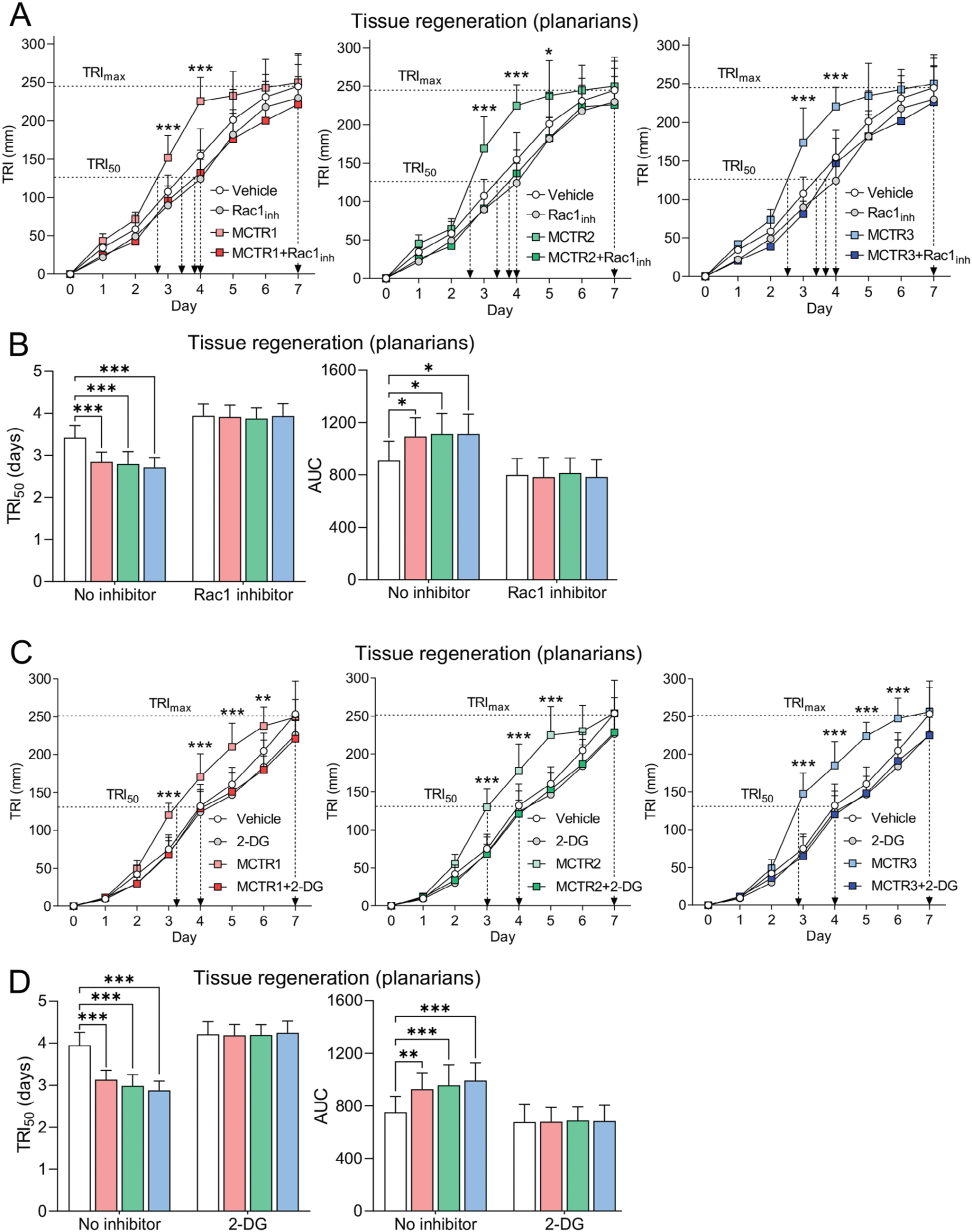
Enhancement of planarian tissue regeneration by MCTRs depends on Rac1 and glycolytic activity. A,B) Tissue regeneration in surgically injured *D. japonica* planarians incubated with Rac1 inhibitor (100 µm NSC23766) or DMSO control (Planaria water containing 0.1% DMSO), combined with 10 nm MCTR1, MCTR2, MCTR3, or Vehicle (Planaria water containing 0.05% EtOH). (A) Tissue regeneration indices (TRI) were determined over a 7‐day period, where TRI_max_ indicates the maximal tissue regeneration of the Vehicle control group at day 7, and TRI_50_ indicates the day at which 50% of TRI_max_ is reached for each group. The Vehicle and Rac1 inhibitor groups are shared between the three panels. (B) Half‐maximal TRI (TRI_50_) and Area‐under‐the‐curve (AUC) values were calculated from the tissue regeneration curves shown in A. N = 8 planarians per group. (C,D) Tissue regeneration in surgically injured *D. japonica* planarians incubated with 2‐DG (20 mM) or water control, combined with 10 nm MCTR1, MCTR2, MCTR3, or Vehicle (Planaria water containing 0.05% EtOH). (C) TRIs were determined over a 7‐day period, where TRI_max_ indicates the maximal tissue regeneration of the Vehicle control group at day 7, and TRI_50_ indicates the day at which 50% of TRI_max_ is reached for each group. The Vehicle and 2‐DG groups are shared between the three panels. (D) Half‐maximal TRI (TRI_50_) and Area‐under‐the‐curve (AUC) values were calculated from the tissue regeneration curves shown in C. N = 10 planarians per group. Data are shown as mean ± SD. p‐values were calculated using two‐way ANOVA with Holm‐Sidak multiple comparison correction. *p <0.05, **p <0.01, ***p <0.001 versus Vehicle control.

To test the physiological relevance of our findings and since AC clearance in planarians is linked with their ability to regenerate,^[^ ^21^ ^]^ we next assessed the ability of MCTRs to enhance tissue regeneration in surgically‐injured planarians treated with Rac1 or glycolytic inhibitors. Here we found that Rac1 and glycolytic activity were required for MCTR to enhance tissue regeneration, as indicated by AUC and TRI_50_ values that were unchanged between Vehicle control and MCTR groups in planarians treated with Rac1 inhibitor (**Figure** **7**A,B) or 2‐DG (Figure 7C,D). Together, these results establish a role for Rac1 and glycolysis modulation in the ability of MCTRs to regulate macrophage efferocytosis at sites of high AC burden in vivo.

## 3 Conclusion

Efferocytosis is a central pillar in both the maintenance of tissue homeostasis and its re‐establishment after inflammation and tissue injury.^[^ ^1^ ^]^ Clearance of multiple ACs by each individual macrophage through a process termed continual efferocytosis prevents the accumulation of ACs and initiates tissue repair.^[^ ^2^, ^7^ ^]^ Intriguingly, there is considerable heterogeneity in the ability of macrophages to preform efferocytosis, with multiple studies observing that only a small subset of macrophages are capable of effective AC clearance at any given time.^[^ ^9^, ^31^ ^]^ In line with this notion, we observed that only small proportions of macrophages express the initiating MCTR biosynthetic enzyme (human ALOX12 and mouse Alox12/15) when evaluating both monocyte‐derived human macrophages in vitro, as well as mouse resident macrophage populations in vivo (where there is likely a mix of embryonically derived and monocyte‐derived cells). This heterogeneity in ALOX12 expression was of functional relevance since it instructs nearby macrophages to take up ACs. Furthermore, macrophages deficient in Alox12/15 displayed a marked reduction in their ability to uptake ACs and perform continual efferocytosis. Intriguingly, we observed that this defect in the ability of ALOX12 (or Alox12/15) negative cells to perform continual efferocytosis was rectified when these cells were co‐cultured with either cells that expressed this enzyme or when incubated with MCTRs.

MCTRs enhanced efferocytosis in both ALOX12‐positive cells, which display an intrinsic ability to uptake ACs, as well as in ALOX12‐negative monocyte‐derived macrophages, which are less competent efferocytes. These findings suggest that the local production of MCTRs by efferocytosing macrophages acts a priming signal to instruct macrophages with a lower propensity to clear AC to initiate this vital process. Combined with the ability to enhance continual efferocytosis, MCTRs thereby increase the total efferocytic capacity of macrophages at sites of tissue injury. This local signaling mechanism can be activated when and where required, as suggested by the observation that MCTR production is rapidly but temporarily upregulated in both efferocytosing macrophages in vitro and in surgically‐injured planarians in vivo. Such localized effects were also observed in ALOX12‐overexpressing human macrophages (Figure 4A), and with co‐incubations of Control and Alox12/15‐deficient mouse macrophages (Figure 4B‐C). Furthermore, these studies describe efferocytosis‐boosting activities of MCTRs that are potentially complementary to efferocytosis‐modulating effects attributed to other Alox12/15‐derived products, such as oxidized phosphatidylethanolamine, which were shown to safeguard against auto‐immunity.^[^ ^32^ ^]^ Together, these results support the hypothesis that local MCTR production by efferocytosing macrophages primes these cells, as well as nearby non‐efferocytosing macrophages, for the clearance multiple rounds of ACs via continual efferocytosis.

Efferocytosis by macrophages is a tightly regulated multistep process that involves energy‐intensive mechanisms, including the modulation of cell membrane composition and the cytoskeleton.^[^ ^10^, ^15^ ^]^ Cellular metabolism plays a central role in efferocytosis, providing both the energy to sustain the engulfment of the AC as well as the pathways required to process the cellular cargo once ACs have been taken up.^[^ ^7^, ^8^, ^9^, ^10^, ^33^ ^]^ The cytoskeletal remodeling required for AC uptake is an energy‐intensive process dependent on a steady supply of cytoplasmic energy equivalents.^[^ ^10^ ^]^ Rac1 is a critical factor within this process, since it facilitates both the actin reorganization within the cell required for AC uptake, ^[^ ^26^ ^]^ as well as activation of glycolytic metabolism.^[^ ^30^ ^]^ Through unbiased phosphoproteomic profiling, we found that MCTRs regulate Rac1 activity and that the efferocytosis‐enhancing actions of these mediators are dependent on the activity of this Rho‐family GTPase. Furthermore, we observed that MCTRs regulated the phosphorylation status of several glycolytic enzymes and upregulated macrophage glycolysis. Published findings highlight a role for glycolysis in facilitating both initial AC uptake, ^[^ ^10^ ^]^ as well as continual efferocytosis via activation of PFKFB2.^[^ ^8^ ^]^ Our present findings identify MCTRs as key regulators of macrophage glycolytic activity via Rac1‐mediated activation of aldolase. Moreover, we found that inhibition of glycolysis reversed both the pro‐efferocytic activities of MCTRs, as well as the resultant enhancement of tissue regeneration. Given that PFKFB2 was not found to be differentially phosphorylated in our phosphoproteomic screen, these data suggest a mechanism of glycolytic activation by MCTRs during efferocytosis centered on Rac1 and aldolase that runs parallel to the previously described activation of PFKFB2.

The present study's strengths are that it evaluates the production and biological activities of MCTRs in a variety of tissue injury settings and organisms supporting a role for these molecules as evolutionary conserved fundamental regulators of continual efferocytosis. Furthermore, it provides a detailed interrogation of the molecular pathways activated by MCTRs in macrophages to exert their efferocytosis‐boosting effects, as the present findings support the hypothesis that AC degradation and the release of AC‐derived DNA is required for ALOX12 induction. Whilst DNase I treatment of ACs did not completely block ALOX12 induction, likely due to remnant DNA in the AC that was released upon AC uptake by the macrophage, TLR9 and AHR inhibition completely abrogated ALOX12 induction by ACs. There are also some limitations that should be considered when evaluating the present findings. First, the present study looks at the role of MCTRs in boosting efferocytosis exclusively within the context of sterile tissue injury. It remains to be established whether, and in what way, a pathogenic infection might affect the local production of MCTRs and their ability to upregulate efferocytosis. Second, we observed that macrophage ALOX12 expression and MCTR production is induced following AC uptake. Mechanistic evaluations on the contribution of AC to the biosynthesis of SPM during efferocytosis suggest that AC contribute SPM precursors, including essential fatty acids, which are converted to bioactive mediators by the macrophage.^[^ ^34^ ^]^ Whether AC contribute precursors in the production of MCTRs remains to be established in future studies. Finally, it is well appreciated that resolution of inflammation and clearance of ACs via efferocytosis is critical for successful tissue repair. However, the factors involved in priming parenchymal cells for proliferation and differentiation to ultimately regain functional tissue, and whether efferocytosing macrophages produce such factors, remain to be established.

In summary, the present findings indicate that at ALOX12 (or Alox12/15 in mice) expressing macrophages rapidly upregulate MCTR production during efferocytosis that in an autocrine and paracrine manner facilitate continual efferocytosis promoting cytoskeletal remodeling and a shift in cellular metabolism. At a molecular level, this priming occurred via the modulation of Rac1 activity and the upregulation of glycolysis, resulting in enhanced tissue repair responses. Taken together, these findings elucidate a previously unappreciated mechanism that coordinates continual efferocytosis by macrophages at sites of high ACs burden to facilitate tissue repair and the restitution of organ function.

## 4 Experimental Section

### Animal Experiments

*Cx3cr1*:cre‐driven Alox12/15‐deficient (Mac*^Alox12/15^* ^KO^) mice were generated by crossing loxP‐flanked (floxed) Alox12/15 mice (B6.Cg‐Alox15tm1.1Nadl/J; strain #03 1835; The Jackson Laboratory) with mice expressing Cre‐recombinase from the *Cx3cr1* gene promoter (B6J.B6N(Cg)‐Cx3cr1tm1.1(cre)Jung/J; strain #02 5524; The Jackson Laboratory). Mouse experiments were approved by the United Kingdom's Home Office (P998AB295). All animal experimental procedures were performed while adhering to the UK's Guidance on the Operation of Animals, Scientific Procedures Act (1986), and Laboratory Animal Science Association Guidelines’ Guiding Principles on Good Practice for Animal Welfare and Ethical Review Bodies. Mice were kept on a 12 h light/dark cycle (07:00h‐19:00 h) and provided with a standard laboratory diet and water ad libitum. Planarian flatworms (Dugesia japonica) were maintained at 19 °C in sterile Planaria water (ddH_2_O containing 0.2 g L^−1^ InstantOcean Sea Salt) and fed pureed calf liver once per week. In all experiments, animals were randomly assigned to control and treatment groups.

### Dexamethasone‐Induced Thymus Apoptosis Mouse Model

For lipid mediator profiling and flow cytometric analysis of dexamethasone‐induced thymus apoptosis, 10‐week‐old male and female C56BL/6J mice (strain #000664; The Jackson Laboratory) were injected intraperitoneally with 250 µL PBS containing either 250 µg dexamethasone, or vehicle (0.63% dimethyl sulfoxide). Mice were euthanized 18 h later by CO_2_ asphyxiation and thymi were harvested, cut into two lobes, mechanically disaggregated, and processed for either flow cytometric analysis of macrophage efferocytosis, or measurement of endogenous lipid mediator levels by LC‐MS/MS as described below.

For immunofluorescence microscopy analysis of dexamethasone‐induced thymus apoptosis, 15‐week‐old male Mac*^Alox12/15^* ^KO^ or Alox12/15‐floxed littermates were injected intraperitoneally with 250 µL PBS containing either 250 µg dexamethasone, or vehicle (0.63% dimethyl sulfoxide). Mice were euthanized 18 h later by CO_2_ asphyxiation and thymi were harvested. One lobe of each thymus was fixed in 10% formalin for 6 h, followed by paraffin embedding and sectioning in 5 µm sections. Sections were stained for ACs using the ApopTag Red In Situ Apoptosis Detection Kit (Millipore) according to manufacturer's instructions, followed by staining with fluorescently‐conjugated antibodies against mouse F4/80 (FITC; Biolegend) and Alox12/15 (AF647; Bioss). Stained samples were mounted on glass microscopy slides with MOWIOL and images were acquired using a Celldiscoverer 7 high‐content imaging system (Zeiss). Data were analyzed using the ZEN 2.6 software (Zeiss).

### In Vivo Peritoneal Efferocytosis Mouse Model

Apoptotic target cells for efferocytosis were generated as follows: human promyelocytic HL‐60 cells were seeded at 1.5 × 10^6^ cells mL^−1^ in RPMI‐1640 medium containing 10% fetal bovine serum and 1% penicillin/streptomycin in 35‐mm plates, irradiated with UV‐C light (254 nm) for 15 min, and incubated at 37 °C and 5% CO_2_ for 2 h. Apoptosis induction was verified by flow cytometry using the APC Annexin V Apoptosis Detection Kit with PI (Biolegend), where Annexin V‐positive and Annexin V/PI‐double positive cells were considered apoptotic. Apoptotic HL‐60 cells were then washed with PBS and labeled with 1 µm pHrodo Red Succinimidyl Ester (Invitrogen) or 1 µm CypHer5E NHS Ester (Sigma) in PBS for 30 min at room temperature, followed by washing twice with PBS. For in vivo peritoneal macrophage continual efferocytosis assays, 15‐week old male Mac*^Alox12/15^* ^KO^ or Alox12/15‐floxed littermates were injected intraperitoneally with 100 µL PBS containing 3.33 × 10^6^ unlabeled apoptotic HL‐60 cells. After 2 h, 100 µL PBS containing either 10 ng MCTR3 or vehicle (0.01% v/v EtOH) and 100 µL PBS containing 3.33 × 10^6^ pHrodo Red‐labeled apoptotic HL‐60 cells were sequentially injected peritoneally. After 45 min, mice were euthanized by CO_2_ asphyxiation, peritoneal lavages were collected by flushing the peritoneal cavity with 5 mL PBS containing 2 mm EDTA, and lavages were analyzed by flow cytometry as described below.

### AC Quantification by TUNEL Assays in Planarian Flatworms

Planarian flatworms (Dugesia japonica) were starved 7 days and subjected to post‐ocular head resection. Posterior portions were placed in Planaria water containing MCTR1, MCTR2, or MCTR3 (0.1‐10 nm) or vehicle (0.05% v/v EtOH) for 4 h. In experiments with Rac1 inhibition, posterior portions were placed in Planaria water for 3 h after which Rac1 inhibitor NSC23766 (100 µm) or equal volume DMSO was added to each well for 15 min, followed by MCTR1, MCTR2, or MCTR3 (1 nm) for 45 min. After incubations, Planarian flatworms were transferred into 2% HCl in ddH_2_O for 5 min to euthanize animals, followed by 4% paraformaldehyde for 20 min to fix specimens. After washing twice with PBS containing 0.3% Triton X‐100 (PBS+TX‐100), specimens were bleached overnight in 6% H_2_O_2_ in PBS+TX‐100. Fixed and bleached Planaria were stained for ACs using the ApopTag Red In Situ Apoptosis Detection Kit (Millipore) according to previously published protocols.^[^ ^35^ ^]^ Stained samples were mounted on glass microscopy slides with MOWIOL and the number of TUNEL+ cells were determined using the Celldiscoverer 7 high‐content imaging system (Zeiss). Data were analyzed using the ZEN 2.6 software (Zeiss).

### Tissue Regeneration Assays in Planarian Flatworms

Planarian flatworms (Dugesia japonica) were starved for 7 days prior to experiments. Tissue regeneration was assessed as previously described.^[^ ^13^ ^]^ Briefly, Planarians were subjected to post‐ocular head resection and posterior portions were subsequently placed in Planaria water containing MCTR1, MCTR2, or MCTR3 (0.1‐10 nm) or vehicle (0.05% v/v EtOH) and imaged over a 7‐day period at 24 h intervals to assess tissue regeneration. Images were analysed using ImageJ software. A tissue regeneration index (TRI) was calculated by dividing the size of the total regenerated tissue area (A) by the post‐ocular width (W) of the animal (TRI = A/W).

### Primary Human Peripheral Blood Mononuclear Cell (PBMC) Isolation and Monocyte‐Derived Macrophage (MoDM) culture

Human peripheral blood mononuclear cells (PBMCs) were isolated from healthy human volunteer blood sourced from the NHS Blood and Transplant bank using Histopaque‐1077 (Sigma) and density centrifugation following the manufacturer's instructions. PBMCs were subsequently resuspended in PBS at 3 × 10^6^ cells mL^−1^ and 30 × 10^6^ cells were seeded in 10‐cm petri dishes. Cells were allowed to adhere by incubating at 37 °C for 45 min. Non‐adherent cells were washed away using PBS and the remaining adherent cells were differentiated to MoDMs by incubating in RPMI‐1640 medium containing 10% human serum, 1% penicillin/streptomycin, and 20 ng mL^−1^ recombinant human GM‐CSF for 7 days at 37 °C and 5% CO_2_, with media being refreshed on day 3. After 7 days, cells were lifted from 10‐cm petri dishes by incubating cells in PBS without calcium or magnesium plus 5 mm EDTA for 30 min followed by gentle pipetting.

### Primary Mouse Bone Marrow‐Derived Macrophage (BMDM) Culture

Bone marrow was isolated from mononuclear phagocyte‐specific Mac*^Alox12/15^* ^KO^ or Alox12/15‐floxed littermates and differentiated to bone marrow‐derived macrophages according to established protocols.^[^ ^36^ ^]^ Briefly, tibias and femurs were flushed with PBS without calcium or magnesium and cells were passed through a 70 µm strainer. Cells were then enumerated, resuspended to 4 × 10^5^ cells mL^−1^ in DMEM‐HG medium containing 10% human serum, 1% penicillin/streptomycin, and 10 ng mL^−1^ recombinant mouse M‐CSF, and 8 mL cells were seeded in 10‐cm petri dishes. Cells were then incubated for 7 days at 37 °C and 5% CO_2_, with 5 mL fresh media being added on day 3. After 7 days, cells were lifted from 10‐cm petri dishes by incubating cells in PBS without calcium or magnesium plus 5 mm EDTA for 30 min followed by gentle pipetting.

### In Vitro Macrophage Efferocytosis and Bead Phagocytosis Assays

For single‐round efferocytosis assays, human MoDM or mouse BMDM were seeded in 96‐well plates at 4 × 10^4^ cells well^−1^, followed by serum‐starvation (0% human serum for MoDM, 1% fetal bovine serum for BMDM) for 16 h. For efferocytosis assays with human MoDM overexpressing GFP‐tagged ALOX12, MoDM were transfected with 300 ng pCMV6‐hALOX12‐GFP (Origene RG210211) or pCMV6‐AC‐GFP backbone control (Origene PS100010) vectors for 48 h using Lipofectamine LTX with PLUS reagent (Invitrogen) according to manufacturer's instructions, followed by staining with Hoechst 33 342 ReadyFlow (Invitrogen) for 15 min. For mouse Mac*^Alox12/15^* ^KO^ and Control BMDM co‐culture efferocytosis assays, cells were labeled with CellBrite Green (Biotium) or PKH26 (Sigma) in suspension according to manufacturer's instructions, followed by seeding in 96‐well plates at various ratios as indicated in the figure panels to a total cell count of 0.4 × 10^5^ cells well^−1^. In select experiments, macrophages were pre‐treated with inhibitors as described in the figure legends, followed by MCTR1, MCTR2, or MCTR3 (1 nm), or all three MCTRs combined (0.33 nm of each MCTR for a total of 1 nm), or vehicle (0.05% v/v EtOH) for 15 min at 37 °C and 5% CO_2_. Apoptotic pHrodo Red‐labeled HL‐60 cells were then added to the macrophages at indicated ratios of AC:macrophage and the increase in pHrodo Red signal over time (representing AC uptake by macrophages) was quantified over the course of 3 h using a ZEISS Celldiscoverer 7 high‐content imaging system with appropriate filters set at 37 °C and 5% CO_2_. Data were analyzed using the ZEN 2.6 software (Zeiss).

### In Vitro Macrophage Continual Efferocytosis Assays

For continual efferocytosis assays, human MoDM or mouse BMDM were seeded in 96‐well plates at 4 × 10^4^ cells well^−1^, followed by serum‐starvation (0% human serum for MoDM, 1% fetal bovine serum for BMDM) for 16 h. Macrophages were stained with CellBrite Green (Biotium) for 1 h at 37 °C, washed thrice with RPMI‐1640 medium, and pre‐treated with MCTR1, MCTR2, or MCTR3 (1 nm) or vehicle (0.05% v/v EtOH) for 15 min at 37 °C and 5% CO_2_ (referred to as “Pre‐treatment” in the figures). Apoptotic pHrodo Red‐labeled HL‐60 cells were then added to the macrophages at 3:1 ratio of AC:macrophage (first round of ACs). After 1 h incubation at 37 °C and 5% CO_2_, unbound ACs were removed by washing thrice with RPMI‐1640 medium and macrophages were left to rest for 2 h at 37 °C and 5% CO_2_ in RPMI‐1640 medium. For select experiments, macrophages were treated with MCTR1, MCTR2, or MCTR3 (1 nm) or vehicle (0.05% v/v EtOH) for the last 15 min of the 2 h rest period (“Interjacent treatment”). Apoptotic CypHer5E‐labeled HL‐60 cells were then added to the macrophages at 3:1 ratio of AC:macrophage (second round of ACs). The increase in pHrodo Red and CypHer5E signal over time (representing first and second round AC uptake by macrophages) was quantified over the course of 3 h using a ZEISS Celldiscoverer 7 high‐content imaging system with appropriate filters (CellBrite Green = GFP filter set; pHrodo Red = Cy3 filter set; CypHer5E = Cy5 filter set) set at 37°C and 5% CO_2_. Data were analyzed using the ZEN 2.6 software (Zeiss). Continual efferocytosis was defined as pHrodo Red^+^ macrophages that became CypHer5E^+^ over the course of the 3 h incubation.

### Macrophage Rac1/RhoA Activity G‐LISA Assays

Human MoDM were seeded in 24‐well plates at 1 × 10^5^ cells well^−1^ and serum‐starved (0% human serum) for 24 h. MoDM were incubated with MCTR1, MCTR2, or MCTR3 (10 nm) or vehicle (0.05% v/v EtOH) for indicated times and active Rac1 or RhoA was measured using the Rac1 or RhoA G‐LISA Activation Assay kit (Cytoskeleton) according to the manufacturer's instructions. Background control values from cells pre‐treated for 24 h with Rac1 inhibitor NSC23766 (100 µm) or Rho inhibitor I CT‐04 (0.5 µg mL^−1^) were subtracted from sample values to get Rac1 or RhoA activity values.

### Targeted Determination of *Maresin* and *Maresin Conjugates in Tissue Regeneration* Abundance by LC‐MS/MS (Lipid Mediator Profiling)

Mouse thymi (one half lobe), surgically‐injured planarians (30 worms per replicate), human macrophages incubated with ACs for 1, 3, 24, or 48 h (1 × 10^6^ cells; washed thrice with PBS to remove unbound ACs), or human macrophages pre‐treated with or without 5 µm AhR inhibitor CH‐223191 followed by incubation with ACs for 24 h (2.4 × 10^6^ cells; washed thrice with PBS to remove unbound ACs) were collected in ice‐cold LC‐MS/MS‐grade methanol containing deuterium‐labeled internal standards (500 pg of each standard per sample) covering the chromatographic regions used for *maresin* and *maresin conjugates in tissue regeneration* identification, namely: d_5_‐MaR1, d_5_‐LTC_4_, d_5_‐LTD_4_, and d_5_‐LTE_4_ (Cayman Chemicals). After addition of ice‐cold methanol containing deuterium‐labeled internal standards, thymus and planarian samples were homogenized using a glass dounce or metallic bead homogenizer. Samples were then kept at −20 °C for at least 45 min and centrifuged 5 min at 1000 x *g* to precipitate proteins. Mediators were extracted from the resulting supernatants using an ExtraHera System (Biotage) with solid‐phase Isolute C18 500 mg/6 mL columns (Biotage). Mediators were eluted from the C18 columns using methyl formate (for MaR1 and MaR2) and methanol (for MCTRs). Eluates were brought to dryness by incubating at 37°C under a constant stream of nitrogen and samples were subsequently resuspended in methanol/water (1:1 v/v) for injection on a Shimadzu LC‐20AD HPLC and a Shimadzu SIL‐20AC autoinjector, paired with a QTrap 5500 or QTrap 6500+. For the chromatographic separation of mediators eluted in methyl formate fraction, an Agilent Poroshell 120 EC‐C18 column (100 mm × 4.6 mm × 2.7 µm) was kept at 50 °C and mediators eluted using a mobile phase consisting of methanol/water/acetic acid of 20:80:0.01 (vol/vol/vol), which was ramped to 50:50:0.01 (vol/vol/vol) over 0.5 min, then to 80:20:0.01 (vol/vol/vol) from 2 min to 11 min, maintained till 14.5 min, and then rapidly ramped to 98:2:0.01 (vol/vol/vol) for the next 0.1 min. This was subsequently maintained at 98:2:0.01 (vol/vol/vol) for 5.4 min, and the flow rate was kept at 0.5 mL min^−1^. The QTrap was operated in negative ionization mode using a multiple reaction monitoring method. For the chromatographic separation of mediators eluted in the methanol fraction, an Agilent Poroshell 120 EC‐C18 column (100 mm × 4.6 mm × 2.7 µm) was kept at 50 °C and mediators eluted using a mobile phase consisting of methanol/water/acetic acid 55:45:0.5 (vol/vol/vol) over 5 min, which was ramped to 80:20:0.5 (vol/vol/vol) for 2 min, maintained at 80:20:0.5 (vol/vol/vol) for the next 3 min, ramped to 98:2:0.5 (vol/vol/vol) over 3 min and kept for 3 min. The QTrap was operated in positive ionization mode using a multiple reaction monitoring method. Data were analyzed using SCIEX OS 2.1. For peak integration and identification of each mediator, the following established criteria were used: 1) a clearly defined peak with a retention time that matches the corresponding synthetic or authentic standard, 2) the signal‐to‐noise ratio of the integrated peak is ≥5, and 3) a secondary transition for the same mediator contains a peak at the correct retention time with a signal‐to‐noise ratio ≥3 and/or an MS/MS spectrum with a score ≥70% match against the reference MS/MS spectrum for the mediator of interest using the Library Search Smart Confirmation function in SCIEX OS 2.1. The following transitions Q1>Q3 were employed for the identification of MaRs and MCTRs: MCTR1: 650>205, 650>191, 650>173, 650>215; MCTR2: 521>205, 521>191, 521>173; MCTR3: 464>191, 464>173, 464>205; MaR1: 359>250, 359>221; MaR2: 359>221, 359>191. Calibration curves were obtained for each mediator using synthetic compound mixtures that gave linear calibration curves with r^2^ values of 0.98–0.99.

### Determination of Intracellular Metabolite Abundance by LC‐MS/MS (Metabolomics)

Human MoDM were seeded in 24‐well plates at 1 × 10^5^ cells/well and incubated with MCTR1, MCTR2, or MCTR3 (0.1 or 1 nm) for 1 h. Planarian flatworms (Dugesia japonica) were starved 7 days and subjected to post‐ocular head resection, after which posterior portions were placed in Planaria water containing MCTR1, MCTR2, or MCTR3 (10 nm) or vehicle (0.05% v/v EtOH) for 3 h. MoDM were harvested by adding 250 µL ice‐cold methanol containing ^13^C‐labeled internal standards for glycolysis and TCA cycle intermediates (10 µg ^13^C_6_‐Glucose, 5 µg ^13^C_2_‐citric acid, 5 µg ^13^C_2_‐fumaric acid per sample; Sigma), followed by 250 µL ice‐cold ultrapure water. Each well was then rinsed with an additional 250 µL ice‐cold methanol with ^13^C‐labeled internal standards followed by 250 µL ice‐cold ultrapure water. Planarian flatworm sections were directly placed in 1 mL ice‐cold methanol containing ^13^C‐labeled internal standards for glycolysis and TCA cycle intermediates (10 µg ^13^C_6_‐Glucose, 5 µg ^13^C_2_‐citric acid, 5 µg ^13^C_2_‐fumaric acid per sample; Sigma), followed by homogenization using a glass dounce and addition of 1 mL ice‐cold ultrapure water. For all samples, 1 volume of chloroform was added to the entire homogenate, followed by thorough mixing and centrifugation for 10 min at 4000 x *g* at 4 °C. The polar top layer was collected and brought to dryness by incubating at 37 °C under a constant stream of nitrogen. Samples were resuspended in ddH_2_O and analyzed using a Qtrap 6500+ (SCIEX) coupled to a Shimadzu SIL‐20AC autoinjector and LC‐20AD binary pump as previously described.^[^ ^37^ ^]^ A Synergi Hydro‐RP column (250 × 4.6 mm × 4 µm, Phenomenex) column maintained at 30 °C was used with a gradient of methanol/water/acetic acid of 0:100:0.5 (vol:vol:vol), which was ramped to 100:0:0.5 (vol:vol:vol) over 16 min. The flow rate was maintained at 0.5 mL min^−1^. A previously described multiple reaction monitoring (MRM) method was used to identify target metabolites.^[^ ^37^ ^]^ Data were analyzed using SCIEX OS 2.1.

### Phosphopeptide Quantification by LC‐MS/MS (Phosphoproteomics)

Phosphoproteomics experiments were carried out as described previously.^[^ ^38^ ^]^ Briefly, human MoDM were incubated with MCTR1, MCTR2, or MCTR3 (1 nm) for 5 or 15 min and lysed in 8 m urea supplemented with phosphatase inhibitors (10 mm Na_3_VO_4_, 100 mm β‐glycerol phosphate, 25 mm Na_2_H_2_P_2_O_7_ [Sigma]). After trypsin digestion, phosphopeptides were enriched from total peptides by TiO_2_ chromatography as previously described.^[^ ^24^ ^]^ Dried phosphopeptides were dissolved in 0.1% trifluoroacetic acid and analyzed using an UltiMate 3000 RSLCnano coupled on‐line to a Q‐Exactive Plus mass spectrometer (Thermo Fisher Scientific). Elution was performed using a gradient of 3% to 35% buffer B (0.1% formic acid in acetonitrile) over 120 min at a flow rate of 300 nL min^−1^ with buffer A (0.1% formic acid in water) being used to balance the mobile phase. Spray voltage was set to 1.95 kV and capillary temperature was set to 255°C. The Q‐Exactive Plus was operated in data‐dependent mode with one survey MS scan followed by 15 MS/MS scans. The full scans were acquired in the mass analyser at 375–1500 m/z with a resolution of 70.000, and MS/MS scans were obtained with a resolution of 17.500. Raw data files were converted into Mascot Generic Format using Mascot Distiller (version 2.5.1) and evaluated against human entries in the SwissProt database (release 12/2015) using the Mascot search daemon (version 2.5.0). The allowed parent mass‐to‐charge value window was set to 10 ppm, and the allowed fragment mass window was set to 25 mmu. Modifications included in the searches were phosphorylation of serine, threonine, and tyrosine, as well as methionine oxidation and pyroglutamate formation. Differentially phosphorylated peptide hits from the 5 min and 15 min mctr treatment datasets were combined by pooling all hits for each MCTR, and pathway and protein‐protein hub analyses were performed using Enrichr.^[^ ^39^ ^]^

### Seahorse XF Extracellular Acidification Rate (ECAR) Assays

Extracellular acidification rates (ECAR) as a measure of glycolytic activity were determined using the Seahorse XF96 Analyzer (Agilent) according to manufacturer's instructions. Human MoDMs were seeded at 5 × 10^4^ cells well^−1^ in XFe96‐well plates and left to attach overnight, followed by pre‐treatment with inhibitors and MCTR1, MCTR2, MCTR3, or vehicle control as described in the figure legends. Wells without cells were included as a background control. Sensor plates were calibrated overnight in a CO_2_‐free incubator at 37 °C using 200 µL well^−1^ XF Calibrant Solution (Agilent). Immediately before assay start, culture medium was replaced with glucose‐free RPMI‐1640 medium supplemented with 2 mM L‐glutamine and cells were incubated 1 h in a CO_2_‐free incubator at 37 °C. Injection ports were loaded with 10x injection mixes to obtain a final concentration in each well of 10 mm glucose after the first injection, 2 µm oligomycin after the second injection, and 20 mm 2‐deoxyglucose after the third injection. After the run, supernatants were aspirated, protein was isolated using RIPA Lysis and Extraction Buffer (Thermo Fisher), and protein content of the lysates was determined using Precision Red Advanced Protein Assay (Cytoskeleton, Inc.). ECAR values were normalized to total protein content of each well. Parameters of glycolytic activity (glycolysis, glycolytic reserve, glycolytic capacity) were calculated from ECAR values according to manufacturer's instructions.

### Glucose Uptake Assays

Human MoDMs were seeded at 4 × 10^4^ cells well^−1^ in 96‐well plates and left to attach overnight at 37 °C and 5% CO_2_. Prior to glucose uptake assays, cells were pre‐treated with inhibitors as described in the figure legends and glucose starved in glucose‐free RPMI‐1640 medium (Agilent) supplemented with 2 mM L‐glutamine (Sigma) for 6 h. Then, cells were treated with 1 nm MCTR1, MCTR2, MCTR3, or vehicle control for 15 min followed by incubation with 25 µg mL^−1^ 2‐Deoxy‐2‐[(7‐nitro‐2,1,3‐benzoxadiazol‐4‐yl)amino]‐D‐glucose (2‐NBDG; Sigma) for 30 min. Cells were washed thrice with PBS to remove free 2‐NBDG and fluorescence was immediately determined in‐plate using a NOVOstar plate reader (BMG Labtech) with FITC filter set.

### RNA Extraction, Reverse Transcription PCR, and Quantitative PCR

For mRNA expression analysis, human MoDM were seeded in 12‐well culture plates at 4 × 10^5^ cells well^−1^, serum‐starved (0% human serum) for 16 h, incubated with MCTR1, MCTR2, or MCTR3 (10 nm) or vehicle (0.05% v/v EtOH) for 15 min, then loaded with apoptotic HL‐60 cells at a 3:1 ratio of AC:macrophage for 1, 3, or 24 h, after which unbound ACs were washed away. Total RNA was extracted using the RNeasy Mini kit (Qiagen) according to manufacturer's instructions. RNA purity and concentration were assessed using a NanoDrop 8000 spectrophotometer (ThermoFisher). Total RNA (1 µg) was reverse‐transcribed using the iScript cDNA Synthesis Kit (Biorad) according to manufacturer's instructions and resulting cDNA was diluted tenfold in ddH_2_O. Quantitative PCR reactions were performed on a QuantStudio 7 Flex System (Applied Biosystems) using QuantiFAST SYBR Green (Qiagen). Quantitect Primer assays (Qiagen) for the indicated genes were used, with RPL13A and RPLP0 Quantitect Primer assays included as housekeeping control genes. Quantification cycles (C_q_), primer amplification efficiency, and transcript starting concentration (N_0_) were calculated using LinRegPCR software.^[^ ^40^ ^]^

### Flow Cytometric Analysis of ALOX12 Expression

For analysis of ALOX12 protein expression in apoptotic HL‐60 cells and human MoDM, rabbit polyclonal anti‐ALOX12 antibody (Abgent AP8877B) was conjugated with Alexa Fluor (AF)594 fluorophore using Lightning‐Link conjugation kit (Abcam) according to manufacturer's instructions. Apoptotic HL‐60 cells were prepared as described above. For experiments involving bead phagocytosis, 800000 4 µm FluoSphere Y/G polystyrene beads (ThermoFisher) or 5 µm streptavidin beads (Bangs Laboratories) were used per 400000 macrophages (2:1 bead:macrophage ratio). Streptavidin beads (100 µL 1% w/v) were coated with 3 µg biotinylated phosphatidylserine (Echelon Biosciences) for 30 min at room temperature. Free biotinylated phosphatidylserine was washed away with PBS and unbound streptavidin was blocked with 12.5 mm EZ‐link Amine‐PEG3‐biotin (ThermoFisher) for 10 min at room temperature. Coated and blocked beads were then labeled with amine‐reactive CypHer5E NHS ester (Sigma) for 30 min at room temperature and washed with PBS.

MoDM were seeded in 12‐well plates at 4 × 10^5^ cells/well and loaded with CypHer5E‐labeled apoptotic HL‐60 cells for 1, 3, or 24 h, or 4 µm FluoSphere Y/G polystyrene beads for 24 h, or 5 µm CypHer5E‐labeled streptavidin beads with or without phosphatidylserine coating for 24 h. In select experiments, MoDM were pre‐treated with 1 µg mL^−1^ TLR9 inhibitor ODN INH‐18 (Invivogen) or 5 µm Aryl hydrocarbon receptor (AhR) inhibitor CH‐223191 (Sigma) for 1 h prior to addition of CypHer5E‐labeled apoptotic HL‐60 cells for 24 h, or apoptotic HL‐60 cells were pre‐treated with 100 UI mL^−1^ DNAse I (Roche) for 1 h at 20 °C followed by washing with PBS containing 0.02% (w/v) bovine serum albumin to remove membrane‐associated DNA complexes. After incubations, MoDM were washed thrice with PBS and lifted from culture plates by incubating in PBS without calcium or magnesium plus 5 mm EDTA for 30 min followed by gentle pipetting. MoDM or apoptotic HL‐60 cells were then permeabilized and fixed using the Foxp3 / Transcription Factor Staining Buffer Set (eBioscience) according to manufacturer's instructions and permeabilized cells were stained with fluorophore‐conjugated antibodies against ALOX12 (AF594) or non‐specific rabbit IgG isotype control for 30 min at 4 °C.

For analysis of mouse peritoneal macrophage efferocytosis, peritoneal lavages were collected by flushing the peritoneal cavity with 5 mL PBS containing 2 mM EDTA, cells were centrifuged 5 min at 500 x *g*, and cell pellets were incubated with mouse TruStain FcX (Biolegend) for 30 min on ice. Cells were then stained with fluorescently‐conjugated antibodies against either: CD3 (APC/Cy7), CD19 (APC/Cy7), SiglecF (APC/Cy7), Ly6G (APC/Cy7), CD11b (PE/Cy5), F4/80 (BV421), CD102 (FITC) (all from Biolegend), followed by fixation and permeabilization using the Foxp3 / Transcription Factor Staining Buffer Set (eBioscience), and intracellular staining of Alox12/15 (AF647; Bioss) for continual efferocytosis assays with pHrodo Red‐labeled ACs in small versus large peritoneal macrophages.

For analysis of mouse thymus macrophage efferocytosis, disaggregated thymus cell suspensions were stained with fluorescently‐conjugated antibody against mouse F4/80 (BV421, Biolegend), permeabilized and fixed with the Foxp3 / Transcription Factor Staining Buffer Set (eBioscience) according to manufacturer's instructions, and then stained for intracellular ACs using Annexin V‐APC (Biolegend).

For all experiments, separate cells stained with corresponding fluorescently‐labeled isotype control antibodies were included as background control, and fluorescence intensity in samples and control stainings (isotype‐labeled, unstained, and Ultracomp eBeads with single fluorophores for compensation calculation) was evaluated on a BD LSRFortessa flow cytometer. Data were analyzed using FlowJo v10.3.

### Statistical Testing

Statistical testing was performed using GraphPad Prism 9. Results are presented as mean ± SD unless otherwise indicated in the figure legends. For all experiments, the number of biological replicates (N) and the statistical tests used in each figure panel are described in the figure legends, where a p‐ or q‐value <0.05 was considered significant. For animal experiments, group sizes were determined based on the variability observed in prior or pilot experiments. Normal distribution of data was tested using the Shapiro‐Wilk normality test. For data following a normal distribution, statistically significant differences were tested using two‐tailed Student's T‐test, one‐way ANOVA, or two‐ANOVA with multiple testing correction using the Holm‐Sidak post‐hoc multiple comparisons correction or a two‐stage Benjamini‐Krieger‐Yekutieli false‐discovery rate (FDR) procedure as indicated in the figure legends. For data not following a normal distribution or for experiments where group sizes were smaller than 6, the non‐parametric Kruskal‐Wallis test with Dunn's post hoc multiple comparisons correction was used.

## Conflict of Interest

J.D. is an inventor on patents related to the composition of matter and/or use of pro‐resolving mediators, including MCTRs, some of which are licensed by Brigham and Women's Hospital or Queen Mary University of London for clinical development

## Author Contributions

J.D. conceived the overall research plan. J.D. and D.S.K. designed the experiments. D.S.K., R.D.M., and V.R. conducted the experiments and interpreted data. D.S.K. and J.D. interpreted data; P.C. and J.D. supervised experiments. All authors contributed to manuscript preparation.

## Supporting information

Supporting Information

## Data Availability

The data that support the findings of this study are openly available in BioStudies at https://www.ebi.ac.uk/biostudies, reference number S‐BSST943.
