# Supplementary material for: Efferocyte‐Derived MCTRs Metabolically Prime Macrophages for Continual Efferocytosis via Rac1‐Mediated Activation of Glycolysis
Source: Adv Sci (Weinh). 2023 Dec 8;11(7):2304690. doi: 10.1002/advs.202304690 (PMC10870015; doi:10.1002/advs.202304690)
Supplement: Supplementary file 1 — Supporting Information [file ADVS-11-2304690-s001.pdf]

ADVANCED SCIENCE
Open Access

Supporting Information

for *Adv. Sci.*, DOI 10.1002/advs.202304690

Efferocyte-Derived MCTRs Metabolically Prime Macrophages for Continual Efferocytosis via Rac1-Mediated Activation of Glycolysis

*Duco Steven Koenis, Roberta de Matteis, Vinothini Rajeeve, Pedro Cutillas and Jesmond Dalli**

Supporting Information

Efferocyte-derived MCTRs metabolically prime macrophages for continual efferocytosis via Rac1-mediated activation of glycolysis

*Duco S. Koenis, Roberta de Matteis, Vinothini Rajeeve, Pedro Cutillas, Jesmond Dalli**

Contents:

Figure S1; related to Figure 1.

Figure S2; related to Figure 1-2.

Figure S3; related to Figure 1-2.

Figure S4; related to Figure 4.

Figure S5; related to Figure 5.

Figure S6; related to Figure 6.

Table S1: Differentially-phosphorylated peptides from phosphoproteomics analysis of human monocyte-derived macrophages treated with MCTR1, MCTR2, MCTR3, or vehicle.

Figure S1

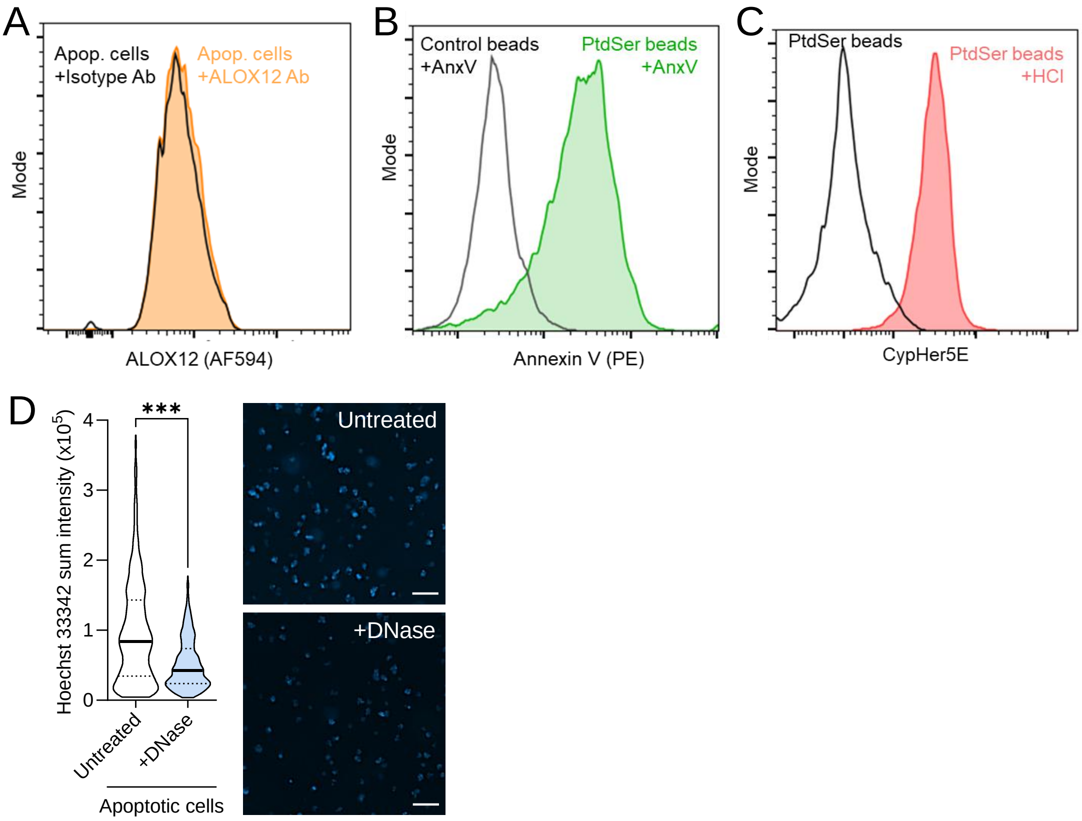

Figure S1; related to Figure 1.
(A) Histogram plot for ALOX12 median fluorescence intensity (MFI) in apoptotic HL-60 cells labelled with Alexa Fluor 594-conjugated anti-ALOX12 or rabbit IgG isotype antibody and acquisition by flow cytometry.
(B) Histogram plot for Annexin V median fluorescence intensity (MFI) in Control beads or beads coated with phosphatidylserine (PtdSer beads) followed by labelling with PE-conjugated Annexin V and acquisition by flow cytometry.
(C) Histogram plot for CypHer5E median fluorescence intensity (MFI) in PtdSer beads conjugated with CypHer5E followed by incubation with or without 2 mM HCl and acquisition by flow cytometry.
(D) Quantification of AC-associated DNA by fluorescence microscopy in untreated ACs or ACs treated with 10µg/ml DNase I for 1 hr and stained with Hoechst 33342. Violin plots are based on 1774 cells (Untreated group) or 1407 cells (+DNase group) and horizontal lines inside violin plots indicate median (solid) and quartiles (dashed); *** $p < 0.001$ by Mann-Whitney U-test. Micrograph insets show Hoechst 33342 signal in ACs from indicated groups; scale bar = 50µm.

Figure S2

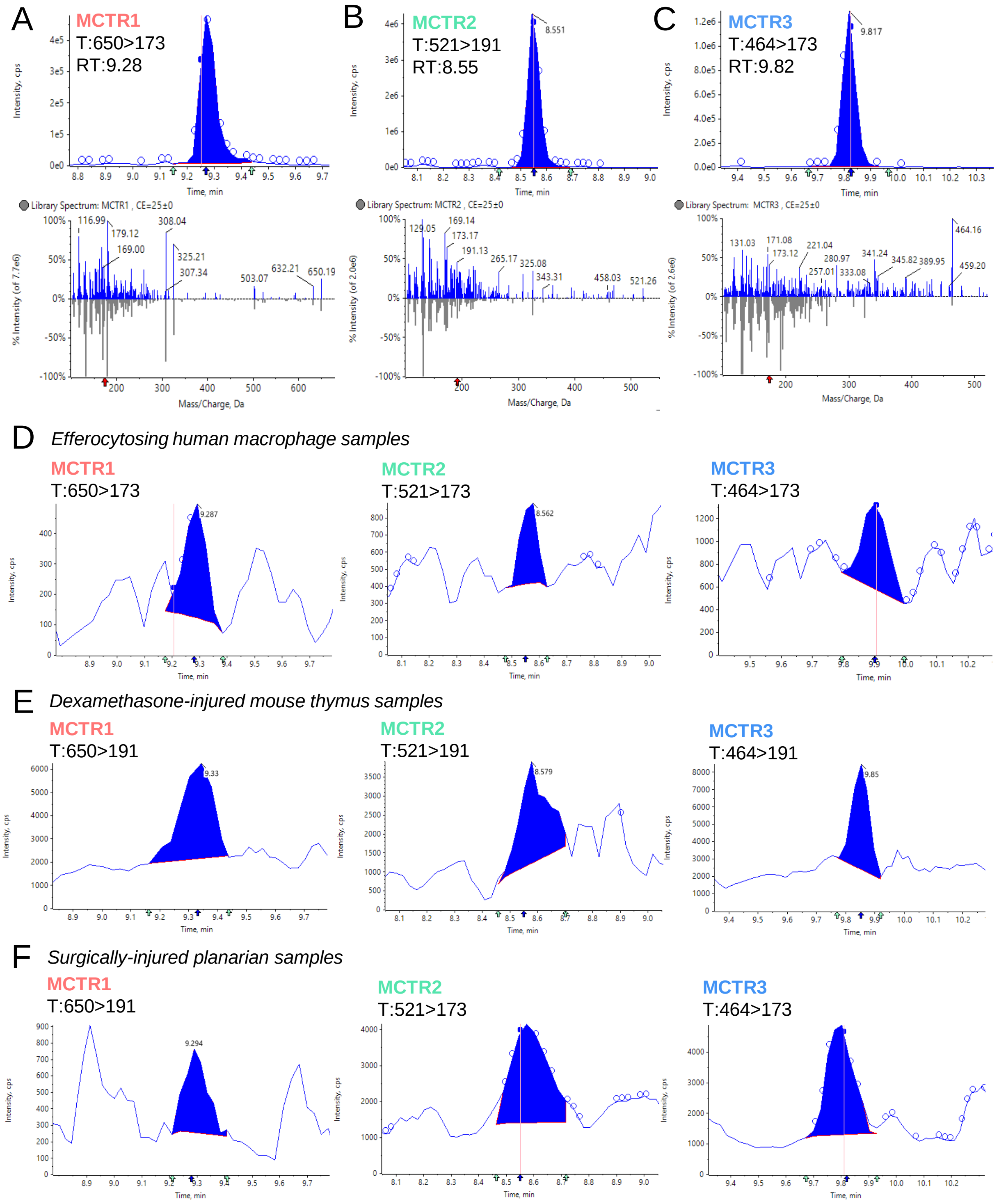

Figure S2; related to Figure 1-2.
(A-C) Representative sMRM chromatograms and MS/MS fragmentation spectra of standards used for identification of (A) MCTR1, (B) MCTR2, and (C) MCTR3. Inset text shows transitions *(T)* and retention times (*RT*) used for identification. MS/MS fragmentation spectra were automatically matched to published spectra for each MCTR using the SCIEX OS Library Candidate Search algorithm.
(D-F) Representative sMRM chromatograms used for identification of MCTRs in biological samples from (D) efferocytosing human macrophages, (E) dexamethasone-injured mouse thymus, or (F) surgically-injured planarian flatworms.

Figure S3

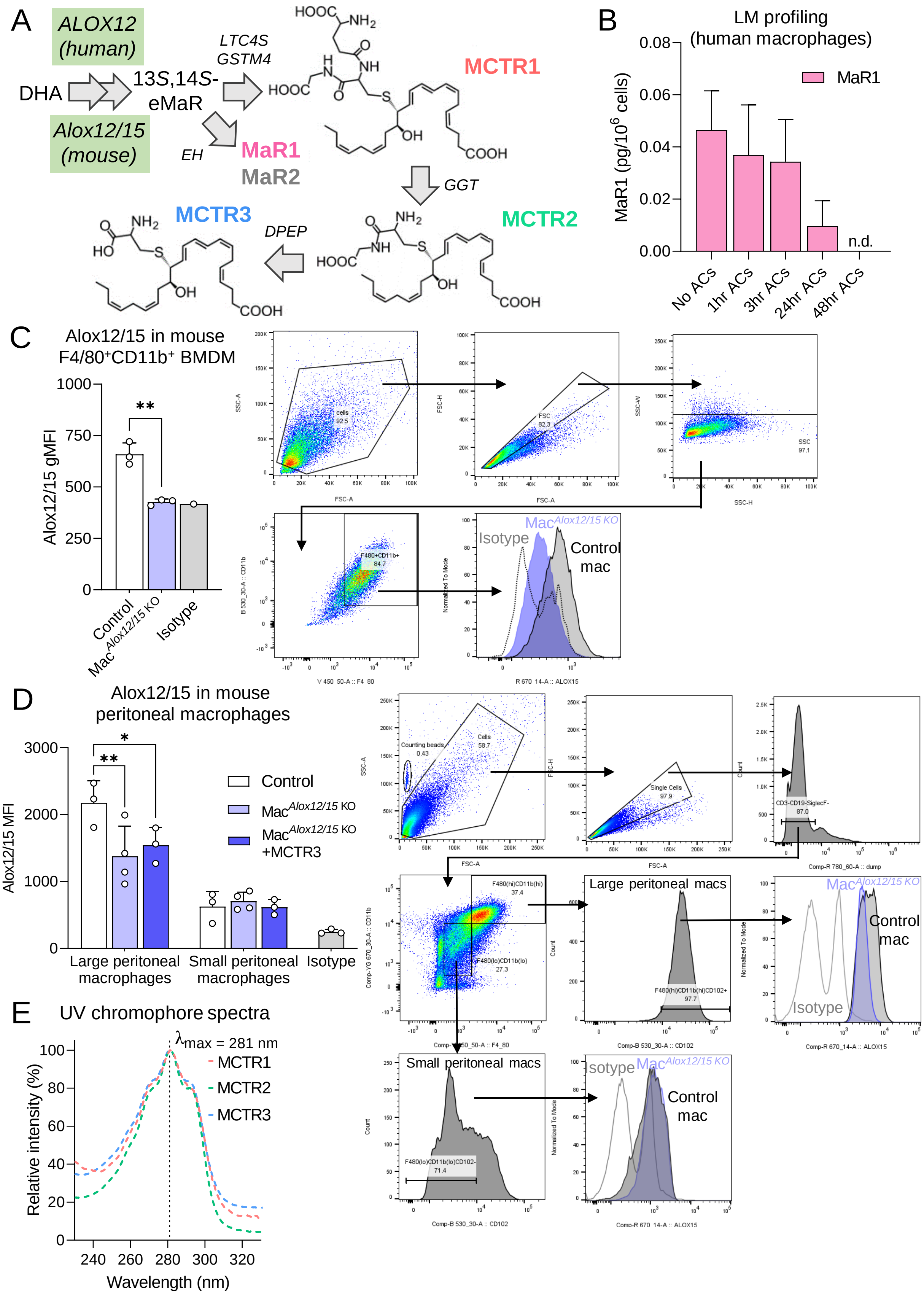
A
ALOX12 (human)
Alox12/15 (mouse)
DHA
13S,14S-eMaR
LTC4S GSTM4
EH
MaR1
MaR2
MCTR1
GGT
MCTR2
DPEP
MCTR3
B
LM profiling (human macrophages)
MaR1 (pg/10^6 cells)
MaR1
n.d.
No ACs
1hr ACs
3hr ACs
24hr ACs
48hr ACs
C
Alox12/15 in mouse F4/80+CD11b+ BMDM
Alox12/15 gMFI
Control
Mac Alox12/15 KO
Isotype
Isotype
Mac Alox12/15 KO
Control mac
D
Alox12/15 in mouse peritoneal macrophages
Alox12/15 MFI
Control
Mac Alox12/15 KO
Mac Alox12/15 KO +MCTR3
Large peritoneal macrophages
Small peritoneal macrophages
Isotype
Large peritoneal macs
Small peritoneal macs
Control mac
Isotype
E
UV chromophore spectra
λmax = 281 nm
MCTR1
MCTR2
MCTR3
Relative intensity (%)
Wavelength (nm)

Figure S3; related to Figure 1-2.
(A) Schematic overview of docosahexaenoic acid (DHA)-derived Maresin-1 (MaR1) and Maresin conjugates in tissue regeneration (MCTR) biosynthesis. *ALOX12* = human 12-lipoxygenase; *Alox12/15* = mouse 12/15-lipoxygenase; 13*S*,14*S*-eMaR = 13*S*,14*S*-epoxy-maresin; *LTC4S* = Leukotriene C4 Synthase; *GSTM4* = Glutathione S-Transferase Mu4; *GGT* = gamma-glutamyltransferase; *DPEP* = dipeptidase.
(B) Quantification of MaR1 by LC-MS/MS in lysates of human MoDMs incubated with ACs at 3:1 AC:macrophage ratio for indicated times. N=5 human donors.
(C) Flow cytometry analysis of Alox12/15 protein expression in BMDM from $\mathrm{Mac}^{\mathit{Alox12/15}\ \mathrm{KO}}$ or Alox12/15-floxed littermates (Control). Gating strategy is shown on the right. N=3 mice per group.
(D) Flow cytometry analysis of Alox12/15 protein expression in large ($CD3^{-}CD19^{-}SiglecF^{-}CD102^{+}CD11b^{hi}F4/80^{hi}$) and small ($CD3^{-}CD19^{-}SiglecF^{-}CD102^{-}CD11b^{med}F4/80^{lo}$) peritoneal macrophages from $\mathrm{Mac}^{\mathit{Alox12/15}\ \mathrm{KO}}$ or Alox12/15-floxed littermates (Control). Gating strategy is shown on the right. N=3 mice per group.
(E) UV chromophore spectra of MCTR1, MCTR2, and MCTR3 synthetic material obtained via total organic synthesis.

Data are shown as mean ± SD. Statistical significance was determined using one-way (B) or two-way (D) ANOVA with Holm-Sidak multiple comparison correction, or unpaired two-tailed Student's T-test (C). *p < 0.05, **p < 0.01, ***p < 0.001.

Figure S4

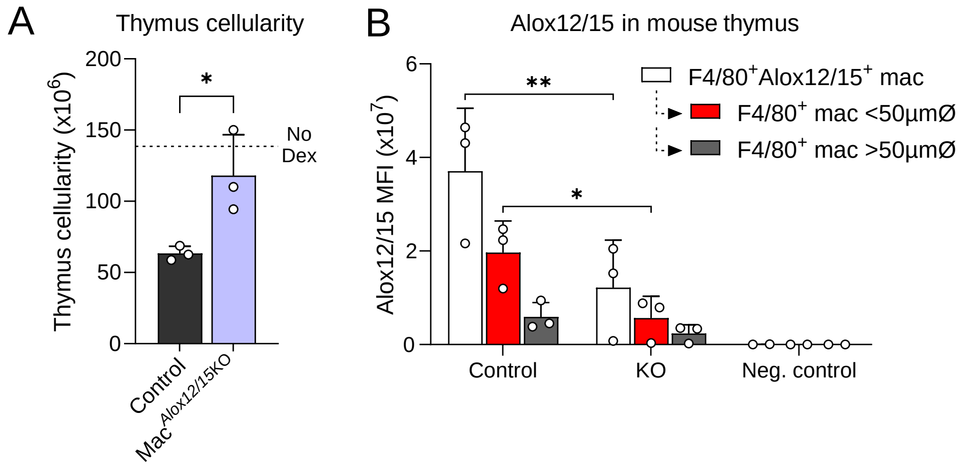

Figure S4; related to Figure 4.
(A-B) Dexamethasone (250µg) or DMSO vehicle was administered via i.p. injection to Mac$^{Alox12/15\ KO}$ or Alox12/15-floxed littermates (Control) for 18 hr; (A) thymus cellularity assessed by cell counting; (B) Immunofluorescence analysis of Alox12/15 protein expression in F4/80^{+} thymic macrophages, with unstained sections included as a negative control ("Neg. control"). N=3 mice per group.

Data are shown as mean ± SD. Statistical significance was determined using two-tailed Student's T-test (A) or two-way ANOVA with Holm-Sidak multiple comparison correction (B). *p < 0.05, **p < 0.01, ***p < 0.001 as indicated in the graphs in the graphs.

Figure S5

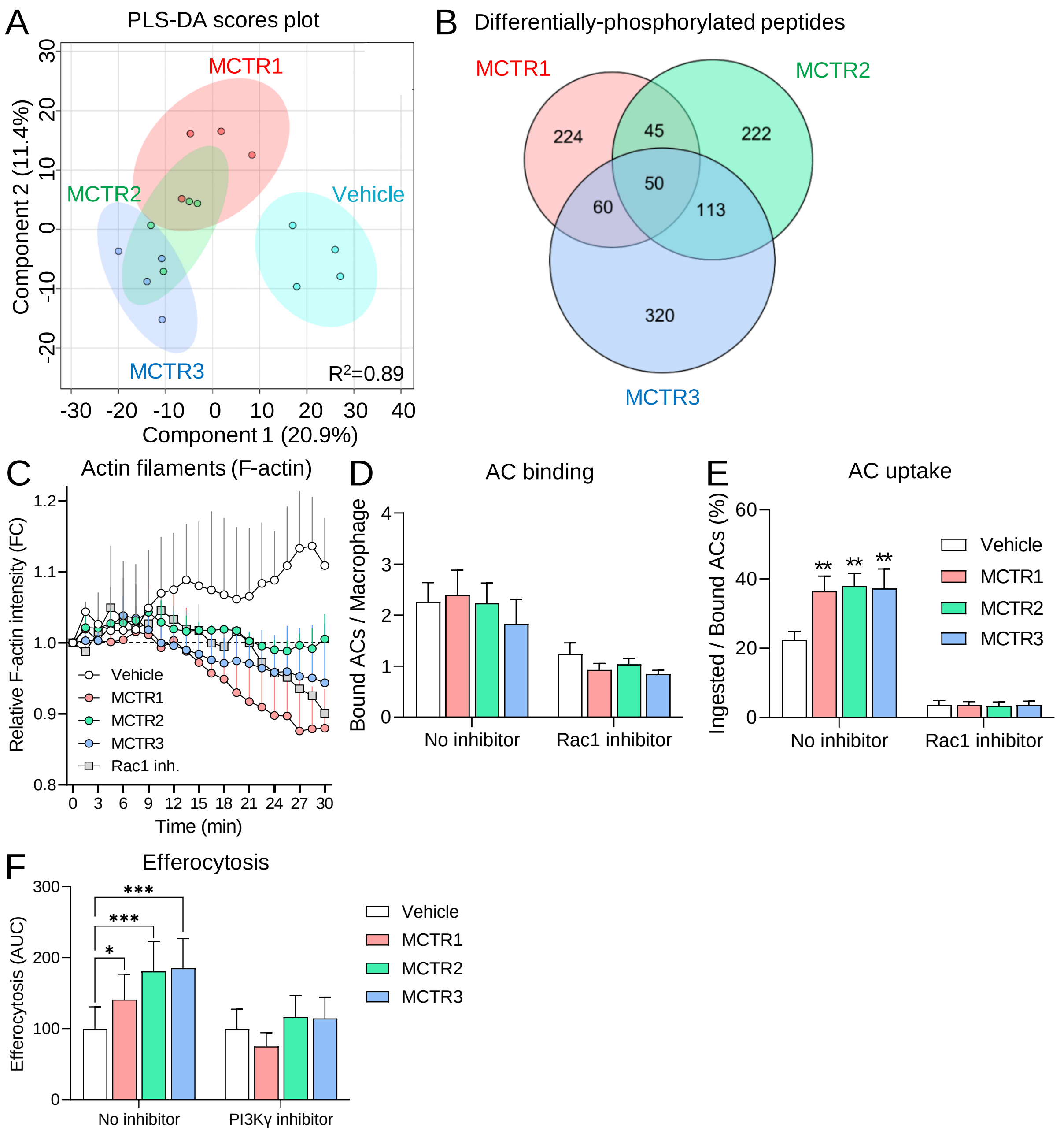

Figure S5; related to Figure 5.
(A-B) Phosphoproteomic profiling of human MoDMs treated with 1 nM MCTR1, MCTR2, MCTR3, or Vehicle (0.05% EtOH) for 5 to 15 min. N=4 human donors; (A) Partial least squares discriminant analysis (PLS-DA) of overall changes in phosphorylated peptide levels, R^2 = coefficient-of-determination; (B) Venn diagram of overlapping and distinct significantly differentially-regulated phosphopeptides between MCTR1, MCTR2, and MCTR3 treatments.
(C) Live measurement of F-actin fiber formation over time in SiR-actin-labelled MoDMs in response to 1 nM MCTR1, MCTR2, MCTR3, or Vehicle (0.05% EtOH) addition at time point 0 min. N=2 human donors.
(D-E) Quantification of number of ACs that were (D) bound but not ingested, or (E) ingested, during efferocytosis of pHrodo Red-labelled ACs (3:1 ratio) by MoDMs pre-incubated with 100 µM Rac1 inhibitor NSC23766 for 20 hr followed by 1 nM MCTR1, MCTR2, MCTR3, or Vehicle (0.05% EtOH) for 15 min. N=5 human donors.
(F) Efferocytosis of pHrodo Red-labelled ACs (3:1 ratio) by MoDMs pre-incubated with 1 µM PI3Kγ inhibitor (AS-604850) or No inhibitor control (0.01% EtOH) for 1 hr followed by 1 nM MCTR1, MCTR2, MCTR3, or Vehicle (0.05% EtOH) for 15 min. N=5 human donors.
Data are shown as mean ± SD. For (C-F), Statistical significance was determined using two-way ANOVA with Holm-Sidak multiple comparison correction. *$p < 0.05$, **$p < 0.01$, ***$p < 0.001$.

Figure S6

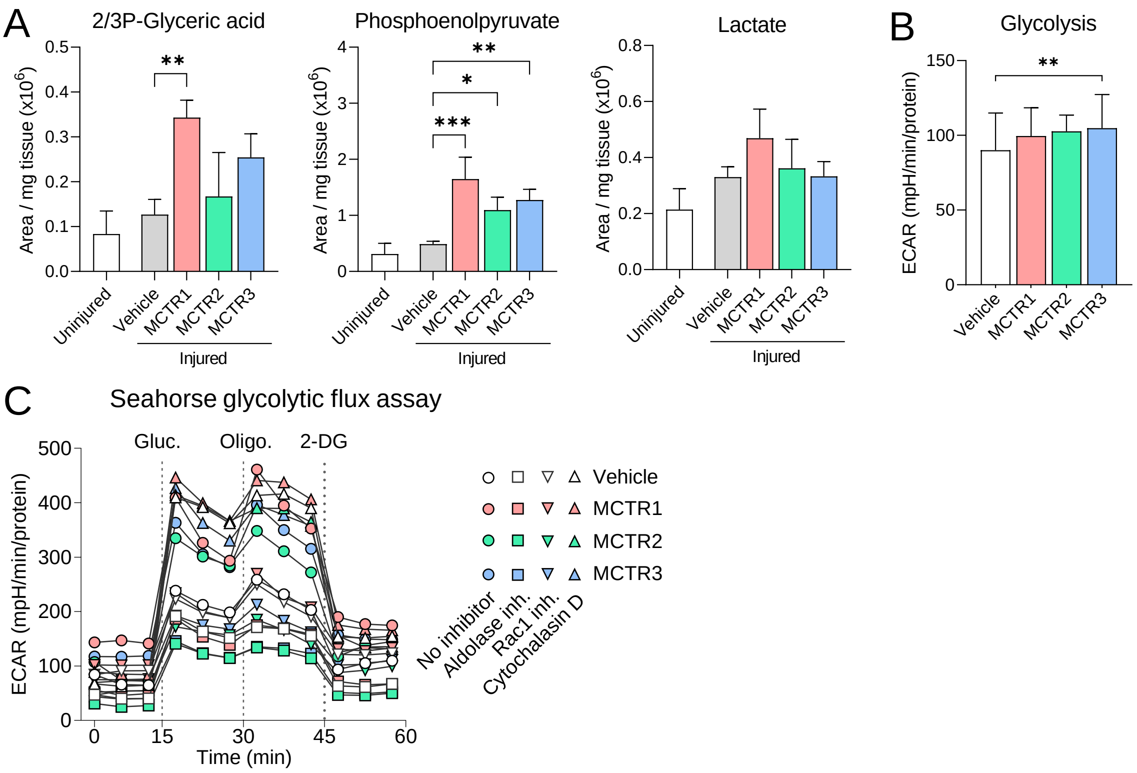

Figure S6; related to Figure 6.

(A) Steady-state levels of 2/3-phosphoglyceric acid, phosphoenolpyruvate, and lactate as determined by LC-MS/MS in uninjured or surgically-injured Planaria incubated with 10 nM MCTR1, MCTR2, MCTR3, or Vehicle (0.05% EtOH) for 3 hrs. N=3 independent replicates per group (20 worms per replicate).

(B) Glycolysis parameter values in human MoDMs incubated with 1 nM MCTR1, MCTR2, MCTR3, or Vehicle (0.05% EtOH) for 60 min, derived from Seahorse flux analysis ECAR values in Figure 6D. N=8 human donors.

(C) Seahorse flux analysis of glycolytic activity in human MoDMs pre-treated with 100 µM Rac1 inhibitor (NSC23766) for 18 hrs, or 10 µM aldolase inhibitor (Aldometanib) or 10 µM cytochalasin D for 2 hrs, followed by incubation with 1 nM MCTR1, MCTR2, MCTR3, or Vehicle (0.05% EtOH) for 1 hr. Extracellular acidification rate (ECAR) in response to sequential glucose (Gluc), oligomycin (Oligo), and 2-deoxyglucose (2-DG) injections. N=5 human donors.

Data are shown as mean ± SD (for A,B) or mean (for C). p-values were calculated using one-way ANOVA (A,B) with Holm-Sidak multiple comparison correction. *$p < 0.05$, **$p < 0.01$, ***$p < 0.001$ versus Vehicle control.

Table S1

KEGG ID	Gene	Modification	Peptide	log2FC MCTR1	p-val MCTR1	log2FC MCTR2	p-val MCTR2	log2FC MCTR3	p-val MCTR3
hsa:22848	AAK1	AAK1_T606	VQTTPPPAVQGQK	-0.205	0.267	-0.469	0.040	-0.259	0.142
hsa:26574	AATF	AATF_S203	AGDRNSEDDGVVMTFSSVK	-0.311	0.394	0.304	0.028	0.215	0.594
hsa:10057	ABCC5	ABCC5_S509	NATLAWDSSHSSIQNSPK	0.074	0.638	0.826	0.043	0.654	0.062
hsa:22885	ABLIM3	ABLIM3_S388	QGMSPTFSR	1.507	0.133	1.867	0.005	2.922	0.007
hsa:23527	ACAP2	ACAP2_S521	YSISLSPPEQQK	0.252	0.368	0.246	0.281	0.847	0.008
hsa:22985	ACIN1	ACIN1_S561	RASHTLLPSHR	-2.571	0.029	-6.154	0.002	-6.677	0.002
hsa:2182	ACSL4	ACSL4_S674	VRLSPEPWTPETGLVTDAFK	0.789	0.042	0.162	0.671	0.484	0.392
hsa:58	ACTA1	ACTA1_S54	HQGVMVGMGQKDSYVGDEAQSK	0.054	0.820	-0.018	0.832	-0.574	0.014
hsa:60	ACTB	ACTB_M305	KDLYANTVLSGGTTMYPGIADR	0.900	0.007	-0.044	0.907	0.268	0.489
hsa:6868	ADAM17	ADAM17_S791	SFEDLTDHPVTR	-0.097	0.743	-0.571	0.139	-0.772	0.050
hsa:8754	ADAM9	ADAM9_S758_T761	HVSPVTPPREVPIYANR	-0.368	0.306	-0.369	0.084	-0.320	0.027
hsa:8754	ADAM9	ADAM9_S758	HVSPVTPPR	-0.101	0.639	-0.620	0.011	-0.013	0.772
hsa:103	ADAR	ADAR_T603	TAESQTPTPSATSFFSGK	-0.162	0.001	0.196	0.319	0.363	0.346
hsa:120	ADD3	ADD3_S683	IEEVLSPEGSPSKSPSK	-0.112	0.560	-0.416	0.037	-0.223	0.207
hsa:27125	AFF4	AFF4_S703	ELLSPLSEPDDRYPLIVK	-0.173	0.454	0.290	0.542	0.387	0.043
hsa:54812	AFTPH	AFTPH_S411	KFTNFQSPNIDPTEENDLDDSLSVK	-0.865	0.013	-0.613	0.053	-0.790	0.029
hsa:116988	AGAP3	AGAP3_S538	LDPPPSPHSNR	-0.099	0.654	-0.472	0.068	-0.479	0.029
hsa:3267	AGFG1	AGFG1_S181	GTPSQSPVVGR	-0.160	0.383	0.376	0.045	0.156	0.441
hsa:3267	AGFG1	AGFG1_T177_S181	SLLGDSAPTLHLNKGTPSQSPVVGR	0.495	0.295	1.295	0.003	0.294	0.615
hsa:79026	AHNAK	AHNAK_S5749_S5752	SSKASLGSLEGEAEAEASSPK	0.626	0.028	1.180	0.007	0.579	0.029
hsa:79026	AHNAK	AHNAK_S5731	GKGGVTGSPEASISGSK	-0.618	0.153	-0.751	0.011	-0.951	0.030
hsa:79026	AHNAK	AHNAK_T4766	VDINTPDVDVHGPDWHLK	-0.562	0.034	-0.349	0.120	-0.712	0.034
hsa:79026	AHNAK	AHNAK_S5841	GHYEVTGSDDETGKLQGSGVSLASKK	-2.319	0.074	0.249	0.952	-2.614	0.038
hsa:79026	AHNAK	AHNAK_S5752	ASLGSLEGEAEAEASSPK	0.284	0.034	0.179	0.211	0.129	0.070
hsa:79026	AHNAK	AHNAK_S115	EVFSSCSSEVVLSGDDEEYQR	-0.820	0.933	2.142	0.027	0.000	0.356

hsa:51151	AIM1	AIM1_T525	MPLLELGGETTPPLSTER	-0.588	0.003	-0.521	0.001	-0.549	0.014
hsa:11215	AKAP11	AKAP11_S1242	SVSPTFLNPSDENLK	0.179	0.376	0.460	0.118	0.895	0.007
hsa:11215	AKAP11	AKAP11_S1019	VSMVHGSSLETLPSCPAVTGQK	0.016	0.741	0.739	0.279	0.783	0.043
hsa:11215	AKAP11	AKAP11_S18	SFSEDVFQSVK	0.330	0.185	0.700	0.029	0.557	0.091
hsa:11214	AKAP13	AKAP13_T2467	RAETFGGFDSHQMNASK	0.472	0.196	0.341	0.183	0.276	0.003
hsa:11214	AKAP13	AKAP13_S1929_S1932	FLSHSTDSLNK	0.228	0.213	0.459	0.101	0.559	0.012
hsa:11214	AKAP13	AKAP13_S1906	SVSIQNITGVGNDENMSNTWK	1.114	0.044	1.422	0.007	0.451	0.129
hsa:8227	AKAP17A	AKAP17A_S537	VVPEDGSPEKR	0.216	0.006	0.051	0.898	-0.463	0.357
hsa:11217	AKAP2	AKAP2_S152	DALGDSLQVPVSPSSTTSSR	0.146	0.488	0.809	0.222	1.193	0.004
hsa:8574	AKR7A2	AKR7A2_S255	FFGNSWAETYR	0.294	0.215	0.563	0.028	0.372	0.239
hsa:226	ALDOA	ALDOA seq: 61 - 69 + Gln->pyro-Glu (N-term Q)	QLLLTADDR	0.697	0.038	-0.304	0.629	0.140	0.836
hsa:54890	ALKBH5	ALKBH5_S361	RGSFSSENYWR	0.229	0.295	0.176	0.339	0.181	0.039
hsa:57679	ALS2	ALS2_S483_S492	RLSLPGLLSQVSPR	0.886	0.098	0.838	0.004	0.785	0.046
hsa:271	AMPD2	AMPD2_S190	AKQDFLKTDSDSDLQLYK	0.435	0.034	0.276	0.474	0.593	0.190
hsa:23141	ANKLE2	ANKLE2_S662	NNSPPTVGAFGHTR	0.309	0.202	0.414	0.043	0.550	0.015
hsa:57719	ANO8	ANO8_S641	RPGPSPEALLEEGSPTMVEK	-0.278	0.343	-0.206	0.465	-0.938	0.007
hsa:10541	ANP32B	ANP32B_T244	KRETDDEGEDD	3.031	0.041	4.453	0.012	4.848	0.011
hsa:55911	APOBR	APOBR_S111	HEVGSSAVEQTWGWGDGSSHGSQAERQDSGAGETAK	-2.272	0.044	0.081	0.599	-3.883	0.005
hsa:55738	ARFGAP1	ARFGAP1_S361	SSDSWEVWGSASTNR	0.431	0.361	0.619	0.047	0.538	0.218
hsa:10565	ARFGEF1	ARFGEF1_S1569	SVDIHDSIQPR	0.032	0.547	0.540	0.024	0.491	0.113
hsa:10564	ARFGEF2	ARFGEF2_S218	ELEKPIQSKPQSPVIQAAAVSPK	0.100	0.622	0.029	0.785	0.335	0.023
hsa:79658	ARHGAP10	ARHGAP10_S591	TPPDTTFPEPTCLSASPPNAPPR	-0.356	0.089	-0.040	0.439	-0.104	0.030
hsa:94134	ARHGAP12	ARHGAP12_T231_S240	ATTPPNQGRPDSPVYANLQELK	0.910	0.046	1.303	0.038	1.230	0.039
hsa:94134	ARHGAP12	ARHGAP12_T104	STENVNKLPELSSFGKPSSSVQGTGLIR	0.426	0.040	0.280	0.105	0.446	0.152
hsa:55114	ARHGAP17	ARHGAP17_S674_S676	SPSPPTQHTGQPPGQPSAPSQLSAPR	0.427	0.019	0.314	0.020	0.357	0.004
hsa:57584	ARHGAP21	ARHGAP21_S1671	RNSEGSELSCTEGSLTSSLDSR	0.297	0.063	0.488	0.013	0.349	0.227
hsa:9938	ARHGAP25	ARHGAP25_T442	TQTLPNRK	0.000	0.356	4.765	0.004	6.002	0.003
hsa:9938	ARHGAP25	ARHGAP25_S511	TSTYDNVPSLPGSPGEEASALSSQACDSK	-0.325	0.048	-0.358	0.220	-0.294	0.343
hsa:57514	ARHGAP31	ARHGAP31_S387	MHSTGTGSSCDLTK	0.001	0.913	-0.536	0.005	-0.701	0.009

hsa:393	ARHGAP4	ARHGAP4_S899	QGLGPASTTSPSPGPR	0.868	0.008	0.756	0.061	0.740	0.116
hsa:394	ARHGAP5	ARHGAP5_S1195	HRGSEEDPLLSPVETWK	-0.555	0.950	-0.414	0.683	-2.521	0.039
hsa:64333	ARHGAP9	ARHGAP9_S203	SPPPGPACPLLQR	0.215	0.297	0.131	0.389	0.164	0.002
hsa:397	ARHGDIB	ARHGDIB_S145	ATFMVGSYGPRPEEYEFLTPVEEAPK	0.537	0.011	-0.123	0.764	0.288	0.511
hsa:9826	ARHGEF11	ARHGEF11_S663_T668	SLENPTPPFTPK	0.579	0.181	0.975	0.179	1.216	0.047
hsa:9181	ARHGEF2	ARHGEF2_S129	ERPSSAIYPSDSFR	0.379	0.001	0.324	0.297	0.516	0.010
hsa:9181	ARHGEF2	ARHGEF2_S886	SLPAGDALYLSFNPPQPSR	0.103	0.232	0.341	0.406	0.272	0.027
hsa:9181	ARHGEF2	ARHGEF2_S174	ILSQSTDSLNMR	0.204	0.312	0.281	0.017	0.281	0.033
hsa:9181	ARHGEF2	ARHGEF2_T175	ILSQSTDSLNMR	0.169	0.410	0.408	0.027	0.336	0.168
hsa:9181	ARHGEF2	ARHGEF2_S956	LSPPHSPR	-0.466	0.049	0.004	0.984	-0.104	0.740
hsa:10095	ARPC1B	ARPC1B_S311	ASSEGGTAAGAGLDSLHK	-0.295	0.332	-0.259	0.354	-0.686	0.009
hsa:10776	ARPP19	ARPP19_S62	YFDSGDYNMAK	0.134	0.204	0.375	0.026	0.190	0.350
hsa:50807	ASAP1	ASAP1_S843	TLSDPPSPLPHGPPNK	0.243	0.092	0.264	0.053	0.419	0.001
hsa:50807	ASAP1	ASAP1_S1027	SHPLDLSPNVQSR	-0.160	0.380	-0.008	0.870	-0.407	0.015
hsa:9776	ATG13	ATG13_S361	ASPHDVLETIFVR	0.633	0.016	0.566	0.068	0.633	0.052
hsa:51062	ATL1	ATL1_S10	DRNSWGGFSEK	0.086	0.397	0.288	0.001	0.389	0.010
hsa:1822	ATN1	ATN1_S101	TEQELPRPQSPSDLDSLDGR	-0.234	0.047	0.122	0.481	0.032	0.873
hsa:490	ATP2B1	ATP2B1_S1216	NSSPPPSPNK	0.262	0.641	0.658	0.019	0.436	0.170
hsa:11273	ATXN2L	ATXN2L_S335	QGSGRESPSLASR	-0.287	0.609	-0.515	0.332	-1.408	0.026
hsa:11273	ATXN2L	ATXN2L_S559	LQPSSSPENSLDPFPPR	0.413	0.031	0.554	0.004	0.403	0.058
hsa:11273	ATXN2L	ATXN2L_S594	EKEVDGLLTSEPMGSPVSSK	0.655	0.196	0.955	0.020	0.570	0.233
hsa:23080	AVL9	AVL9_S246	KSMSEDGGLQESNPCADDFVSASTADVSHTNLGTIR	0.102	0.012	0.141	0.479	0.281	0.026
hsa:9531	BAG3	BAG3_S173	SQSPAASDCSSSSSSASLPSSGR	-0.175	0.360	0.380	0.042	-0.244	0.465
hsa:7917	BAG6	BAG6_S964	RVGDPPQPLPEEPMEVQGAERASPEPQR	-0.212	0.340	-0.122	0.453	-0.338	0.041
hsa:7917	BAG6	BAG6_S973	ENASPAPGTTAEEAMSR	-0.524	0.033	-0.236	0.264	-0.343	0.246
hsa:8314	BAP1	BAP1_S521	SANPTRPSSPVTSHISK	-0.244	0.215	-0.409	0.046	-0.088	0.669
hsa:10409	BASP1	BASP1_S164	KTEAPAAPAAQETKSDGAPASDSKPGSSEAAPSSK	-0.721	0.007	-0.199	0.649	0.151	0.572
hsa:11177	BAZ1A	BAZ1A_S1413	RQSPEPSPVTLGR	-0.201	0.441	0.245	0.285	0.281	0.011
hsa:10295	BCKDK	BCKDK_T32	STSATDTHHVEMAR	-0.395	0.064	-0.673	0.003	-0.490	0.114

hsa:83596	BCL2L12	BCL2L12_S242	LVRLSSDSFAR	0.026	0.935	-0.108	0.227	-0.151	0.028
hsa:83596	BCL2L12	BCL2L12_S273	ACPGPPPPSPEPLAR	-0.705	0.042	-0.507	0.212	-0.507	0.180
hsa:9774	BCLAF1	BCLAF1_S512	DLFDYSPPLHK	0.214	0.241	0.483	0.057	0.565	0.006
hsa:9774	BCLAF1	BCLAF1_S531	STFREESPLR	-0.308	0.107	-0.595	0.068	-0.607	0.020
hsa:9774	BCLAF1	BCLAF1_S285_S290	YSPSQNSPIHHIPSR	0.389	0.357	0.327	0.191	0.413	0.030
hsa:9774	BCLAF1	BCLAF1_S290	YSPSQNSPIHHIPSR	0.389	0.357	0.327	0.191	0.413	0.030
hsa:9774	BCLAF1	BCLAF1_S222	SSATSGDIWPGLSAYDNSPR	0.624	0.268	0.974	0.010	1.150	0.034
hsa:9774	BCLAF1	BCLAF1_S648	QKSPEIHR	1.050	0.332	1.652	0.070	1.872	0.040
hsa:9774	BCLAF1	BCLAF1_S177	KAEGEPQEESPLK	0.313	0.008	0.050	0.871	-0.274	0.565
hsa:51272	BET1L	BET1L_S9	AQSPGAVEEILDR	-0.452	0.014	-0.027	0.909	-0.240	0.407
hsa:29998	BICRA	GLTSCR1_S1413	GGSPAPLPAK	-0.642	0.024	-0.346	0.091	-0.743	0.045
hsa:51411	BIN2	BIN2_S406	RASIQR	2.877	0.151	3.660	0.024	4.571	0.023
hsa:51411	BIN2	BIN2_S357	SQEEVLPSSTTPSPGGALSPSGQPSSSATEVVLR	-0.595	0.013	-0.264	0.146	-0.253	0.048
hsa:51411	BIN2	BIN2_S395	TASEGSEQPK	1.376	0.046	2.262	0.030	2.377	0.574
hsa:57448	BIRC6	BIRC6_S577	SPATSPISSNSHR	0.008	0.584	-0.874	0.049	-0.283	0.139
hsa:55589	BMP2K	BMP2K_S14	SEGGSGGGAAGGGAGGAGAGAGCGSGGSSVGVR	0.060	0.768	-0.235	0.516	-0.817	0.032
hsa:673	BRAF	BRAF_S447	RDSSDDWEIPDGQITVGQR	-0.235	0.229	-0.029	0.836	0.162	0.046
hsa:23476	BRD4	BRD4_S470	MPDEPEEPVVAVSSPAVPPPTK	-0.121	0.015	0.088	0.767	0.179	0.677
hsa:2972	BRF1	BRF1_S553	GLSSAGGGSPHREDAQPEHSASAR	-1.162	0.110	-1.471	0.039	-1.754	0.024
hsa:9024	BRSK2	BRSK2_T431	VTPHPSPR	-0.466	0.049	0.002	0.992	-0.104	0.740
hsa:695	BTK	BTK_S174	NGSLKPGSSHR	0.496	0.219	0.071	0.280	-2.096	0.023
hsa:84811	BUD13	BUD13_S214	RPQHNSSGASPR	1.664	0.108	2.419	0.024	2.582	0.015
hsa:84811	BUD13	BUD13_S127	HDTPDSSPR	0.998	0.223	1.292	0.098	1.778	0.017
hsa:84811	BUD13	BUD13_S126_S127	HDTPDSSPR	3.071	0.033	4.700	0.017	4.650	0.058
hsa:283897	C16orf54	C16orf54_S194	QRPGSPDPEWGLQPR	-0.154	0.555	-0.160	0.345	-0.164	0.027
hsa:9854	C2CD2L	C2CD2L_S662	QKEAGLSQSHDDLSNATATPSVR	-0.814	0.028	-0.471	0.100	-1.219	0.091
hsa:9847	C2CD5	C2CD5_S260	LSSPAAFLPACNSPSK	0.383	0.095	0.567	0.079	0.962	0.008
hsa:728	C5AR1	C5AR1_S332	NVLTEESVVRESK	-0.243	0.216	-0.094	0.597	-0.634	0.029
hsa:79886	CAAP1	CAAP1_S312	SVNEILGLAESSPNEPK	-0.261	0.013	0.112	0.437	0.080	0.736

hsa:813	CALU	CALU_S69	TFDQLTPEESKER	-0.172	0.223	-0.388	0.038	-0.163	0.266
hsa:10645	CAMKK2	CAMKK2_S100	KLSLQER	0.435	0.587	0.235	0.629	-0.363	0.035
hsa:157922	CAMSAP1	CAMSAP1_S793	SSPCLSTASQMSSVSMASGSVK	1.449	0.010	0.990	0.036	0.668	0.272
hsa:822	CAPG	CAPG seq: 97 - 112 no mod	EVQGNESDLFMSYFPR	0.697	0.021	-0.385	0.652	0.137	0.819
hsa:64170	CARD9	CARD9_S460	RSQELSLPQDLEDTQLSDKGCLAGGGSPK	-0.172	0.025	-0.002	0.984	0.198	0.196
hsa:23589	CARHSP1	CARHSP1_S30	ERSPSPLR	-0.543	0.026	-0.160	0.612	-0.565	0.196
hsa:57091	CASS4	CASS4_S289	SLTPQLNNNVPMQK	0.023	0.875	0.183	0.029	0.165	0.334
hsa:10951	CBX1	CBX1_S89	KADSDSEDKGEESKPK	0.786	0.118	1.601	0.075	2.467	0.011
hsa:55749	CCAR1	CCAR1_T627	EISTPTHWSK	0.364	0.326	0.721	0.016	0.804	0.012
hsa:57805	CCAR2	CCAR2_S678	SVASNQSEMEFSSLQDMPK	-0.873	0.082	-0.219	0.013	-0.109	0.596
hsa:8030	CCDC6	CCDC6_S419	RSNSPDKFK	1.511	0.167	2.074	0.048	2.282	0.034
hsa:729440	CCDC61	CCDC61_S373_S376	LGSGGSGDGPSVSWSR	1.068	0.011	1.116	0.007	1.091	0.042
hsa:79080	CCDC86	CCDC86_S47	ALVEFESNPEETREPGSPPSVQR	-0.258	0.085	0.143	0.034	0.089	0.281
hsa:79080	CCDC86	CCDC86_S91	LQQGAGLESPQGQPEPGAASPQR	-0.505	0.024	-0.074	0.779	-0.262	0.530
hsa:55704	CCDC88A	CCDC88A_S1020	MVQSSPPISGEDNKWER	-0.233	0.452	0.019	0.901	0.141	0.044
hsa:55704	CCDC88A	CCDC88A_S1566	DTTSFEDISPQGVSDDSSTGSR	0.940	0.016	0.305	0.324	0.421	0.278
hsa:283234	CCDC88B	CCDC88B_S436	SLEPPPGSPGEAPLAGAAPSLQDEVR	-0.411	0.000	-0.206	0.243	-0.248	0.345
hsa:8812	CCNK	CCNK_S340	AVVVSPK	0.100	0.558	-0.261	0.447	-0.500	0.041
hsa:57018	CCNL1	CCNL1_S374	QQASKSPYNGVR	-0.160	0.456	-0.302	0.206	-0.664	0.039
hsa:23607	CD2AP	CD2AP_S510	FNGGHSPTHSPEK	0.012	0.925	0.247	0.564	0.650	0.032
hsa:10421	CD2BP2	CD2BP2_S195	KGPGQPSSPQRLDR	-0.969	0.041	-0.673	0.059	-1.280	0.006
hsa:960	CD44	CD44_S697_S706	KPSGLNGEASKSQEMVHLVNK	-0.511	0.036	-0.699	0.016	-0.709	0.008
hsa:960	CD44	CD44_S706	KPSGLNGEASKSQEMVHLVNK	-0.721	0.125	-0.832	0.091	-0.945	0.023
hsa:960	CD44	CD44_S697	LVINSGNGAVEDRKPSGLNGEASK	-0.904	0.061	-0.695	0.146	-1.107	0.040
hsa:921	CD5	CD5_S482	SSMQPDNSSDSDYDLHGAQR	-0.378	0.010	-0.012	0.913	-0.094	0.466
hsa:9578	CDC42BPB	CDC42BPB_S1690	HSTPSNSSNPSGPPSPNSPHR	-0.063	0.703	-0.521	0.136	-0.649	0.013
hsa:984	CDK11B	CDK11B_S589	EYGSPLK	0.387	0.036	0.369	0.127	0.724	0.000
hsa:984	CDK11B	CDK11B_S740	GTSPRPPEGGLGYSQLGDDDLK	-0.047	0.658	0.141	0.016	0.189	0.346
hsa:51755	CDK12	CDK12_S334	SSSPFLSK	-0.387	0.288	-0.052	0.784	-0.481	0.038

hsa:51755	CDK12	CDK12_S423	GSPVFLPR	0.079	0.597	0.172	0.034	0.127	0.284
hsa:51755	CDK12	CDK12_S1083	NSSPAPPQPAPGK	-0.697	0.040	-0.183	0.416	-0.180	0.284
hsa:51755	CDK12	CDK12_S333_S334	SSSPFLSK	0.229	0.162	0.575	0.012	0.178	0.502
hsa:8621	CDK13	CDK13_T1246	ILELTPEPDRPR	-0.014	0.680	2.084	0.536	1.943	0.000
hsa:5218	CDK14	CDK14_S95	VHSENNACINFK	0.106	0.087	0.310	0.035	0.378	0.268
hsa:1022	CDK7	CDK7_T170	AYTHQVVTR	-0.019	0.618	0.073	0.205	0.253	0.016
hsa:1025	CDK9	CDK9_S347	KGSQITQQSTNQSR	0.514	0.414	0.165	0.532	0.439	0.030
hsa:1051	CEBPB	CEBPB_S231	AYLGYQAVPSGSSGSLSTSSSSSPPGTPSPADAK	0.100	0.019	-0.113	0.804	-0.315	0.311
hsa:1062	CENPE	CENPE_S46	SFNFDR	0.115	0.590	0.329	0.025	0.651	0.003
hsa:1069	CETN2	CETN2_S20	RMSPKPELTEEQK	-0.426	0.112	-0.548	0.079	-0.595	0.048
hsa:283489	CHAMP1	CHAMP1_S476	GGSPDLWK	0.219	0.035	0.122	0.072	0.276	0.089
hsa:54927	CHCHD3	CHCHD3_S50	SQRYSGAYGASVSDEELK	-0.246	0.185	-0.162	0.028	0.002	0.987
hsa:1108	CHD4	CHD4_S1537	KMSQPGSPSPK	-0.682	0.523	-2.158	0.066	-4.349	0.005
hsa:1108	CHD4	CHD4_S1535	MSQPGSPSPK	0.743	0.054	0.612	0.107	1.046	0.013
hsa:57680	CHD8	CHD8_S2046	VSPSDTTPLVSR	-0.367	0.049	0.038	0.881	0.022	0.919
hsa:26973	CHORDC1	CHORDC1_S200	KTSDFNTFLAQEGCTK	0.282	0.353	0.451	0.156	0.314	0.033
hsa:54480	CHPF2	CHPF2_S64	ARLDQSDEDFKPR	-0.240	0.036	0.042	0.791	-0.235	0.518
hsa:23122	CLASP2	CLASP2_S596	VLNTGSDVEEAVADALKKPAR	0.483	0.017	0.333	0.306	0.684	0.076
hsa:11129	CLASRP	CLASRP_S294	RDSPTYDPYKR	0.977	0.038	0.796	0.175	1.367	0.019
hsa:11129	CLASRP	CLASRP_S285	KISPPSYAR	0.145	0.156	0.045	0.798	0.249	0.041
hsa:9685	CLINT1	CLINT1_S299	TIDLGAAAHYTGDKASPDQNASTHTPQSSVK	0.428	0.274	0.689	0.044	0.702	0.040
hsa:57396	CLK4	CLK4_S138	SIEDDEEGHLICQSGDVLR	0.674	0.012	0.541	0.079	0.945	0.003
hsa:1201	CLN3	CLN3_S12	RFSDSEGEETVPEPR	0.602	0.019	0.649	0.055	0.538	0.045
hsa:1207	CLNS1A	CLNS1A_S102	FEEESKEPVADEEEEDSDDDVEPITEFR	0.553	0.085	1.121	0.021	1.351	0.018
hsa:1213	CLTC	CLTC_T394	GILRTPDTIR	-0.057	0.421	-0.379	0.010	-0.265	0.206
hsa:26505	CNNM3	CNNM3_S700	TTTAAGSSHSRPGVPVEGSPGRNPGV	0.358	0.328	0.554	0.017	0.585	0.069
hsa:8161	COIL	COIL_S271	NSSEKLPTELSKEEPSTK	-0.372	0.020	-0.203	0.264	-0.235	0.299
hsa:1362	CPD	CPD_T1370	SLLSHEFQDETDTEEETLYSSKH	-0.692	0.050	-0.554	0.001	-0.375	0.247
hsa:11052	CPSF6	CPSF6_T407	EMDTARTPLSEAEFEEIMNR	-1.023	0.047	0.399	0.507	0.378	0.451

hsa:1385	CREB1	CREB1_S108	LFSGTQISTIAESEDSQESVDSVTDSQK	-0.317	0.326	-0.899	0.038	-1.334	0.006
hsa:79174	CRELD2	CRELD2_S70	TLSKYESSEIR	0.067	0.872	0.651	0.024	0.716	0.218
hsa:200186	CRTC2	CRTC2_S178	TSSDSALHTSVMNPSPQDTYPGPTPPSILPSR	-0.354	0.859	-0.412	0.077	-0.194	0.041
hsa:200186	CRTC2	CRTC2_S613	HGSGPNIILTGDSSPGFSK	0.766	0.040	0.681	0.082	0.745	0.125
hsa:64784	CRTC3	CRTC3_S370	LFSLSNPSLSTTNLSGPSR	0.751	0.045	0.883	0.003	0.342	0.251
hsa:1465	CSRP1	CSRP1_S192	GFGFGQGAGALVHSE	0.331	0.214	0.437	0.032	0.496	0.101
hsa:1479	CSTF3	CSTF3_S691	RPNEDSDEDEEKGAVVPPVHDIYR	-0.908	0.027	-0.310	0.294	-0.531	0.172
hsa:4253	CTAGE5	CTAGE5_S517	EHSPYGPSPLGWPSSETR	-0.743	0.237	-1.365	0.022	-1.138	0.042
hsa:1500	CTNND1	CTNND1_S232	HYEDGYPGGSDNYGSLSR	2.556	0.030	3.097	0.186	3.021	0.430
hsa:8450	CUL4B	CUL4B_S146	MAEESSSSSSSSPTAATSQQQQLK	-0.236	0.149	0.546	0.161	0.615	0.030
hsa:1535	CYBA	CYBA_S168	KKPSEEEAAVAAGGPPGGPQVNPIPVTDEVV	-0.186	0.164	-0.042	0.012	-0.255	0.264
hsa:1616	DAXX	DAXX_S495	DGDKSPMSSLQISNEK	1.596	0.002	0.354	0.188	2.164	0.130
hsa:55827	DCAF6	DCAF6_S649	DSALQDTDDSDDDPVLIPGAR	-0.255	0.037	-0.206	0.095	-0.176	0.005
hsa:79877	DCAKD	DCAKD_S149	RNSLNR	0.580	0.281	1.390	0.042	0.342	0.480
hsa:55802	DCP1A	DCP1A_S525	KASSPSPLTIGTPESQR	-1.230	0.074	-0.654	0.275	-1.749	0.015
hsa:55802	DCP1A	DCP1A_S353	SPLLNQPVPELSHASLIANQSPFR	0.016	0.906	0.259	0.006	0.243	0.056
hsa:1643	DDB2	DDB2_S26	SRSPLELEPEAK	0.268	0.021	0.259	0.265	0.226	0.166
hsa:84301	DDI2	DDI2_S128	QPPGTQQSHSSPGEITSSPQGLDNPALLR	0.401	0.075	0.509	0.011	0.224	0.348
hsa:9188	DDX21	DDX21_S121	VTKNEEPSEEEIDAPKPK	0.466	0.032	0.363	0.523	-1.300	0.267
hsa:9416	DDX23	DDX23_S14	DRDASPSKEER	0.895	0.208	1.803	0.106	2.448	0.047
hsa:9416	DDX23	DDX23_S109	SSLSPGR	0.411	0.668	0.435	0.010	0.084	0.843
hsa:11325	DDX42	DDX42_S754	AGSSAAGASGWTSAGSLNSVPTNSAQQGHNSPDSPVTSAAK	0.232	0.549	0.729	0.060	0.799	0.007
hsa:79039	DDX54	DDX54_S782	QKIDDRDSDEEGASDR	0.454	0.026	0.233	0.741	-0.582	0.337
hsa:55667	DENND4C	DENND4C_S732_S737	HSQPSPEPHSPTEPPAWGSSIVK	-0.296	0.403	-0.596	0.087	-0.840	0.029
hsa:1676	DFFA	DFFA_S315	SISASKASPPGDLQNPK	-0.064	0.638	0.278	0.332	0.391	0.005
hsa:8525	DGKZ	DGKZ_S37	RRSPAGQASSSLAQR	0.279	0.224	0.528	0.108	0.927	0.007
hsa:8525	DGKZ	DGKZ_S53	RSSAQLQGCLLSCGVR	0.077	0.879	0.538	0.022	0.425	0.051
hsa:1660	DHX9	DHX9_S87	SEEVPAFGVASPPPLTDTPDTTANAEGDLPTTMGGPLPPHLALK	-0.679	0.049	-0.123	0.795	-0.104	0.816
hsa:11083	DIDO1	DIDO1_S1040	SPPEGDTTLFLSR	0.482	0.233	0.793	0.043	1.206	0.028

hsa:11083	DIDO1	DIDO1_T1256_S1260	KPSKYPLCSADAAVSTTPPGSPPPPPPLPEPPVLK	0.586	0.520	0.995	0.034	0.472	0.263
hsa:1736	DKC1	DKC1_S451_S455	KRESESESDETPPAAPQLIK	0.155	0.359	0.380	0.048	0.559	0.203
hsa:1657	DMXL1	DMXL1_S574	STSMLISSGHNK	0.493	0.013	0.371	0.153	0.484	0.010
hsa:123872	DNAAF1	DNAAF1_T269	RTVTVR	0.912	0.179	1.583	0.001	1.738	0.000
hsa:25981	DNAH1	DNAH1_T2574	ISRTLR	0.460	0.253	0.553	0.027	0.082	0.931
hsa:1785	DNM2	DNM2_S764	EALNIIGDISTSTVSTPVPPPVDDTWLQSASSHSPTPQR	-0.618	0.040	-0.318	0.406	0.165	0.620
hsa:1794	DOCK2	DOCK2_S1685	VEQEEPISPGSTLPEVK	0.112	0.608	0.279	0.043	0.230	0.036
hsa:1794	DOCK2	DOCK2_S1592	RVSDNLRPFHDR	-0.871	0.174	-0.802	0.214	-1.439	0.049
hsa:80005	DOCK5	DOCK5_S1834	NSTELAPPLPVR	-0.209	0.339	0.271	0.018	-0.194	0.301
hsa:85440	DOCK7	DOCK7_S864	LPNTYPNSSSPGPGGLGGSVHYATMAR	0.353	0.050	0.235	0.378	0.147	0.226
hsa:81704	DOCK8	DOCK8_S139	NQGSPEICGFKK	-0.340	0.050	0.305	0.438	0.380	0.018
hsa:81704	DOCK8	DOCK8_S904	VMSSSNPDLAGTHSAADEEVK	0.138	0.502	0.184	0.393	0.307	0.028
hsa:9980	DOP1B	DOPEY2_S1193_S1194	VDSDKTQASESFSSDEEADLELQALTTSR	0.117	0.288	0.195	0.250	0.482	0.021
hsa:23033	DOPEY1	DOPEY1_S1238	NSSSPCISGTTHTLHDSSVASIETK	-0.483	0.061	-0.549	0.198	-1.124	0.013
hsa:84062	DTNBP1	DTNBP1_S316_S321	DISEGGESPVVQSDEEEVQVDTALATSHTDR	1.503	0.038	1.807	0.026	1.050	0.048
hsa:151636	DTX3L	DTX3L_S539	AASPPLKGSVSSEASELDKK	-0.469	0.295	-0.386	0.365	-1.026	0.018
hsa:1778	DYNC1H1	DYNC1H1_S4368	TDSTSDGRPAWMR	0.147	0.315	0.455	0.069	0.549	0.017
hsa:51143	DYNC1LI1	DYNC1LI1_S207	DFQEYVEPGEDFPASPQRR	0.230	0.501	1.544	0.008	0.787	0.185
hsa:9149	DYRK1B	DYRK1B_Y273	IYQYIQSR	0.562	0.009	0.422	0.352	0.377	0.328
hsa:8445	DYRK2	DYRK2_T381	VYTYIQSR	0.194	0.480	0.705	0.179	0.477	0.005
hsa:8445	DYRK2	DYRK2_Y382	VYTYIQSR	0.239	0.383	0.702	0.175	0.479	0.010
hsa:9166	EBAG9	EBAG9_S36	KLSGDQITLPTTVDYSSVPK	0.019	0.934	0.211	0.110	0.373	0.030
hsa:1933	EEF1B2	EEF1B2_S106	YGPADVEDTTGSGATDSKDDDDIDLFGSDDEEESEEAK	0.693	0.005	0.607	0.094	0.551	0.212
hsa:1936	EEF1D	EEF1D_S162	KPATPAEDDEDDDIDLFGSDNEEEDKEAAQLR	0.625	0.028	1.181	0.007	0.580	0.029
hsa:79180	EFHD2	EFHD2_S74	RADLNQGIGEPQSPSRR	-0.888	0.011	-0.184	0.523	-0.793	0.023
hsa:254102	EHBP1L1	EHBP1L1_S1273	AHGSFSHVR	-1.339	0.232	-4.401	0.004	-3.429	0.008
hsa:8662	EIF3B	EIF3B_S83	TEPAAEAEAASGPSESPSPPAAEELPGSHAEPPVPAQGEAPGEQAR	-0.038	0.566	0.190	0.029	0.422	0.395
hsa:728689	EIF3CL	EIF3CL_S39	QPLLLSEDEEDTKR	1.214	0.091	0.937	0.045	0.692	0.214
hsa:1975	EIF4B	EIF4B_S497	SQSSDTEQQSPTSGGGK	0.661	0.040	0.709	0.126	0.459	0.076

hsa:1975	EIF4B	EIF4B_S498_T500	SQSSDTEQQSPTSGGGK	2.052	0.051	2.645	0.040	2.732	0.095
hsa:1978	EIF4EBP1	EIF4EBP1_T70	TPPRDLPTIPGVTSPSSDEPPMEASQSHLR	-0.424	0.030	0.094	0.940	0.266	0.139
hsa:1981	EIF4G1	EIF4G1_S1231	EAALPPVSPLK	0.178	0.048	0.303	0.016	0.412	0.320
hsa:8672	EIF4G3	EIF4G3_S232	SPSPVLR	-0.132	0.368	-0.364	0.035	-0.058	0.697
hsa:1983	EIF5	EIF5_S389_S390	EAEEESSGGEEEDEDENIEVVYSK	0.998	0.928	3.097	0.024	2.209	0.182
hsa:9669	EIF5B	EIF5B_S113	KQSFDDNDSEELEDKDSK	0.618	0.073	0.288	0.543	-0.453	0.002
hsa:9669	EIF5B	EIF5B_S107_S113	KQSFDDNDSEELEDKDSK	0.545	0.045	0.807	0.462	-0.463	0.405
hsa:1997	ELF1	ELF1_S168	YADSPGASSPEQPKR	-0.111	0.073	0.187	0.029	0.256	0.006
hsa:1997	ELF1	ELF1_S187	KTKPPRPDSPATTPNISVK	-0.701	0.049	-0.349	0.327	-0.558	0.110
hsa:2000	ELF4	ELF4_S188	STSPVTDPSIPIR	-0.192	0.028	0.146	0.263	0.061	0.766
hsa:27436	EML4	EML4_T897_T899	APVSSTESVIQSNTPTPPPSQPLNETAEEESR	-0.119	0.038	0.113	0.566	0.196	0.589
hsa:64772	ENGASE	ENGASE_S66	EVVSFSPDPLPVR	0.568	0.043	0.627	0.281	0.774	0.165
hsa:2036	EPB41L1	EPB41L1_S541	RLPSSPASPSPK	-0.199	0.026	-0.058	0.739	-0.137	0.609
hsa:2037	EPB41L2	EPB41L2_T600	SPTKAPHLQLIEGK	-0.277	0.163	0.066	0.420	-0.587	0.006
hsa:2037	EPB41L2	EPB41L2_S499	LVSPEQPPK	-0.145	0.235	-0.051	0.270	-0.315	0.028
hsa:23136	EPB41L3	EPB41L3_S762	LSTSPVR	1.840	0.084	2.191	0.041	2.666	0.027
hsa:22905	EPN2	EPN2_S173	GSSQPNLSTSHSEQEYGK	-0.565	0.016	-0.321	0.293	-0.570	0.130
hsa:58513	EPS15L1	EPS15L1_S229	KTVFPGAVPVLPASPPPKDSLR	0.126	0.030	-0.492	0.732	-0.074	0.560
hsa:23085	ERC1	ERC1_T38	TNSTGGSSGSSVGGGSGK	1.555	0.051	1.633	0.050	2.064	0.091
hsa:2073	ERCC5	ERCC5_S526	ELTPASPTCTNSVSK	-0.695	0.030	0.051	0.629	0.021	0.924
hsa:10613	ERLIN1	ERLIN1_S324	ESSLPSK	0.690	0.096	0.710	0.026	0.602	0.111
hsa:57488	ESYT2	ESYT2_S693_S699_T701	KTSIKSHMSGSPGPGGSNTAPSTPVIGGSDKPGMEEK	-1.293	0.037	-1.030	0.085	-1.449	0.069
hsa:57488	ESYT2	ESYT2_T701	SHMSGSPGPGGSNTAPSTPVIGGSDKPGMEEK	-0.253	0.025	0.035	0.888	-0.022	0.924
hsa:2119	ETV5	ETV5_S425	LSRSLR	0.912	0.179	1.583	0.001	1.738	0.000
hsa:2120	ETV6	ETV6_S203	SPLDNMIR	0.418	0.038	0.294	0.304	0.337	0.354
hsa:55770	EXOC2	EXOC2_S432	GSSFQSGR	-0.399	0.401	-1.538	0.084	-2.511	0.005
hsa:116224	FAM122A	FAM122A_S189	SQSPINCIRPSVLGPLK	1.037	0.062	1.600	0.000	0.568	0.086
hsa:116496	FAM129A	FAM129A_T584	KHNLFEDNMALPSESVSSLTDLKPPTGSNQASPAR	-0.400	0.149	-0.873	0.018	-1.337	0.005
hsa:64855	FAM129B	FAM129B_S665_S681	GLLAQGLRPESPPPAGPLLNGAPAGESPQPK	-0.027	0.764	-0.381	0.114	-0.984	0.004

hsa:64855	FAM129B	FAM129B_S665	GLLAQGLRPESPPPAGPLLNGAPAGESPQPK	-0.234	0.341	-0.539	0.024	-0.631	0.125
hsa:64855	FAM129B	FAM129B_S692_S696	AAPEASSPPASPLQHLLPGK	0.910	0.035	0.845	0.028	0.036	0.969
hsa:8603	FAM193A	FAM193A_S1144	LVLAESPQPK	-0.442	0.296	-0.396	0.047	-0.419	0.021
hsa:8603	FAM193A	FAM193A_S293	LILTDSGSAPTFCSDDEDVAPLSAK	-0.296	0.315	0.148	0.012	0.163	0.218
hsa:23272	FAM208A	FAM208A_S979	SSDYQFPSSPFTDTLK	0.642	0.014	0.548	0.064	-0.085	0.623
hsa:203259	FAM219A	FAM219A_S115	GYSSLDQSPDEKPLVALDTDSDDDFDMSR	-0.225	0.002	0.188	0.680	0.191	0.561
hsa:84895	FAM73B	FAM73B_S276	TLMLPLTEGSLR	0.339	0.028	0.386	0.049	0.488	0.089
hsa:143684	FAM76B	FAM76B_S193	ISNLSPEEEQGLWK	-0.296	0.197	-0.120	0.418	-0.153	0.042
hsa:286077	FAM83H	FAM83H_S892	RGSPTTGFIEQK	-0.291	0.396	0.070	0.775	0.322	0.013
hsa:10160	FARP1	FARP1_S427	VSAGEPGSHPSPAPR	-0.043	0.379	0.108	0.176	0.258	0.009
hsa:26272	FBXO4	FBXO4_S12	SGTNSPPPPFSDWGR	0.371	0.520	0.577	0.035	0.565	0.033
hsa:115548	FCHO2	FCHO2_S403	NLSNEELTK	0.560	0.041	0.388	0.131	0.128	0.781
hsa:83706	FERMT3	FERMT3_S497	TGSGGPGNHPHGPDASAEGLNPYGLVAPR	-0.195	0.489	0.124	0.793	-0.736	0.023
hsa:121512	FGD4	FGD4_S392	SLEIISTAASHSNSAIR	-0.075	0.694	-0.425	0.047	-0.075	0.694
hsa:81608	FIP1L1	FIP1L1_T494_S500	ERDHSPTPSVFNSDEER	0.379	0.356	0.640	0.038	0.248	0.213
hsa:23307	FKBP15	FKBP15_S1024	KGDSEAEALSEIKDGSLPPELSCIPSHR	0.413	0.617	0.793	0.283	1.320	0.006
hsa:23307	FKBP15	FKBP15_S979	ERPESPMVPSEQVVEEAVPLPPQALTTSQDGHRR	-1.072	0.008	-0.447	0.096	-0.806	0.006
hsa:23307	FKBP15	FKBP15_S619	NNSLQTATENTQAR	0.683	0.009	0.531	0.160	0.579	0.046
hsa:23307	FKBP15	FKBP15_S1097	EVAPDGPLQESSTRLSLTSDPEEGDPLALGPESPGEPQPPQLK	-1.140	0.049	-0.997	0.079	-1.157	0.048
hsa:23307	FKBP15	FKBP15_S956	RPSQEQSASASSGQPQAPLNR	-0.247	0.080	0.432	0.014	0.251	0.066
hsa:201163	FLCN	FLCN_S62	AHSPAEGASVESSSPGPK	-0.787	0.023	-0.200	0.336	-0.077	0.564
hsa:2313	FLI1	FLI1_S39	ADMTASGSPDYGQPHK	-0.662	0.025	-0.378	0.127	-0.549	0.006
hsa:2313	FLI1	FLI1_S241	GAWGNNMNSGLNKSPPLGGAQTISK	0.113	0.992	0.461	0.061	0.581	0.030
-	FLJ45252	YJ005_S175	KKSMEELTVIQCTSQELPAQTGLLSQTGDVPLPAGR	-0.864	0.023	-0.291	0.327	-0.654	0.118
hsa:2316	FLNA	FLNA_T2336	RLTVSSLQESGLK	0.084	0.543	0.024	0.940	0.299	0.024
hsa:2316	FLNA	FLNA_S2180	IPEISIQDMTAQVTSPSGK	-0.103	0.640	-0.281	0.356	-0.493	0.045
hsa:2316	FLNA	FLNA_S2152_S2158	RAPSVANVGSHCDLSLK	0.053	0.485	0.352	0.047	0.385	0.063
hsa:752	FMNL1	FMNL1_S184	NKPLEQSVEDLSKGPPSSVPK	0.041	0.810	0.026	0.892	0.168	0.043
hsa:752	FMNL1	FMNL1_S1031	GEPPAPKSPPK	-0.033	0.706	-0.708	0.020	-0.327	0.189

hsa:23360	FNBP4	FNBP4_S116	ATGGLCLLGAYADSDDDDNDVSEK	1.691	0.070	2.317	0.035	1.430	0.129
hsa:22862	FNDC3A	FNDC3A_S203_S207_S213	MSSPPSSPQKCPSPINEHNGLIK	-0.224	0.324	-0.449	0.103	-0.964	0.001
hsa:221937	FOXK1	FOXK1_T247	SMVSPVPSPTGTISVPNSCPASPR	-0.429	0.051	-0.196	0.232	-0.257	0.013
hsa:221937	FOXK1	FOXK1_T436	SGGLQTPECLSR	-0.097	0.665	0.312	0.002	0.144	0.375
hsa:3607	FOXK2	FOXK2_S428	FAQSAPGSPLSSQPVLITVQR	1.041	0.109	1.715	0.006	0.613	0.090
hsa:10146	G3BP1	G3BP1_S149	YQDEVFGGFVTEPQEESEEEVEEPEER	-0.384	0.015	-0.144	0.380	-0.199	0.202
hsa:10146	G3BP1	G3BP1_S232	SSSPAPADIAQTVQEDLR	-0.126	0.505	-0.219	0.046	0.099	0.552
hsa:2597	GAPDH	GAPDH_T153	IISNASCTTNCLAPLAK	0.479	0.360	0.679	0.162	1.362	0.030
hsa:26130	GAPVD1	GAPVD1_T762	EVSSRPSTPGLSVVSGISATSEDIPNKIEDLR	-0.344	0.323	-0.535	0.125	-1.064	0.008
hsa:8522	GAS7	GAS7_S117	RYYVNTTTNETTWERPSSSPGIPASPGSHR	-1.585	0.019	-0.487	0.409	-1.552	0.027
hsa:8729	GBF1	GBF1_S347_S349	SQSASVESIPEVLEECTSPADHSDSASVHDMDYVNPR	-0.130	0.024	0.134	0.616	0.081	0.619
hsa:2665	GDI2	GDI2_S61	FKIPGSPPESMGR	0.276	0.021	0.261	0.076	0.259	0.386
hsa:23163	GGA3	GGA3_S159	TLIPSPPPRPK	-0.138	0.444	-0.193	0.038	-0.205	0.173
hsa:28964	GIT1	GIT1_S592	HGSGADSDYENTQSGDPLLGLEGK	-0.151	0.045	-0.169	0.516	-0.026	0.942
hsa:9815	GIT2	GIT2_S415	QKSLDSDLSDGPVTVQEFMEVK	-0.753	0.022	-0.154	0.513	-0.527	0.016
hsa:51291	GMIP	GMIP_S437	SLDSPTSSPGAGTR	0.076	0.534	0.053	0.809	0.358	0.013
hsa:2794	GNL1	GNL1_S51	REEQTDTSDGESVTHHIR	-0.664	0.141	-1.098	0.061	-1.054	0.027
hsa:9950	GOLGA5	GOLGA5_S116	KKSEPDDELLFDFLNSSQK	-1.212	0.019	-1.622	0.018	-2.413	0.009
hsa:55425	GPALPP1	GPALPP1_S105	KQDDSPPRPIIGPALPPGFIK	0.318	0.322	0.639	0.051	0.981	0.019
hsa:23131	GPATCH8	GPATCH8_S740	GPKPEPPGSGSPAPPRR	-0.375	0.082	-0.378	0.022	-0.250	0.226
hsa:23432	GPR161	GPR161_S356	TSRLFSISNR	0.155	0.580	0.078	0.815	0.486	0.049
hsa:285513	GPRIN3	GPRIN3_S214	VVSHSSSPVGGPEGER	-0.547	0.244	-0.759	0.048	-0.684	0.092
hsa:23151	GRAMD4	GRAMD4_S28	RDDFLDLAESPNASDTECSDEIPLKVPR	0.330	0.441	0.538	0.781	0.470	0.039
hsa:56850	GRIPAP1	GRIPAP1_S704	SLSSSPQAQPPRPAELSDEEVAELFQR	0.215	0.017	0.159	0.522	0.294	0.323
hsa:2969	GTF2I	GTF2I_S679	SPGSNSKVPEIEVTVEGPNNNNPQTSAVR	-0.383	0.159	-0.202	0.040	0.103	0.563
hsa:9567	GTPBP1	GTPBP1_S25	SAMDSPVPASMFAPEPSSPGAAR	-0.163	0.035	-0.019	0.887	0.172	0.092
hsa:3065	HDAC1	HDAC1_S393	MLPHAPGVQMQAIPEDAIPEESGDEDEDDPDKR	0.687	0.190	1.398	0.043	1.868	0.013
hsa:3066	HDAC2	HDAC2_S394	MLPHAPGVQMQAIPEDAVHEDSGDEDGEDPDKR	-1.127	0.906	0.332	0.596	2.807	0.014
hsa:9759	HDAC4	HDAC4_S265	SSPLLR	-0.351	0.044	-0.215	0.205	-0.022	0.883

hsa:84717	HDGFL2	HDGFRP2_S454	KRSEGFSMDR	-1.442	0.166	-2.585	0.036	-11.023	0.003
hsa:9931	HELZ	HELZ_S1317	SPESRPSVVYPSTK	-0.146	0.316	0.369	0.188	0.378	0.042
hsa:8925	HERC1	HERC1_S1428	RVSTDLPEGQDVYTAACNSVIHR	-0.216	0.017	-0.055	0.702	0.128	0.004
hsa:8925	HERC1	HERC1_T1429	RVSTDLPEGQDVYTAACNSVIHR	0.004	0.934	0.349	0.587	0.647	0.011
hsa:255809	HIDE1	HIDE1_S216_S217	KRPTSTSSSPETPEFSTFR	-0.333	0.122	-0.123	0.553	-0.512	0.039
hsa:255809	HIDE1	HIDE1_S213	KRPTSTSSSPETPEFSTFR	-0.582	0.018	-0.373	0.195	-0.380	0.199
hsa:3008	HIST1H1E	HIST1H1E_S36	KASGPPVSELITK	-0.275	0.303	-0.527	0.080	-0.657	0.015
hsa:8359	HIST1H4A	HIST1H4A_S48	RISGLIYEETR	0.036	0.962	0.455	0.129	0.484	0.035
hsa:3105	HLA-A	HLA-A_S356_S359	GGSYTQAASSDSAQGSDVSLTACKV	0.242	0.306	0.400	0.032	0.424	0.313
hsa:3150	HMGN1	HMGN1_S7	KVSSAEGAAK	1.895	0.108	1.926	0.059	2.920	0.033
hsa:23526	HMHA1	HMHA1_S23	AGSPSPQPSGELPR	-0.019	0.893	-0.065	0.772	0.246	0.006
hsa:23526	HMHA1	HMHA1_S569	DQEPDVHYDFEPHVSANAWSPVMR	-0.452	0.083	-0.028	0.798	-0.610	0.016
hsa:23526	HMHA1	HMHA1_S578	KSSFNVSDVARPEAAGSPPEEGGCTEGTPAK	0.019	0.850	0.381	0.239	0.468	0.025
hsa:3181	HNRNPA2B1	HNRNPA2B1_S259	GFGDGYNGYGGGPGGGNFGGSPGYGGGR	0.398	0.273	0.556	0.034	0.697	0.048
hsa:3184	HNRNPD	HNRNPD_S83	NEEDEGHSNSSPR	1.855	0.052	2.274	0.041	2.390	0.093
hsa:3187	HNRNPH1	HNRNPH1_S63	EGRPSGEAFVELESEDEVK	0.214	0.022	-0.371	0.251	-0.388	0.090
hsa:3188	HNRNPH2	HNRNPH2_S104	HTGPNSPDTANDGFVR	0.117	0.374	0.034	0.979	0.331	0.039
hsa:3190	HNRNPK	HNRNPK_M283_S284	DYDDMSPR	0.920	0.032	1.171	0.027	1.051	0.112
hsa:3190	HNRNPK	HNRNPK_S379	GSYGDLGGPIITTQVTIPK	0.418	0.087	0.357	0.036	0.272	0.188
hsa:3190	HNRNPK	HNRNPK_S284	RDYDDMSPR	0.956	0.048	0.748	0.086	0.357	0.261
hsa:4670	HNRNPM	HNRNPM_S468	MGPLGLDHMASSIER	0.376	0.187	0.409	0.043	0.427	0.005
hsa:4670	HNRNPM	HNRNPM_S633_S637	GNFGGSFAGSFGGAGGHAPGVAR	0.615	0.018	1.191	0.005	0.539	0.023
hsa:4670	HNRNPM	HNRNPM_S528	MGLSMER	0.419	0.394	0.411	0.319	0.628	0.034
hsa:3192	HNRNPU	HNRNPU_S271	SPQPPVEEEDEHFDDTVVCLDTYNCDLHFK	-0.371	0.007	-0.445	0.203	0.037	0.896
hsa:50809	HP1BP3	HP1BP3_S156	QTPMASSPRPK	-0.675	0.041	-0.453	0.163	-0.327	0.185
hsa:3297	HSF1	HSF1_S303	VKEEPPSPPQSPR	0.103	0.663	-0.036	0.732	0.296	0.048
hsa:3320	HSP90AA1	HSP90AA1_S263	ESEDKPEIEDVGSDEEEEK	0.303	0.039	0.195	0.542	-0.491	0.192
hsa:3329	HSPD1	HSPD1_S253	ISSIQSIVPALEIANAHR	-2.753	0.081	-2.421	0.101	-3.405	0.039
hsa:10808	HSPH1	HSPH1_S809	IESPKLER	0.185	0.318	0.184	0.169	0.395	0.038

hsa:27336	HTATSF1	HTATSF1_S387	SDSVSASER	2.415	0.214	2.505	0.024	3.132	0.026
hsa:27336	HTATSF1	HTATSF1_S616	VLDEEGSER	3.265	0.021	3.620	0.040	3.932	0.096
hsa:3383	ICAM1	ICAM1_T530	GTPMKPNTQATPP	0.047	0.760	0.303	0.060	0.487	0.008
hsa:9641	IKBKE	IKBKE_S664	GAQASPPPIAPYPSPTR	-0.069	0.773	0.235	0.408	0.634	0.028
hsa:3609	ILF3	ILF3_S482_T486	DSSKGEDSAEETEAKPAVVAPAPVVEAVSTPSAAFPSDATAEQGPILTK	1.222	0.112	1.252	0.007	0.600	0.304
hsa:3614	IMPDH1	IMPDH1_S160	LVGIVTSR	0.092	0.522	0.303	0.095	0.206	0.038
hsa:3615	IMPDH2	IMPDH2_S160	LVGIISSR	0.092	0.522	0.303	0.095	0.206	0.038
hsa:83444	INO80B	INO80B_S130	AWLDEDSNLSPSPLR	0.060	0.755	-0.217	0.431	-1.007	0.002
hsa:83444	INO80B	INO80B_S351	LGGPEGPGSPLLAT	-0.275	0.017	-0.240	0.234	-0.129	0.329
hsa:3635	INPP5D	INPP5D_S243	LFDQQLSPGLRPRPQVPGEANPINMVSK	1.024	0.010	0.620	0.131	0.560	0.346
hsa:65123	INTS3	INTS3_S502	EKFPEFCSSPSPPVEVK	0.436	0.031	-0.251	0.363	0.089	0.657
hsa:26145	IRF2BP1	IRF2BP1_S436	NVAEALGHSPK	-0.378	0.036	-0.437	0.055	-0.579	0.173
hsa:26145	IRF2BP1	IRF2BP1_S453	AGGASPAASSTAQPPTQHR	-0.302	0.189	-0.406	0.046	-0.251	0.260
hsa:359948	IRF2BP2	IRF2BP2_S175	LEEPPELNRQSPNPR	-0.034	0.510	0.056	0.540	0.150	0.013
hsa:359948	IRF2BP2	IRF2BP2_S360	RKPSPEPEGEVGPPK	-0.342	0.001	-0.160	0.629	-0.130	0.549
hsa:64207	IRF2BPL	IRF2BPL_S658_S662	NSSSPVSPASVPGQR	-0.048	0.917	-0.290	0.015	-0.473	0.210
hsa:8660	IRS2	IRS2_S915	SPGEYINIDFGEPGAR	-0.417	0.097	-0.179	0.278	-0.521	0.040
hsa:3689	ITGB2	ITGB2_T758	SATTTVMNPK	-0.220	0.487	-1.139	0.095	-1.866	0.045
hsa:3689	ITGB2	*ITGB2 seq: 578 - 586 no mod*	TTEGCLNPR	1.103	0.020	-0.239	0.773	0.538	0.477
hsa:3707	ITPKB	ITPKB_T33	SGGGPGPSGSETPPPPRR	0.023	0.826	0.045	0.658	0.226	0.002
hsa:6453	ITSN1	ITSN1_S315	SGSGISVISSTSVDQR	-0.040	0.631	0.256	0.347	0.371	0.029
hsa:6453	ITSN1	ITSN1_S904	SAFTPATATGSSPSPVLGQGEK	-0.108	0.576	0.189	0.344	0.310	0.033
hsa:6453	ITSN1	ITSN1_T894	SAFTPATATGSSPSPVLGQGEKVEGLQAQALYPWR	0.162	0.035	0.090	0.566	0.073	0.479
hsa:55677	IWS1	IWS1_S438_S440	TIASDSEEEAGKELSDKK	0.408	0.034	0.429	0.336	-0.307	0.446
hsa:51155	JPT1	HN1_S88	RNSSEASSGDFLDLK	-0.213	0.018	0.230	0.061	0.155	0.301
hsa:3726	JUNB	JUNB_T255	DATPPVSPINMEDQER	-0.436	0.143	-0.509	0.112	-0.458	0.043
hsa:284058	KANSL1	KANSL1_S991_S994	SPISPELHSAPLTPVAR	0.406	0.081	0.696	0.046	0.184	0.404
hsa:23028	KDM1A	KDM1A_S69	EPPRASPPGGLAEPPGSAGPQAGPTVVPGSATPMETGIAETPEGR	0.050	0.663	0.182	0.008	0.194	0.478
hsa:221656	KDM1B	KDM1B_S247	AAATGNASPGKLEHSK	-0.223	0.372	-0.409	0.075	-0.706	0.007

hsa:22992	KDM2A	KDM2A_T713_S718	SCDEPLTPPPHSPTSMLQLIHDPVSPR	-0.201	0.252	0.260	0.147	0.218	0.015
hsa:10657	KHDRBS1	KHDRBS1_S20	SGSMDPSGAHPSVR	-0.397	0.313	-0.886	0.097	-0.781	0.038
hsa:23351	KHNYN	KHNYN_S359	VPSPPPAPEPPWHCGDR	0.651	0.040	0.211	0.583	0.213	0.842
hsa:9764	KIAA0513	KIAA0513_S74	SSSNESFSSNQSTESTQDEETLALR	-0.171	0.545	0.067	0.801	0.209	0.047
hsa:23313	KIAA0930	KIAA0930_S324	KSHSANDSEEFFREDDGGADLHNATNLR	-2.108	0.047	-1.948	0.072	-2.407	0.033
hsa:23313	KIAA0930	KIAA0930_S304	NNRPAFFSPSLKR	0.361	0.076	0.291	0.017	0.239	0.367
hsa:23313	KIAA0930	KIAA0930_S267	VSTGDTSPCGTEEDSSPASPMHER	-0.355	0.273	-0.248	0.048	0.073	0.861
hsa:57456	KIAA1143	KIAA1143_S50	IQPQPPDEDGDHSDKEDEQPQVVVLK	1.265	0.326	1.755	0.016	1.678	0.018
hsa:57536	KIAA1328	KIAA1328_S521	YETSLLDLVQSLSPNSAPKPQR	0.166	0.192	0.437	0.030	0.447	0.117
hsa:25758	KIAA1549L	KIAA1549L_S1653	TGPVAVASLR	-0.270	0.049	-0.280	0.203	-0.217	0.359
hsa:57498	KIDINS220	KIDINS220_S1555	VPKSPEHSAEPIR	1.355	0.036	1.239	0.050	1.840	0.005
hsa:57498	KIDINS220	KIDINS220_S1411	SSPHSTYYMGQSSSGGSIHSNLEQEK	0.197	0.395	0.625	0.078	0.337	0.037
hsa:23303	KIF13B	KIF13B_S1410	GRWESQQDVSQTTVSR	0.491	0.010	0.393	0.049	0.371	0.115
hsa:23095	KIF1B	KIF1B_S1487	GDSLILEHQWELEKLELLHEVEK	-2.921	0.009	-1.179	0.108	-2.189	0.010
hsa:10749	KIF1C	KIF1C_S915	MPSARPPSPPLSSWER	0.379	0.009	0.362	0.111	0.416	0.250
hsa:3831	KLC1	KLC1_S521	SRESLNVDVVK	-0.387	0.029	-0.181	0.060	-0.307	0.050
hsa:3831	KLC1	KLC1_S524	SRESLNVDVVK	-0.415	0.044	-0.302	0.103	-0.323	0.233
hsa:89953	KLC4	KLC4_S590	AASLNYLNQPSAAPLQVSR	0.403	0.102	0.536	0.044	0.432	0.189
hsa:9757	KMT2B	KMT2B_S861	ERPSGPESPVQGPR	-0.492	0.049	-0.173	0.521	0.020	0.907
-	KR87P	KR87P_T232	LAWTLR	0.508	0.338	1.221	0.050	1.342	0.036
hsa:65095	KRI1	KRI1_S171	AFVEDSEDEDGAGEGGSSLLQK	0.352	0.507	-0.206	0.709	-1.320	0.022
hsa:3895	KTN1	KTN1_S75	KEIQNGNLHESDSESVPR	-0.900	0.389	-2.053	0.158	-3.074	0.036
hsa:51056	LAP3	LAP3_S194	GVLFASGQNLAR	0.028	0.903	-0.146	0.492	-0.356	0.006
hsa:23367	LARP1	LARP1_T526	AVTPVPTKTEEVSNLK	-0.322	0.252	-0.253	0.149	-0.647	0.011
hsa:23367	LARP1	LARP1_S90	ESPRPLQLPGAEGPAISDGEEGGGEPGAGGGAAGAAGAGR	-0.211	0.225	0.084	0.820	-0.217	0.024
hsa:23367	LARP1	LARP1_S143	NALPPVLTTVNGQSPPEHSAPAK	-0.072	0.619	0.170	0.004	0.206	0.201
hsa:55132	LARP1B	LARP1B_S343	IGSPLSPK	-0.234	0.664	-0.260	0.018	-0.394	0.023
hsa:55132	LARP1B	LARP1B_S340	IGSPLSPK	-0.321	0.220	-0.556	0.018	-0.231	0.310
hsa:3927	LASP1	LASP1_M131_S146	MGPSGGEGMEPERRDSQDGSSYR	0.106	0.064	0.248	0.017	0.357	0.021

hsa:3927	LASP1	LASP1_S146	RDSQDGSSYR	1.505	0.047	1.459	0.071	1.585	0.074
hsa:27040	LAT	LAT_T39	LPGSYDSTSSDSLYPR	0.164	0.100	0.413	0.143	0.480	0.006
hsa:23592	LEMD3	LEMD3_S144	VLLGFSSDESDVEASPR	-0.479	0.268	-0.705	0.140	-1.118	0.007
hsa:3980	LIG3	LIG3_S242	FSGFSAKPNNSGEAPSSPTPK	0.518	0.025	0.631	0.187	0.935	0.036
hsa:11025	LILRB3	LILRB3_S503	SSPAADVQEENLYAAVK	-0.028	0.799	-0.241	0.288	-0.275	0.025
hsa:11025	LILRB3	LILRB3_S522	DTQSEDRVELDSQSPHDEDPQAVTYAPVK	-0.144	0.562	0.149	0.263	-0.067	0.035
hsa:11006	LILRB4	LILRB4_S319	SSPAADVQGENFCAAVK	-0.069	0.627	0.356	0.107	0.338	0.003
hsa:11006	LILRB4	LILRB4_T336	NTQPEDGVEMDTRQSPHDEDPQAVTYAK	-0.868	0.223	-0.744	0.278	-1.372	0.049
hsa:51474	LIMA1	LIMA1_S692_S698	EGHSLEMENENLVENGADSDEDDNSFLKQQSPQEPK	-0.419	0.042	-0.021	0.955	0.250	0.619
hsa:3984	LIMK1	LIMK1_S298_S310	SCSIDRSPGAGSLGSPASQR	-0.705	0.075	-0.856	0.016	-1.068	0.001
hsa:4000	LMNA	LMNA_S616	ASASGSGAQVGGPISSGSSASSVTVTR	0.123	0.650	0.612	0.079	0.582	0.012
hsa:4000	LMNA	LMNA_S414	ASSHSSQTQGGGSVTK	0.939	0.425	1.228	0.204	1.563	0.020
hsa:4000	LMNA	LMNA_S407	ASSHSSQTQGGGSVTK	0.961	0.410	1.116	0.223	1.572	0.023
hsa:4000	LMNA	LMNA_S463	SNEDQSMGNWQIK	0.211	0.545	0.580	0.176	0.747	0.025
hsa:4000	LMNA	LMNA_S390_S392	LRLSPSPTSQR	-0.016	0.933	0.061	0.743	0.259	0.034
hsa:4000	LMNA	LMNA_S403	GRASSHSSQTQGGGSVTK	1.377	0.099	1.581	0.521	1.556	0.041
hsa:23143	LRCH1	LRCH1_S532_S536	ENSPAVSPTTNSTAPFGLKPR	0.843	0.167	1.112	0.149	1.434	0.026
hsa:84859	LRCH3	LRCH3_S415	RISHEGSPVKPVAIR	0.385	0.641	0.836	0.047	0.895	0.025
hsa:145581	LRFN5	LRFN5_T53	RTVELR	0.473	0.766	2.448	0.021	4.282	0.000
hsa:57470	LRRC47	LRRC47_S520	KYTLENKEEGSLSDTEADAVSGQLPDPTTNPSAGK	0.055	0.732	0.220	0.016	0.270	0.252
hsa:9208	LRRFIP1	LRRFIP1_S768	KALDSNSLENDDLSAPGREPGHFNPESR	0.004	0.962	0.094	0.570	0.112	0.003
hsa:164312	LRRN4	LRRN4_S332	RTVLSR	1.233	0.160	1.539	0.037	1.959	0.030
hsa:222229	LRWD1	LRWD1_S251	ACASPSAQVEGSPVAGSDGSQPAVK	0.011	0.906	0.444	0.024	0.437	0.183
hsa:26065	LSM14A	LSM14A_S192	SPTMEQAVQTASAHLPAPAAVGR	0.287	0.049	0.040	0.920	-0.002	0.835
hsa:4046	LSP1	LSP1_S111	SPEGEQEDRPGLHAYEK	0.400	0.045	0.345	0.303	0.391	0.221
hsa:4046	LSP1	LSP1_T175_S177_T196	TPSPLVLEGTIEQSSPPLSPTTK	-1.040	0.748	-0.424	0.043	-0.107	0.987
hsa:4067	LYN	LYN_S166	GSFSLSVR	-1.703	0.107	-2.528	0.038	-1.983	0.082
hsa:1130	LYST	LYST_S2627	RMSQENPSQATETELAQR	0.048	0.915	0.172	0.724	0.853	0.018
hsa:23499	MACF1	MACF1_S3927	QGSFSEDVISHK	0.093	0.724	-0.016	0.912	0.347	0.023

hsa:23499	MACF1	MACF1_S7279	RPTPTFHSSR	-0.282	0.282	-0.538	0.115	-0.620	0.025
hsa:23499	MACF1	MACF1_S5588	EVEEELATSGGQSPTGEQIPQFQQR	-0.546	0.036	0.127	0.169	0.145	0.290
hsa:55201	MAP1S	MAP1S_S582	KAPSTSHSGFPPVANGPRSPPSLR	-0.946	0.323	-1.223	0.238	-3.944	0.005
hsa:55201	MAP1S	MAP1S_T638_S640	ALELPLAASSIPRPRTPSPESHR	0.119	0.833	0.484	0.035	0.120	0.871
hsa:5609	MAP2K7	MAP2K7_S55	SPSSESSPQHPTPPARPR	-0.034	0.890	0.363	0.034	0.330	0.328
hsa:4296	MAP3K11	MAP3K11_S507	ITVQASPGLDR	-0.229	0.119	-0.232	0.265	-0.554	0.002
hsa:10746	MAP3K2	MAP3K2_S164	DRSSPPPGYIPDELHQVAR	0.061	0.663	0.268	0.014	0.299	0.287
hsa:10746	MAP3K2	MAP3K2_S331	RRGSDIDNPTLTVMDISPPSR	0.204	0.007	0.819	0.175	0.784	0.302
hsa:4215	MAP3K3	MAP3K3_S234	SADSPSFR	3.066	0.147	2.733	0.135	3.833	0.008
hsa:4215	MAP3K3	MAP3K3_S176	SSPPPGYVPER	0.042	0.879	0.439	0.115	0.578	0.038
hsa:4134	MAP4	MAP4_S99	KKPCSETSQIEDTPSSKPTLLANGGHGVEGSDTTGSPTEFLEEK	2.876	0.039	3.319	0.025	3.377	0.023
hsa:9053	MAP7	MAP7_S202	LSSSSATLLNSPDR	0.738	0.042	0.224	0.838	0.164	0.688
hsa:55700	MAP7D1	MAP7D1_S399	RKPNAGGSPAPVR	-0.663	0.107	-0.461	0.316	-2.768	0.012
hsa:55700	MAP7D1	MAP7D1_S274	SSATLWNSPSR	-1.512	0.096	-1.861	0.046	-1.496	0.093
hsa:55700	MAP7D1	MAP7D1_S539	ESAAPASPAPSPAPSPTPAPPQK	-0.692	0.095	-0.628	0.030	-0.327	0.286
hsa:4141	MARS	MARS_S825	TSPKPAVVETVTTAKPQQIQALMDEVTK	-0.034	0.867	-0.502	0.321	-1.364	0.023
hsa:23139	MAST2	MAST2_S913	RLSVSESSHTESDSSPPMTVR	-0.216	0.027	-0.281	0.005	-0.161	0.053
hsa:23139	MAST2	MAST2_S900	SWVIGSPEILR	0.316	0.005	0.085	0.563	-0.058	0.803
hsa:9782	MATR3	MATR3_S206	GPSLNPVLDYDHGSR	1.066	0.275	0.810	0.172	1.174	0.050
hsa:57506	MAVS	MAVS_S165	EKEPSYPMPVQETQAPESPGENSEQALQTLSPR	-0.436	0.374	-0.153	0.860	-1.003	0.046
hsa:4171	MCM2	MCM2_S27	GNDPLTSSPGR	0.138	0.246	0.173	0.666	0.457	0.000
hsa:4171	MCM2	MCM2_S139	GLLYDSDEEDEERPAR	0.412	0.020	0.441	0.030	0.509	0.034
hsa:84331	MCRIP2	FAM195A_T86	APSTSPSFEGTQETYTVAHEENVR	-0.405	0.519	0.042	0.028	-0.100	0.889
hsa:4204	MECP2	MECP2_S80	HEPVQPSAHHSAEPAEAGKAETSEGSGSAPAVPEASASPK	-1.972	0.012	-1.736	0.021	-2.228	0.006
hsa:5469	MED1	MED1_T1051	SQTPPGVATPPIPK	-0.168	0.121	0.213	0.399	0.152	0.023
hsa:219541	MED19	MED19_S226	NRHSPDHPGMGSSQASSSSSLR	-1.957	0.004	-2.121	0.039	-5.180	0.000
hsa:4205	MEF2A	MEF2A_S496	ESPSVKR	0.776	0.088	0.652	0.102	0.885	0.038
hsa:150365	MEI1	MEI1_T130	LEQTIR	0.473	0.414	0.566	0.411	0.917	0.048
hsa:56257	MEPCE	MEPCE_S390	GRGSWGGR	2.405	0.020	1.181	0.541	3.854	0.013

hsa:4236	MFAP1	MFAP1_S53	RPDYAPMESSDEEDEEFQFIKK	-0.333	0.032	-0.169	0.276	0.012	0.949
hsa:84179	MFSD7	MFSD7_S507	GPGSPHPACHR	-0.649	0.013	-1.137	0.008	-1.223	0.004
hsa:64780	MICAL1	MICAL1_M614_S617	SMAHSPGPVSQASPGTSSAVLFLSK	-0.253	0.049	-0.004	0.992	0.251	0.353
hsa:64780	MICAL1	MICAL1_T475	AVTPNQVR	-0.591	0.015	-0.092	0.590	0.055	0.742
hsa:85377	MICALL1	MICALL1_S484_S486	APSASPLALHASR	-0.042	0.809	0.169	0.731	-0.799	0.009
hsa:85377	MICALL1	MICALL1_S471	SLHPWYGITPTSSPK	0.332	0.492	0.368	0.249	0.364	0.037
hsa:79778	MICALL2	MICALL2_S649	TPRPASPGPSLPAR	-0.281	0.343	-0.537	0.155	-0.526	0.033
hsa:50488	MINK1	MINK1_S763	SDSVLPASHGHLPQAGSLER	0.381	0.350	0.419	0.114	0.397	0.034
hsa:50488	MINK1	MINK1_S918	NLLHADSNGYTNLPDVVQPSHSPTENSK	0.084	0.765	0.372	0.030	0.842	0.062
hsa:54468	MIOS	MIOS_S766	GFSQYGVSGSPTK	0.081	0.697	0.119	0.652	0.407	0.027
hsa:79169	MMTAG2	MMTAG2_S220	RPAEATSSPTSPERPR	-0.410	0.082	-0.506	0.031	-0.526	0.127
hsa:84315	MON1A	MON1A_S31	AESPTPGMAQGMEPGAGQEGAMFVHAR	0.119	0.168	0.408	0.026	0.375	0.015
hsa:22880	MORC2	MORC2_S615	SPPLPAVIR	0.426	0.012	0.209	0.306	0.203	0.197
hsa:10199	MPHOSPH10	MPHOSPH10_S163_S167	KSPVFSDEDSDLDFDISKLEQQSK	-0.505	0.030	-0.009	0.898	0.155	0.366
hsa:54737	MPHOSPH8	MPHOSPH8_S51	GAEAFGDSEEDGEDVFEVEK	-0.393	0.407	-1.626	0.037	-2.536	0.003
hsa:54737	MPHOSPH8	MPHOSPH8_S403	GLWSTDSAEEDKETK	-0.015	0.954	-0.247	0.029	-0.438	0.070
hsa:4361	MRE11A	MRE11A_S688_S689	GVDFESSEDDDDDPFMNTSSLRR	-0.146	0.509	0.002	0.991	0.267	0.021
hsa:92259	MRPS36	MRPS36_S90	LVSQEEMEFIQR	-0.262	0.048	0.138	0.043	0.291	0.124
hsa:10943	MSL3	MSL3_S400	SSSPIPLTPSK	0.590	0.507	1.133	0.020	1.196	0.009
hsa:4478	MSN	MSN_S576	QRIDEFESM	0.304	0.138	0.258	0.357	0.573	0.037
hsa:9219	MTA2	MTA2_S435	GHLSRPEAQSLSPYTTSANR	0.535	0.301	0.429	0.151	0.814	0.019
hsa:4597	MVD	MVD_S96	NSRDGDPLPSSLSCK	-0.246	0.463	-0.300	0.041	-0.187	0.274
hsa:9961	MVP	MVP_S445	SLQPLAPR	-0.137	0.253	0.071	0.512	0.229	0.018
hsa:9961	MVP	MVP_S867	VASGPSPGEGISPQSAQAPQAPGDNHVVPVLR	0.334	0.377	0.572	0.040	0.785	0.037
hsa:10514	MYBBP1A	MYBBP1A_S1267	NQKPSQVNGAPGSPTEPAGQK	0.296	0.956	0.139	0.717	0.640	0.045
hsa:23077	MYCBP2	MYCBP2_S2833	SKSDSYTLDPDTLR	-0.185	0.264	-0.348	0.001	-0.322	0.024
hsa:23077	MYCBP2	MYCBP2_S2643	HEDEQALLDQNSQTPPPSPFSVQAFNK	-0.223	0.480	-0.200	0.331	0.059	0.031
hsa:4627	MYH9	MYH9_S1943	GAGDGSDEEVDGK	0.564	0.030	0.967	0.133	0.501	0.105
hsa:399687	MYO18A	MYO18A_S2007	SSSPTSYWK	0.480	0.029	0.579	0.051	0.512	0.013

hsa:399687	MYO18A	MYO18A_S2020	SLAPDRSDDEHDPLDNTSRPR	-0.307	0.311	-0.230	0.426	-0.644	0.043
hsa:399687	MYO18A	MYO18A_S102	GSVILDSGHLSTASSSDDLKGEEGSFR	0.218	0.326	0.024	0.762	0.324	0.044
hsa:4643	MYO1E	MYO1E_S736	RNSINR	0.580	0.281	1.390	0.042	0.342	0.480
hsa:4644	MYO5A	MYO5A_S600	AISPTSATSSGR	-0.731	0.210	-1.293	0.036	-1.656	0.008
hsa:4650	MYO9B	MYO9B_S2141	RYSDPPTYCLPPASGQTNG	0.040	0.454	0.204	0.096	0.221	0.007
hsa:4650	MYO9B	MYO9B_S1354	RTSFSTSDVSK	0.216	0.210	0.199	0.473	0.428	0.023
hsa:4650	MYO9B	MYO9B_S717	AAGMSSPGAQSHPEELPR	-0.210	0.441	-0.190	0.471	-0.681	0.044
hsa:4650	MYO9B	MYO9B_S1992	GSDEENLDSETSASTESLLEER	1.425	0.039	1.047	0.093	1.264	0.059
hsa:4650	MYO9B	MYO9B_S1114	SPLEHSSPEKEAPSPEK	-0.372	0.257	-0.701	0.028	-0.407	0.182
hsa:114803	MYSM1	MYSM1_S218	IEKLSDDEEVDITDEVDELSSQTPQK	-0.331	0.034	-0.109	0.740	-0.081	0.640
hsa:4665	NAB2	NAB2_S171	SPLELGEKLSPLPGGPGAGDPR	-0.953	0.302	0.865	0.081	1.270	0.039
hsa:4665	NAB2	NAB2_S159_S162	SFSPKSPLELGEK	1.171	0.041	0.828	0.017	0.606	0.049
hsa:4666	NACA	NACA_S2029	VQGEAVSNIQENTQTPTVQEESEEEEVDETGVEVK	-0.541	0.160	-0.351	0.125	-0.417	0.050
hsa:4676	NAP1L4	NAP1L4_S125	EFITGDVEPTDAESEWHSENEEEEKLAGDMK	-0.120	0.872	0.747	0.025	0.803	0.032
hsa:89796	NAV1	NAV1_S1182	QNSSDSISSLNSITSHSSIGSSK	0.140	0.346	0.352	0.094	0.286	0.002
hsa:64151	NCAPG	NCAPG_S674	TLHCEGTEINSDDEQESKEVEETATAK	-0.426	0.399	-0.255	0.008	-0.555	0.001
hsa:4686	NCBP1	NCBP1_S22	KTSDANETEDHLESLICK	-0.183	0.646	0.313	0.030	0.482	0.026
hsa:654817	NCF1C	NCF1C_S349	QARPGPQSPGSPLEEER	-0.492	0.031	-0.292	0.088	-0.154	0.236
hsa:4690	NCK1	NCK1_S89	RKPSVPDSASPADDSFVDPGER	-0.157	0.339	0.378	0.093	0.432	0.015
hsa:4690	NCK1	NCK1_S85	RKPSVPDSASPADDSFVDPGER	0.108	0.321	0.649	0.041	0.546	0.282
hsa:57701	NCKAP5L	NCKAP5L_S436	LQIGPPSPGEAQGPLLPSPAR	1.539	0.055	1.824	0.030	0.933	0.501
hsa:4691	NCL	NCL_S67	KVVVSPTK	-0.107	0.468	-0.425	0.038	-0.046	0.602
hsa:9611	NCOR1	NCOR1_S1472	AQLSPGIYDDTSAR	-0.021	0.936	0.173	0.147	0.401	0.002
hsa:10397	NDRG1	NDRG1_S362	SRSHTSEGAHLDITPNSGAAGNSAGPK	-2.108	0.083	-1.555	0.118	-4.590	0.001
hsa:91754	NEK9	NEK9_S869	VASEAPLEHKPQVEASSPR	-0.553	0.050	-0.615	0.066	-0.670	0.128
hsa:7469	NELFA	NELFA_S363	EASRPPEEPSAPSPTLPAQFK	0.103	0.320	0.380	0.001	0.287	0.080
hsa:7936	NELFE	NELFE_S179	SGAHSSASPPR	0.587	0.037	0.696	0.058	0.748	0.021
hsa:9147	NEMF	NEMF_S417	NPYLLSEEEDDDVDGDVNVEKNETEPPK	-1.501	0.020	-0.337	0.461	-0.751	0.195
hsa:10763	NES	NES_S768	SLGEQDQMTLRPPEKVDLEPLK	-0.885	0.036	-0.116	0.675	-0.268	0.459

hsa:4763	NF1	NF1_S2543	SFDHLISDTK	-0.078	0.645	-0.439	0.041	-0.290	0.204
hsa:4798	NFRKB	NFRKB_S298	KGSLAALYDLAVLK	-0.405	0.097	-0.633	0.012	-0.390	0.086
hsa:4830	NME1	NME1_T79	VMLGETNPADSKPGTIR	0.101	0.033	0.046	0.891	0.115	0.698
-	NME2P1	NME2P1_T79	VMLGETNPADSKPGTIR	-0.299	0.364	0.606	0.037	0.530	0.044
hsa:26155	NOC2L	NOC2L_S49	EAARSPDKPGGSPSASR	-0.267	0.022	-0.119	0.851	0.369	0.336
hsa:9221	NOLC1	NOLC1_S538	GKGSPRPQAPK	-0.323	0.257	-1.364	0.127	-4.910	0.001
hsa:10528	NOP56	NOP56_S520	EELMSSDLEETAGSTSIPK	0.389	0.361	0.304	0.126	0.627	0.017
hsa:51602	NOP58	NOP58_S502	HIKEEPLSEEEPCTSTAIASPEK	-0.814	0.008	-1.026	0.002	-1.057	0.008
hsa:4869	NPM1	NPM1_S70	TVSLGAGAKDELHIVEAEAMNYEGSPIK	0.142	0.671	-0.277	0.443	-1.965	0.002
hsa:7182	NR2C2	NR2C2_S46	IQIVTAVDASGSPK	0.043	0.865	-0.663	0.023	-0.399	0.098
hsa:54888	NSUN2	NSUN2_S743_S751	AGEPNSPDAEEANSPDVTAGCDPAGVHPPR	-0.241	0.269	-0.126	0.380	-0.225	0.023
hsa:54888	NSUN2	NSUN2_S743	AGEPNSPDAEEANSPDVTAGCDPAGVHPPR	-0.157	0.290	0.190	0.022	0.300	0.364
hsa:4924	NUCB1	NUCB1_S369	AQRLSQETEALGR	-0.293	0.155	-0.032	0.706	0.167	0.016
hsa:64710	NUCKS1	NUCKS1_S19	VVDYSQFQESDDADEDYGR	-0.233	0.268	0.293	0.025	0.433	0.068
hsa:64710	NUCKS1	NUCKS1_S181	ATVTPSPVK	0.164	0.438	0.422	0.047	0.135	0.242
hsa:57532	NUFIP2	NUFIP2_S572	RTSPQVLGSILK	0.091	0.088	0.175	0.039	0.195	0.015
hsa:57532	NUFIP2	NUFIP2_S112	NLSSDEATNPISR	1.075	0.042	1.073	0.076	0.720	0.294
hsa:129401	NUP35	NUP35_T273	TLGTPTQPGSTPR	0.053	0.959	1.076	0.031	1.050	0.094
hsa:4927	NUP88	NUP88_T37	NQSPTEAEKPASSSLPSSPPPQLLTR	0.378	0.030	0.433	0.026	0.191	0.327
hsa:4928	NUP98	NUP98_S1060	FTSGAFLSPSVSVQECR	0.573	0.057	0.777	0.043	0.490	0.166
hsa:54940	OCIAD1	OCIAD1_S123	RSSPPGHYYQK	-0.192	0.303	-0.398	0.091	-0.529	0.017
hsa:10133	OPTN	OPTN_S528	TSDSDQQAYLVQR	0.310	0.149	0.625	0.087	0.632	0.003
hsa:5007	OSBP	OSBP_S190_S193	MLAESDESGDEESVSQTDKTELQNTLR	1.943	0.276	1.249	0.366	1.771	0.011
hsa:114885	OSBPL11	OSBPL11_S15	VSESEGKLEGQATAVTPNK	0.260	0.095	0.356	0.006	0.119	0.698
hsa:26031	OSBPL3	OSBPL3_S251	TYSAPAINAIQGGSFESPK	-0.047	0.753	-0.410	0.037	-0.877	0.079
hsa:8106	PABPN1	PABPN1_S150	QMNMSPPPGNAGPVIMSIEEK	-0.232	0.287	-0.080	0.540	0.112	0.015
hsa:55690	PACS1	PACS1_S529_S531	TNSSDSERSPDLGHSTQIPR	-0.374	0.005	0.019	0.944	-0.123	0.580
hsa:23241	PACS2	PACS2_S700_S702	VGIVEPSSATSGDSDDAAPSGSGTLSSTPPSASPAAK	-0.215	0.160	0.077	0.403	0.296	0.024
hsa:5062	PAK2	PAK2_S132	FYDSNTVK	-0.533	0.224	-0.498	0.037	-0.488	0.107

hsa:5062	PAK2	PAK2_S141	YLSFTPPEKDGFPSGTPALNAK	0.658	0.159	1.374	0.014	0.471	0.528
hsa:255967	PAN3	PAN3_S354	ITPHTSPAPR	-0.415	0.126	-0.794	0.002	-0.637	0.002
hsa:10914	PAPOLA	PAPOLA_S537	LTALNDSSLDLSMDSDNSMSVPSPTSATK	-0.146	0.492	0.126	0.034	0.261	0.294
hsa:64761	PARP12	PARP12_S261	DSSGSVSPNTLSQEEGDQICLYHIR	-0.317	0.159	0.172	0.426	0.377	0.043
hsa:51260	PBDC1	PBDC1_S197	GADSGEEKEEGINREDK	0.291	0.010	0.082	0.894	-0.532	0.319
hsa:5091	PC	PC_T21	TSTAPAASPNVR	-0.248	0.026	0.351	0.199	0.154	0.490
hsa:5093	PCBP1	PCBP1_S173	QICLVMLETLSQSPQGR	1.217	0.737	2.611	0.041	2.417	0.067
hsa:5093	PCBP1	PCBP1_S190	VMTIPYQPMPASSPVICAGGQDR	0.475	0.493	0.466	0.038	-0.144	0.477
hsa:51585	PCF11	PCF11_S489	SRSPIIHSPK	-1.073	0.241	-3.574	0.006	-5.044	0.000
hsa:51585	PCF11	PCF11_S489_S494	SRSPIIHSPK	-0.046	0.650	0.120	0.099	0.123	0.013
hsa:51585	PCF11	PCF11_S777	MIFEGPNKLSPR	-0.525	0.022	-0.126	0.492	-0.190	0.264
hsa:5116	PCNT	PCNT_S2214	KSPVGMLDLSSWSSPEVLR	0.414	0.010	0.319	0.228	0.351	0.113
hsa:27344	PCSK1N	PCSK1N_M1_S4	MAGSPLLWGPR	0.201	0.307	0.238	0.147	0.086	0.030
hsa:11333	PDAP1	PDAP1_S60_S63	SLDSDESEDEEDDYQQKR	-0.359	0.938	2.581	0.041	0.366	0.742
hsa:64236	PDLIM2	PDLIM2_S87	SQATSPGQTNGDSSLEVLATR	0.082	0.831	-0.059	0.779	-0.624	0.047
hsa:64236	PDLIM2	PDLIM2_S134	AGSPFSPPPSSSSLTGEAAISR	0.601	0.238	1.126	0.020	0.391	0.303
hsa:10611	PDLIM5	PDLIM5_S313	ANNSQEPSPQLASSVASTR	1.254	0.035	0.880	0.925	0.424	0.763
hsa:23244	PDS5A	PDS5A_T1208	IISVTPVK	0.713	0.238	0.088	0.030	0.031	0.258
hsa:23047	PDS5B	PDS5B_S1166	METVSNASSSSNPSSPGR	-0.051	0.682	-0.421	0.049	0.079	0.908
hsa:8682	PEA15	PEA15_S116	DIIRQPSEEEIIK	0.149	0.327	-0.119	0.049	0.274	0.149
hsa:5824	PEX19	PEX19_S147	NATDLQNSSMSEEELTK	0.325	0.032	0.375	0.151	0.191	0.892
hsa:5216	PFN1	PFN1_T93	STGGAPTFNVTVTK	0.510	0.044	0.017	0.945	0.108	0.708
hsa:5223	PGAM1	PGAM1_S23	FSGWYDADLSPAGHEEAK	1.079	0.074	1.681	0.031	1.253	0.008
hsa:5223	PGAM1	PGAM1_S118_M126	SYDVPPPPMEPDHPFYSNISK	0.204	0.292	0.282	0.060	0.382	0.050
hsa:5230	PGK1	PGK1_S203	ALESPERPFLAILGGAK	0.300	0.031	-0.017	0.953	0.050	0.839
hsa:10857	PGRMC1	PGRMC1_S181	EGEEPTVYSDEEEPKDESAR	0.666	0.021	0.422	0.168	-0.015	0.970
hsa:10424	PGRMC2	PGRMC2_T211	LLKPGEEPSEYTDEEDTKDHNKQD	-0.512	0.045	-0.429	0.146	-0.035	0.687
hsa:5253	PHF2	PHF2_S899	KGSDDAPYSPTAR	0.153	0.854	-0.084	0.572	0.231	0.011
hsa:55023	PHIP	PHIP_S674	GSISSTSEVHSPPNVGLR	0.093	0.616	0.255	0.129	0.314	0.040

hsa:5257	PHKB	PHKB_S701	RQSSTPSAPELGQQPDVNISEWK	0.218	0.416	0.039	0.849	0.168	0.018
hsa:23187	PHLDB1	PHLDB1_S518_S520	SPSPTLGESLAPHK	-0.334	0.463	0.150	0.464	0.265	0.046
hsa:57661	PHRF1	PHRF1_S973	TVTCVTVVEPEAPPSPDVLQAATHR	-0.090	0.356	-0.331	0.036	-0.294	0.113
hsa:57661	PHRF1	PHRF1_S915	GAVAAEGASDTEREEPTESQGLAAR	0.168	0.034	0.341	0.161	-0.142	0.652
hsa:55361	PI4K2A	PI4K2A_S47	VAAAAGSGPSPPGSPGHDR	-0.073	0.829	-0.398	0.041	-0.490	0.065
hsa:5297	PI4KA	PI4KA_S172	SFNDFR	0.115	0.590	0.329	0.025	0.651	0.003
hsa:23533	PIK3R5	PIK3R5_S348	DSLLSTSSLASHDSTLSLASSQASGPALSR	0.700	0.574	0.923	0.251	1.885	0.030
hsa:9600	PITPNM1	PITPNM1_S593	RGSMNNELLSPEFGPVRDPLADGVEGLGR	0.724	0.141	1.065	0.043	0.721	0.236
hsa:5311	PKD2	PKD2_S832	RGSISSGVSYEEFQVLVR	-0.832	0.026	-0.748	0.144	-0.252	0.440
hsa:5315	PKM	PKM_T524	KGDVVIVLTGWRPGSGFTNTMR	-1.841	0.028	-1.206	0.058	-1.508	0.032
hsa:5585	PKN1	PKN1_S916	TDVSNFDEEFTGEAPTLSPPR	-0.677	0.020	-0.498	0.137	-0.451	0.145
hsa:5339	PLEC	PLEC_S4384_S4400	SSSVGSSSSYPISPAVSR	0.532	0.007	0.558	0.016	0.420	0.066
hsa:5339	PLEC	PLEC_S1444	VQSGSESVIQEYVDLR	0.709	0.035	0.621	0.079	0.237	0.314
hsa:5339	PLEC	PLEC_S4386_S4389	SSSVGSSSSYPISPAVSR	0.201	0.004	0.251	0.438	-0.234	0.407
hsa:9842	PLEKHM1	PLEKHM1_S432	LVVSSPTSPK	0.792	0.046	0.192	0.543	-0.024	0.981
hsa:84898	PLXDC2	PLXDC2_S506	GSGHPAYAEVEPVGEK	-0.072	0.657	0.244	0.421	0.373	0.048
hsa:10154	PLXNC1	PLXNC1_S978	KQSQQLELLESELR	0.670	0.215	0.927	0.026	0.984	0.105
hsa:5371	PML	PML_S530	SPVIGSEVFLPNSNHVASGAGEAEER	0.229	0.991	0.280	0.916	0.694	0.005
hsa:25957	PNISR	PNISR_S211	QRSPIALPVK	0.165	0.162	0.342	0.149	0.261	0.049
hsa:5411	PNN	PNN_S66	RGFSDSGGGPPAK	0.135	0.178	0.014	0.917	0.152	0.041
hsa:5424	POLD1	POLD1_T678	ETDPLR	1.136	0.414	1.116	0.021	0.489	0.441
hsa:100101267	POM121C	POM121C_S357	GLNSQSSDDHLNKR	-0.220	0.359	-0.637	0.045	-0.543	0.173
hsa:5451	POU2F1	POU2F1_S448	INPPSSGGTSSSPIK	0.172	0.319	0.379	0.088	0.510	0.012
hsa:27068	PPA2	PPA2_S317	SLVESVSSSPNKESNEEEQVWHFLGK	0.224	0.626	0.778	0.044	0.571	0.228
hsa:8500	PPFIA1	PPFIA1_S708	RIPHSPAR	-0.761	0.357	-3.253	0.014	-4.680	0.001
hsa:8500	PPFIA1	PPFIA1_S763	GALHTVSHEDIR	-0.481	0.038	-0.432	0.051	-0.464	0.027
hsa:51535	PPHLN1	PPHLN1_S201	ERPVQSLKTSR	-0.578	0.011	-0.373	0.022	-0.403	0.135
hsa:9360	PPIG	PPIG_S415	NVSESPNRK	3.483	0.257	4.794	0.005	4.915	0.005
hsa:9360	PPIG	PPIG_T358	RSETPPHWR	0.360	0.276	0.720	0.006	0.763	0.038

hsa:23262	PPIP5K2	PPIP5K2_S38	HFFHHADEDDEEEDDSPPER	-2.220	0.060	-5.331	0.004	0.000	0.000
hsa:23262	PPIP5K2	PPIP5K2_S1006	SGEQITSSPVSPK	0.470	0.177	0.332	0.307	0.600	0.021
hsa:5514	PPP1R10	PPP1R10_S313	VLSPTAAKPSPFEGK	0.080	0.280	-0.040	0.686	0.153	0.047
hsa:6992	PPP1R11	PPP1R11_S77	AFGESSTESDEEEEEGCGHTHCVR	-0.007	0.979	0.132	0.022	-0.245	0.306
hsa:84988	PPP1R16A	PP16A_S333	QRSLLR	-1.108	0.391	-5.106	0.049	0.000	0.026
hsa:170954	PPP1R18	PPP1R18_S368	LLESPGVEAGEGEAEKEEAGAQGRPLR	-1.052	0.060	-0.094	0.657	-0.713	0.030
hsa:5504	PPP1R2	PPP1R2_T89	IDEPSTPYHSMMGDDEDACSDTEATEAMAPDILAR	-0.654	0.246	-1.241	0.034	-2.151	0.004
hsa:153743	PPP1R2B	PPP1R2P3_S122	IQEQESSGEEDSDLSPEER	0.417	0.969	2.834	0.158	2.114	0.023
hsa:5510	PPP1R7	PPP1R7_S24_S37	VESEESGDEEGKKHSSGIVADLSEQSLK	-0.481	0.028	0.001	0.987	-0.394	0.013
hsa:22870	PPP6R1	PPP6R1_S529_S531	NMVDLVNTHHLHSSSDDEDDRLK	-2.170	0.110	-1.487	0.224	-3.717	0.024
hsa:22870	PPP6R1	PPP6R1_S531	NMVDLVNTHHLHSSSDDEDDRLK	-1.579	0.043	-1.006	0.185	-1.430	0.082
hsa:55291	PPP6R3	PPP6R3_S617	IQQFDDGGSDEEDIWEEK	0.948	0.002	1.238	0.007	0.938	0.069
hsa:157285	PRAG1	SGK223_S653	IEEEEEVEQELLSHSWGR	-0.148	0.533	0.027	0.994	-0.657	0.022
hsa:5546	PRCC	PRCC_S157_S159	IAAPELHKGDSDSEEDEPTK	0.315	0.087	0.625	0.049	0.359	0.202
hsa:5565	PRKAB2	PRKAB2_S184	DLSSSPPGPYGQEMYAFR	0.692	0.025	0.859	0.030	1.095	0.055
hsa:5576	PRKAR2A	PRKAR2A_S99	RVSVCAETYNPDEEEEDTDPR	0.216	0.021	0.362	0.418	-0.051	0.869
hsa:5580	PRKCD	PRKCD_S304	RSDSASSEPVGIYQGFEK	0.120	0.260	0.400	0.102	0.530	0.040
hsa:5580	PRKCD	PRKCD_S645	ARLSYSDK	0.037	0.946	-0.390	0.037	0.099	0.882
hsa:11212	PROSC	PROSC_S244	IGSTIFGER	0.326	0.026	-0.035	0.842	0.488	0.008
hsa:55119	PRPF38B	PRPF38B_S266_S268	RSLSPR	1.581	0.357	0.861	0.027	0.118	0.886
hsa:8899	PRPF4B	PRPF4B_S328	KPIKSPSKDASSGK	-1.573	0.114	-3.881	0.004	0.000	0.000
hsa:8899	PRPF4B	PRPF4B_S427_S431_S437	SKDASPINRWSPTR	-0.044	0.794	0.535	0.086	0.249	0.014
hsa:8899	PRPF4B	PRPF4B_S330	KPIKSPSK	1.159	0.326	2.582	0.149	3.242	0.036
hsa:8899	PRPF4B	PRPF4B_M133_S142_S144	VQSGMGLILQGYESGSEEEGEIHEK	-0.016	0.937	0.232	0.008	0.174	0.503
hsa:8899	PRPF4B	PRPF4B_S387	TLSPGRR	-0.208	0.397	0.266	0.001	-0.039	0.928
hsa:5636	PRPSAP2	PRPSAP2_S227	LGIAVIHGEAQDAESDLVDGRHSPPMVR	0.242	0.003	0.050	0.682	0.248	0.045
hsa:7916	PRRC2A	PRRC2A_S1219	LIPGPLSPVAR	0.384	0.028	0.082	0.755	-0.183	0.339
hsa:23215	PRRC2C	PRRC2C_T2682	STTPTSSPFR	0.253	0.086	0.323	0.268	0.458	0.037
hsa:23362	PSD3	PSD3_S338	ESSKVPR	0.776	0.088	0.652	0.102	0.885	0.038

hsa:23550	PSD4	PSD4_S1019	SHSSPSLHQDEAPTTAK	-0.275	0.184	-0.633	0.006	-0.490	0.050
hsa:23550	PSD4	PSD4_S134_S143	QNTASPGSPVNSHLPGSPK	-0.342	0.046	-0.222	0.159	-0.327	0.068
hsa:5664	PSEN2	PSEN2_S25	TSLMSAESPTPR	-1.348	0.020	-0.987	0.157	-2.078	0.039
hsa:5782	PTPN12	PTPN12_T509	VSVTPPEESQNSDTPPRPDR	-0.267	0.027	0.216	0.542	0.269	0.155
hsa:5782	PTPN12	PTPN12_S670	DVDVSEDSPPPLPERTPESFVLASEHNTPVR	-0.235	0.022	-0.088	0.528	0.080	0.779
hsa:9698	PUM1	PUM1_S709	RDSLTGSSDLYKR	0.210	0.057	0.339	0.030	-0.177	0.766
hsa:5829	PXN	PXN_S85	FIHQQPQSSSPVYGSSAK	-0.483	0.030	-0.018	0.922	-0.001	0.998
hsa:54870	QRICH1	QRICH1_S345	GDPQQQSITHIAIPQEAYNAVHVSGSPTALAAVK	0.133	0.238	0.140	0.429	0.216	0.011
hsa:80223	RAB11FIP1	RAB11FIP1_S202	NKDSGSDTASAIIPSTTPSVDSDDESVVK	-0.400	0.151	-0.328	0.123	-1.041	0.006
hsa:80223	RAB11FIP1	RAB11FIP1_S357	HLFSSTENLAAGSWK	0.669	0.143	0.816	0.010	0.798	0.013
hsa:22841	RAB11FIP2	RAB11FIP2_S150	NNMTASMFDLSMKDK	-0.123	0.999	1.207	0.040	-0.090	0.824
hsa:9135	RABEP1	RABEP1_S407	RAQSTDSLGTSGSLQSK	-1.016	0.151	0.002	0.687	-1.179	0.015
hsa:27342	RABGEF1	RABGEF1_S607	YMSGQTSPR	0.586	0.001	0.188	0.316	0.152	0.377
hsa:10928	RALBP1	RALBP1_S645	AGKEPAKPSPSR	-0.580	0.169	-1.093	0.009	-1.098	0.021
hsa:253959	RALGAPA1	RALGAPA1_S860	RGSSPGSLEIPK	0.275	0.038	0.341	0.186	0.381	0.120
hsa:55103	RALGPS2	RALGPS2_T361	SATFPNAGPR	-1.730	0.053	-1.189	0.085	-3.422	0.001
hsa:5903	RANBP2	RANBP2_S1160	NHETDGGSAHGDDDDDGPHFEPVVPLPDKIEVK	-1.507	0.021	-1.050	0.105	-1.502	0.036
hsa:8498	RANBP3	RANBP3_S539	MPAPEPGAAPSNEEDDSDDDDVLAPSGATAAGAGDEGDGQTTGST	2.130	0.026	3.151	0.019	2.030	0.185
-	RAP1BL	RAP1BL seq: 137 - 149 + Gln->pyro-Glu (N-term Q)	QWNNCAFLESSAK	-0.165	0.722	0.050	0.510	0.144	0.033
hsa:2889	RAPGEF1	RAPGEF1_S281	VVDNSPPPALPPK	-0.285	0.089	-0.615	0.006	-0.537	0.024
hsa:2889	RAPGEF1	RAPGEF1_T254	TTGMSQSTELLPDATDEEVAPPKPPLPGIR	-0.149	0.627	0.123	0.708	0.487	0.027
hsa:2889	RAPGEF1	RAPGEF1_S652	SPDALESAQSEEEVDELSLIDHNEIMSR	1.122	0.018	0.935	0.037	0.572	0.151
hsa:64926	RASAL3	RASAL3_S166_S167_S170	VGSASSEGSIHVAMGNFR	-0.003	0.974	0.390	0.329	0.460	0.026
hsa:64926	RASAL3	RASAL3_S164_S167	VGSASSEGSIHVAMGNFR	-0.003	0.974	0.342	0.048	0.175	0.421
hsa:5925	RB1	RB1_S37	KTAATAAAAAAEPPAPPPPPPPEEDPEQDSGPEDLPLVR	-0.130	0.555	-0.230	0.473	-0.984	0.013
hsa:5930	RBBP6	RBBP6_S1277	KVTGTEGSSSTLVDYTSTSSTGGSPVRK	0.756	0.066	0.455	0.056	0.422	0.011
hsa:5930	RBBP6	RBBP6_S861	ENFSPER	0.442	0.031	0.302	0.471	0.216	0.664
hsa:8241	RBM10	RBM10_S89	HRHSPTGPPGFPR	-5.122	0.008	-5.401	0.005	-5.572	0.004
hsa:10432	RBM14	RBM14_S618	RLSESQLSFR	0.401	0.082	0.373	0.164	0.603	0.037

hsa:64783	RBM15	RBM15_S670_S674	HCAPSPDRSPELSSSR	0.084	0.892	-0.407	0.092	-0.904	0.022
hsa:64062	RBM26	RBM26_S127	LNHSPPQSSSR	-0.357	0.029	0.014	0.907	0.154	0.165
hsa:54439	RBM27	RBM27_S120	KYPSPQK	-0.480	0.005	0.184	0.038	0.084	0.725
hsa:155435	RBM33	RBM33_S765	VKPASPVAQPK	-0.199	0.335	-0.531	0.007	-0.354	0.018
hsa:9584	RBM39	RBM39_S97	YRSPYSGPK	-0.115	0.473	0.208	0.154	0.184	0.030
hsa:9584	RBM39	RBM39_S136	DKSPVREPIDNLTPEER	-0.325	0.029	-0.205	0.111	-0.132	0.399
hsa:27316	RBMX	RBMX_S88	VEQATKPSFESGR	-0.503	0.211	-0.240	0.249	-0.497	0.038
hsa:1104	RCC1	RCC1_S11	RRSPPADAIPK	-2.414	0.016	-4.457	0.001	0.000	0.000
hsa:23186	RCOR1	RCOR1_S257	EREESEDELEEANGNNPIDIEVDQNK	0.268	0.035	0.371	0.176	-0.080	0.811
hsa:92241	RCSD1	RCSD1_S216	SKAPGSPLSSEGAAGEGVR	0.049	0.602	0.345	0.033	0.710	0.133
hsa:768211	RELL1	RELL1_S244	SLMSVSGAETVNGEVPATPVKR	-0.765	0.272	-0.695	0.372	-1.949	0.016
hsa:85021	REPS1	REPS1_S540	SHSGTSPDNTAPPPPPPRPQPSHSR	-0.355	0.187	-0.671	0.003	-0.956	0.003
hsa:85021	REPS1	REPS1_T481	TGSDHTNPTSPLLVKPSDLLEENK	0.258	0.815	1.199	0.115	1.254	0.003
hsa:85021	REPS1	REPS1_S272_S273	RQSSSYDDPWK	-0.045	0.828	0.411	0.033	-0.229	0.854
hsa:85021	REPS1	REPS1_S272	RQSSSYDDPWK	-0.045	0.828	0.411	0.033	-0.229	0.854
hsa:162427	RETREG3	FAM134C_M254_S258_S260	AMDNHSDSEEELAAFCPQLDDSTVAR	0.036	0.835	-0.163	0.324	-0.053	0.034
hsa:5981	RFC1	RFC1_S69	IIYDSDSESEETLQVK	-0.144	0.031	-0.253	0.093	0.571	0.665
hsa:23180	RFTN1	RFTN1_S175	FVGVIPQYHSSVNSAGSSAPVSTANSTEDAR	-0.069	0.230	0.195	0.028	0.259	0.215
hsa:23180	RFTN1	RFTN1_S199	GDHASLENEKPGTGDVCSAPAGR	-0.498	0.101	-0.833	0.045	-0.373	0.313
hsa:28984	RGCC	RGCC_T102	LNSPTDSTPALLSATVTPQK	-0.131	0.112	0.234	0.042	0.074	0.496
hsa:28984	RGCC	RGCC_S97	LNSPTDSTPALLSATVTPQK	-0.317	0.024	0.088	0.398	-0.022	0.732
hsa:10287	RGS19	RGS19_S215	ALLLQGPSQSSSEA	-0.273	0.046	-0.061	0.323	-0.063	0.689
hsa:60626	RIC8A	RIC8A_S436_T441	GLMAGGRPEGQYSEDEDTDTDEYK	2.676	0.018	2.664	0.034	2.235	0.456
hsa:55177	RMDN3	RMDN3_S46	SQSLPNSLDYTQTSDPGR	0.075	0.659	0.262	0.353	0.428	0.001
hsa:7737	RNF113A	RNF113A_S253	YGVYEDENYEVGSDDEEIPFK	0.996	0.005	1.430	0.007	0.857	0.046
hsa:55072	RNF31	RNF31_S466	RLSAPLPSSCGDPEK	-0.387	0.344	-0.840	0.038	-0.836	0.052
hsa:80196	RNF34	RNF34_S256	ASLSDLSSLDDVEGMSVR	0.052	0.841	0.492	0.149	0.276	0.014
hsa:6102	RP2	RP2_S28	QYSWDQR	0.093	0.416	0.351	0.003	0.351	0.146
hsa:6158	RPL28	RPL28_S115	RASAILR	-0.213	0.176	-0.417	0.049	-0.159	0.303

hsa:6123	RPL3L	RL3L_S7	KFSAPR	-0.204	0.237	-0.351	0.036	-0.293	0.125
hsa:54913	RPP25	RPP25_S162	QPGYQPPNPHPGPSSPPAAPASK	-0.735	0.080	-0.161	0.555	-0.761	0.020
hsa:23248	RPRD2	RPRD2_S758	IISPGSSTPSSTR	-0.328	0.230	0.068	0.882	0.248	0.040
hsa:23248	RPRD2	RPRD2_S1099	RMSGEPIQTVESIR	0.006	0.973	0.346	0.024	0.198	0.392
hsa:6189	RPS3A	RPS3A_S236	LMELHGEGSSSGK	-0.174	0.565	0.198	0.592	0.271	0.027
hsa:6195	RPS6KA1	RPS6KA1_S363	DSPGIPPSAGAHQLFR	0.364	0.029	0.333	0.210	0.411	0.057
hsa:6197	RPS6KA3	RPS6KA3_S369	TPKDSPGIPPSANAHQLFR	-0.448	0.259	-0.460	0.276	-0.787	0.045
hsa:27330	RPS6KA6	RPS6KA6_S232	AYSFCGTVEYMAPEVVNR	-0.505	0.029	-0.483	0.037	-0.550	0.086
hsa:26750	RPS6KC1	RPS6KC1_S423_S427	FLNRSPEESFDIK	0.237	0.340	0.457	0.036	0.355	0.159
hsa:64121	RRAGC	RRAGC_S382	SCGHQTSASSLK	1.231	0.162	0.307	0.595	-5.589	0.034
hsa:6238	RRBP1	RRBP1_S1277	SHVEDGDIAGAPASSPEAPPAEQDPVQLK	-0.605	0.141	-0.738	0.039	-0.762	0.031
hsa:6239	RREB1	RREB1_S1320	QVAGDAPVEQATAETASPVHR	-0.337	0.079	-0.444	0.013	-0.483	0.019
hsa:6241	RRM2	RRM2_S20	VPLAPITDPQQLQLSPLK	-0.563	0.578	-7.989	0.045	-7.135	0.056
hsa:57142	RTN4	RTN4_S184_T188	RRGSSGSVDETLFALPAASEPVIR	-0.302	0.268	-0.618	0.033	-1.377	0.001
hsa:57142	RTN4	RTN4_S184	RRGSSGSVDETLFALPAASEPVIR	-0.943	0.004	-0.916	0.009	-1.183	0.038
hsa:9711	RUBCN	RUBCN_S410	SHSDTSIASR	1.454	0.131	2.209	0.246	3.340	0.015
hsa:6280	S100A9	S100A9_T113	MHEGDEGPGHHHKPGLGEGTP	-5.114	0.006	-7.954	0.000	-8.331	0.000
hsa:6294	SAFB	SAFB_S32	RLSDLR	0.442	0.331	1.106	0.057	1.216	0.041
hsa:25939	SAMHD1	SAMHD1_T21_S33	CDDSPRTPSNTPSAEADWSPGLELHPDYK	-0.209	0.650	1.163	0.040	1.907	0.014
hsa:25939	SAMHD1	SAMHD1_S33	TPSNTPSAEADWSPGLELHPDYK	0.359	0.049	0.304	0.480	0.240	0.635
hsa:23328	SASH1	SASH1_S813	SLPVSICR	0.117	0.490	0.310	0.045	0.248	0.148
hsa:23328	SASH1	SASH1_S839	SHSLDDLQVEPGAEQDVPTEVTEPPPQIVPEVPQK	-0.288	0.042	0.016	0.944	0.062	0.774
hsa:54440	SASH3	SASH3_S7	KPSNASEKEPTQK	0.966	0.054	2.109	0.015	2.753	0.000
hsa:54440	SASH3	SASH3_S113	ALSEEMADTLEEGSASPTSPDYSLDSPGPEK	-0.367	0.243	0.081	0.890	-1.026	0.022
hsa:54440	SASH3	SASH3_S158_S160	QASTGSELCSPSPGSGSFGEEPPAPQYTGPFCGR	2.031	0.035	2.359	0.170	1.771	0.250
hsa:58506	SCAF1	SCAF1_S498_S500	QRSPSPAPAPAPAAAAGPPTR	-0.490	0.153	-0.050	0.779	-0.453	0.013
hsa:9169	SCAF11	SCAF11_S816	KRPQSPSPR	0.909	0.183	1.418	0.253	2.008	0.001
hsa:9169	SCAF11	SCAF11_S816_S818	KRPQSPSPR	0.946	0.187	1.767	0.083	2.478	0.025
hsa:8578	SCARF1	SCARF1_S714	HFGSFQK	2.327	0.015	3.039	0.119	2.496	0.014

hsa:23256	SCFD1	SCFD1_S303	VNLEESSGVENSPAGARPK	-0.105	0.651	0.135	0.623	0.248	0.007
hsa:23513	SCRIB	SCRIB_S1561	LAEAPSPAPTPSPTPVEDLGPQTSTSPGRLSPDFAEELR	-3.832	0.003	0.122	0.978	-0.125	0.749
hsa:23513	SCRIB	SCRIB_S1508	MKSLEQDALR	-0.145	0.738	0.011	0.049	-0.270	0.942
hsa:9919	SEC16A	SEC16A_S391	QIDSSPVGGETDETTVSQNYR	-0.304	0.174	-0.161	0.036	-0.336	0.012
hsa:9919	SEC16A	SEC16A_S1184	SLHSAHSLASR	-0.251	0.327	-0.659	0.052	-0.986	0.017
hsa:9919	SEC16A	SEC16A_T1876	FANLTPSR	0.423	0.098	0.555	0.041	0.698	0.111
hsa:9919	SEC16A	SEC16A_S1191	SSLSSHSHQSQIYR	0.116	0.722	0.480	0.028	0.273	0.171
hsa:10952	SEC61B	SEC61B_S49	TTSAGTGGMWR	0.171	0.558	0.593	0.014	0.825	0.019
hsa:10952	SEC61B	SEC61B_S13	PGPTPSGTNVGSSGR	-0.005	0.944	0.342	0.034	0.258	0.094
hsa:6400	SEL1L	SEL1L_S41	TTLTSDESVKDHTTAGR	-0.022	0.931	0.265	0.030	0.266	0.313
hsa:989	SEPT7	SEPT7_T426	ILEQQNSSRTLEK	0.933	0.542	3.741	0.132	4.463	0.010
hsa:5345	SERPINF2	A2AP_S459	DFLQSLK	-2.166	0.068	-0.873	0.387	-3.608	0.010
hsa:29072	SETD2	SETD2_S624	LNDSPTLK	-0.055	0.903	-0.041	0.956	-0.784	0.033
hsa:7536	SF1	SF1_S80_S82	TGDLGIPPNPEDRSPSPEPIYNSEGK	0.721	0.038	0.981	0.010	0.606	0.081
hsa:10291	SF3A1	SF3A1_S329	FGESEEVEMEVESDEEDDKQEK	0.457	0.022	0.144	0.845	-0.493	0.407
hsa:23451	SF3B1	SF3B1_S194	VVNGAAASQPPSKR	-0.826	0.073	-2.159	0.002	-2.156	0.002
hsa:23616	SH3BP1	SH3BP1_S262	ENHGQADHSPSMTATHFPR	-0.712	0.041	-0.398	0.221	-0.707	0.033
hsa:6452	SH3BP2	SH3BP2_S416	SPPDGQSFR	0.316	0.017	0.225	0.034	0.530	0.012
hsa:80851	SH3BP5L	SH3BP5L_S362	GLSDHVSLDGQELGTR	0.068	0.681	0.242	0.154	0.296	0.009
hsa:30011	SH3KBP1	SH3KBP1_S183	ETTGSESDGGDSSSTK	2.661	0.040	4.059	0.028	3.892	0.220
hsa:57698	SHTN1	SHTN1_S494	VTAEADSSSPTGILATSESK	-0.160	0.115	0.045	0.718	0.094	0.021
hsa:6494	SIPA1	SIPA1_S74	AHSHEEASRPAATSTR	-0.996	0.337	-2.011	0.166	-6.572	0.001
hsa:22933	SIRT2	SIRT2_S366	EHASIDAQSGAGVPNPSTSASPK	-0.140	0.397	-0.213	0.191	-0.274	0.049
hsa:22933	SIRT2	SIRT2_S368	EHASIDAQSGAGVPNPSTSASPK	-0.370	0.039	0.011	0.939	0.159	0.247
hsa:8935	SKAP2	SKAP2_S283	KMSQDSVHHTSGDK	-1.498	0.140	-2.569	0.048	-7.759	0.000
hsa:57606	SLAIN2	SLAIN2_S247	SSDRNPPLSPQSSIDSELSASELDEDSIGSNYK	-0.244	0.846	-0.259	0.934	-1.485	0.041
hsa:9990	SLC12A6	SLC12A6_S32	IDDIPGLSDTSPDLSSR	-0.354	0.045	-0.030	0.701	-0.144	0.285
hsa:6566	SLC16A1	SLC16A1_S461	KESKEEETSIDVAGKPNEVTK	-1.295	0.134	-2.476	0.018	-1.862	0.062
hsa:6575	SLC20A2	SLC20A2_S316	ALSMTHGSVK	-0.488	0.276	-0.898	0.057	-1.065	0.021

hsa:7922	SLC39A7	SLC39A7_S276	EKQSSEEEEKETR	0.875	0.181	1.677	0.095	2.823	0.018
hsa:6522	SLC4A2	SLC4A2_T141	ALTQPSPVSTPSSVQFFLQEDDSADRK	-0.141	0.503	0.109	0.186	0.121	0.041
hsa:9497	SLC4A7	SLC4A7_S407	GNGSGGSRENSTVDFSK	-0.398	0.282	-1.252	0.006	-0.587	0.177
hsa:9498	SLC4A8	S4A8_S237	SFAEVGK	-1.528	0.006	-0.236	0.622	-0.099	0.844
hsa:84138	SLC7A6OS	SLC7A6OS_S308	EFGYDSPHDLDSD	-0.177	0.299	-0.052	0.592	-0.214	0.027
hsa:6546	SLC8A1	SLC8A1_S285	GMIIEHEGDRPSSKTEIEMDGK	-1.061	0.105	-1.229	0.048	-0.783	0.157
hsa:6548	SLC9A1	SLC9A1_S693	LDSPTMSR	0.370	0.253	0.643	0.016	0.316	0.140
hsa:79811	SLTM	SLTM_M993_S1002	AGAGMITQHSSNASPINR	0.148	0.570	0.295	0.042	0.174	0.503
hsa:10569	SLU7	SLU7_S215	LVEQANSPK	0.199	0.544	0.012	0.946	0.424	0.033
hsa:10944	SMAP	SMAP_S25	RSASPDDDLGSSNWEAADLGNEERK	-0.253	0.312	-0.306	0.092	-0.299	0.015
hsa:10944	SMAP	SMAP_S17	RSASPDDDLGSSNWEAADLGNEERK	-2.620	0.016	-0.335	0.498	-1.217	0.151
hsa:6595	SMARCA2	SMARCA2_S1377	GRPPAEKLSPNPPK	-0.201	0.134	-0.507	0.009	-0.170	0.319
hsa:56916	SMARCAD1	SMARCAD1_S98	GIQYIDLSSDSEDVVSPNCSNTVQEK	0.480	0.534	0.515	0.013	0.367	0.365
hsa:6599	SMARCC1	SMARCC1_S328_S330	KHSPSPPPPTPTESR	-0.222	0.020	0.033	0.734	-0.027	0.828
hsa:6601	SMARCC2	SMARCC2_S302	KRSPSPSPTPEAK	0.901	0.114	1.363	0.254	0.781	0.016
hsa:10051	SMC4	SMC4_S28	RREEGPPPPSPDGASSDAEPEPPSGR	-0.482	0.251	-0.312	0.169	-0.970	0.002
hsa:10051	SMC4	SMC4_T44	TESPATAAETASEELDNR	0.187	0.033	0.030	0.915	-0.566	0.258
hsa:27044	SND1	SND1_S426	VNVTVDYIRPASPATETVPAFSER	0.270	0.462	0.478	0.177	0.885	0.027
hsa:79753	SNIP1	SNIP1_S52	RPDHSGGSPSPPTSEPAR	-0.403	0.273	-0.657	0.080	-0.685	0.042
hsa:79753	SNIP1	SNIP1_S35	QERLSPEVAPPAHR	-0.428	0.049	-0.500	0.047	-0.499	0.258
hsa:6641	SNTB1	SNTB1_S219	GSPVSEIGWETPPPESPR	0.042	0.644	0.613	0.063	1.113	0.000
hsa:6645	SNTB2	SNTB2_S222	KPSLVSDLPWEGAAPQSPSFSGSEDSGSPK	0.077	0.737	0.101	0.319	0.274	0.031
hsa:6645	SNTB2	SNTB2_S393	SPSLGSDLTFATR	-0.181	0.101	0.206	0.024	0.322	0.141
hsa:22938	SNW1	SNW1_S224	GPPSPPAPVMHSPSR	-0.274	0.103	-0.445	0.038	-0.638	0.008
hsa:22938	SNW1	SNW1_S224_S232	GPPSPPAPVMHSPSR	-0.269	0.348	-0.332	0.186	-0.245	0.037
hsa:51429	SNX9	SNX9_S122	SGNWESSEGWGAQPEGAGAQR	2.831	0.026	3.244	0.017	3.200	0.021
hsa:140710	SOGA1	SOGA1_S1017	VYYSPPVAR	0.199	0.150	0.185	0.008	0.440	0.175
hsa:6651	SON	SON_S2009_S2011_S2013	RRSFSISPVR	0.704	0.076	0.694	0.104	0.752	0.018
hsa:6651	SON	SON_S283	SVESTSPEPSK	0.454	0.036	0.298	0.490	-0.145	0.672

hsa:6654	SOS1	SOS1_S1210	TSISDPPESPPLLPPREPVR	0.242	0.006	-0.018	0.962	0.203	0.736
hsa:6672	SP100	SP100_S410	VIGQDHDFSESSEEEAPAEASSGALR	-0.299	0.275	-0.342	0.092	-0.375	0.025
hsa:6672	SP100	SP100_S389	KLSTFR	-1.417	0.032	-1.638	0.038	-1.119	0.108
hsa:3431	SP110	SP110_S380	VTQGAASPGHGIQEK	-0.471	0.046	0.077	0.302	0.039	0.876
hsa:9043	SPAG9	SPAG9_S705	DGGSVVGASVFYK	0.117	0.486	0.195	0.155	-0.853	0.005
hsa:9043	SPAG9	SPAG9_S185	LHQLSGSDQLESTAHSR	-0.325	0.073	-0.200	0.026	-0.193	0.213
hsa:9043	SPAG9	SPAG9_S732_S733	SASQSSLDKLDQELK	0.262	0.006	0.056	0.727	-0.171	0.377
hsa:9043	SPAG9	SPAG9_S339	NVSTGSAENEEKSEVQAIIESTPELDMDKDLSGYK	0.382	0.516	0.801	0.001	0.370	0.638
hsa:25803	SPDEF	SPDEF_S308	LSRSIR	0.912	0.179	1.583	0.001	1.738	0.000
hsa:23013	SPEN	SPEN_S727	SQSPVHLR	-0.294	0.304	-0.645	0.043	-0.444	0.082
hsa:152185	SPICE1	SPICE1_T235	IATQSQITPPGTPSSALSSGEQR	-0.233	0.526	0.186	0.372	0.385	0.033
hsa:6696	SPP1	SPP1_S275_S280	EFHSHEFHSHEDMLVVDPK	-1.656	0.105	-1.497	0.201	-7.165	0.001
hsa:6696	SPP1	SPP1_S303_S308	FRISHELDSASSEVN	0.347	0.454	0.266	0.181	0.490	0.020
hsa:6696	SPP1	SPP1_S62	QNLLAPQNAVSSEETNDFK	-0.518	0.102	-0.206	0.583	-0.257	0.027
hsa:6696	SPP1	SPP1_S63	QNLLAPQNAVSSEETNDFK	0.717	0.147	1.027	0.016	0.392	0.274
hsa:6696	SPP1	SPP1_S234	AIPVAQDLNAPSDWDSRGKDSYETSQLDDQSAETHSHK	-2.140	0.041	0.132	0.964	-0.901	0.335
hsa:6696	SPP1	SPP1_S195	RPDIQYPDATDEDITSHMESEELNGAYK	-1.070	0.049	-0.700	0.222	-0.141	0.618
hsa:6696	SPP1	SPP1_S215	AIPVAQDLNAPSDWDSR	0.494	0.038	0.375	0.154	0.198	0.741
hsa:6696	SPP1	SPP1_T185_S191_S195	RPDIQYPDATDEDITSHMESEELNGAYK	-0.270	0.018	0.026	0.823	-0.011	0.930
hsa:6709	SPTAN1	SPTAN1_S1031	KLDPAQSASRENLLEEQGSIALR	0.378	0.506	-0.105	0.036	0.109	0.399
hsa:8878	SQSTM1	SQSTM1_S24	RFSFCCSPEPEAEAEAAAGPGPCER	-0.545	0.062	-0.391	0.054	-0.490	0.015
hsa:8878	SQSTM1	SQSTM1_S272	LTPVSPESSSTEEK	-0.213	0.421	0.168	0.044	-0.019	0.620
hsa:6714	SRC	SRC_S17	RRSLEPAENVHGAGGGAFPASQTPSKPASADGHR	-3.531	0.041	-3.078	0.066	0.000	0.004
hsa:23380	SRGAP2	SRGAP2_S1013	SASTAGDIACAFRPVK	0.494	0.217	0.657	0.125	0.452	0.025
hsa:6733	SRPK2	SRPK2_S497	TVSASSTGDLPK	0.432	0.135	0.348	0.109	0.738	0.029
hsa:6734	SRPR	SRPR_S298	GTGSGGQLQDLDCSSSDDEGAAQNSTKPSATK	-0.080	0.486	0.247	0.220	0.406	0.049
hsa:10250	SRRM1	SRRM1_S597	RYSPPIQR	0.235	0.048	0.284	0.040	0.555	0.001
hsa:10250	SRRM1	SRRM1_S389_S393	RLSPSASPPR	0.285	0.077	0.407	0.011	0.650	0.006
hsa:10250	SRRM1	SRRM1_S316	RRPSPR	1.835	0.184	3.972	0.018	3.488	0.008

hsa:10250	SRRM1	SRRM1_S402_T406	HRPSPPATPPPK	-0.505	0.071	-0.262	0.313	-0.269	0.008
hsa:10250	SRRM1	SRRM1_S683	RHSPSPRPR	-3.633	0.027	-4.834	0.008	-4.746	0.013
hsa:10250	SRRM1	SRRM1_S713_S715	RQSPSPSTRPIR	-0.532	0.141	-0.291	0.059	-0.407	0.013
hsa:10250	SRRM1	SRRM1_S414	HSPTPQQSNR	2.340	0.082	2.960	0.025	3.586	0.016
hsa:10250	SRRM1	SRRM1_S414_T416	TRHSPTPQQSNR	2.264	0.304	3.071	0.024	3.093	0.023
hsa:10250	SRRM1	SRRM1_S752_S754_S756	SVSGSPEPAAK	2.380	0.362	3.003	0.041	3.133	0.026
hsa:10250	SRRM1	SRRM1_S605_S607	RYSPSPPPKR	0.214	0.063	0.318	0.134	0.323	0.036
hsa:10250	SRRM1	SRRM1_S626_S628	RASPSPPPKR	0.706	0.133	0.655	0.118	0.838	0.043
hsa:10250	SRRM1	SRRM1_T220	KEKTPELPEPSVK	-0.384	0.043	-0.458	0.041	-0.337	0.087
hsa:10250	SRRM1	SRRM1_S769	KPPAPPSPVQSQSPSTNWSPAVPVK	0.534	0.121	0.672	0.019	0.544	0.140
hsa:10250	SRRM1	SRRM1_S616	RTASPPPPPKR	-0.159	0.026	0.314	0.627	0.549	0.226
hsa:10250	SRRM1	SRRM1_S389_S391_S393	RLSPSASPPR	0.037	0.016	0.043	0.451	-0.540	0.227
hsa:10250	SRRM1	SRRM1_S551	SPSPPPTR	1.031	0.025	1.060	0.094	0.621	0.370
hsa:10250	SRRM1	SRRM1_S874	KETESEAEDNLDDLEK	0.549	0.011	0.172	0.600	-0.209	0.655
hsa:23524	SRRM2	SRRM2_S2132	CRSPGMLEPLGSSR	-0.067	0.671	0.032	0.893	0.241	0.005
hsa:23524	SRRM2	SRRM2_S508_S510	SRSPQWR	0.334	0.217	0.414	0.078	0.410	0.005
hsa:23524	SRRM2	SRRM2_S1869	SRASPATHR	3.054	0.029	4.116	0.233	4.624	0.006
hsa:23524	SRRM2	SRRM2_S1403_S1404	AGMSSNQSISSPVLDAVPR	-0.159	0.165	0.114	0.537	0.162	0.007
hsa:23524	SRRM2	SRRM2_S895	HSCSGSSPPR	0.952	0.225	1.818	0.093	2.890	0.015
hsa:23524	SRRM2	SRRM2_S778_S780_S783	SLSGSSPCPK	5.144	0.480	4.736	0.184	4.478	0.023
hsa:23524	SRRM2	SRRM2_S440	HASSSPESPKPAPAPGSHR	-0.178	0.653	-0.152	0.640	-0.983	0.024
hsa:23524	SRRM2	SRRM2_S1320	ELSNSPLR	-0.521	0.017	0.367	0.205	-0.776	0.028
hsa:23524	SRRM2	SRRM2_S322_S323	RGEGDAPFSEPGTTSTQRPSSPETATK	-0.255	0.179	-0.268	0.018	-0.378	0.031
hsa:23524	SRRM2	SRRM2_S973	YSHSGSSSPDTK	1.911	0.106	2.481	0.037	2.477	0.038
hsa:23524	SRRM2	SRRM2_S1854_S1857	SRTSPAPWK	0.596	0.264	0.692	0.306	0.971	0.048
hsa:23524	SRRM2	SRRM2_T1043	SSTPPGESYFGVSSLQLK	-0.421	0.045	0.296	0.499	-0.397	0.171
hsa:23524	SRRM2	SRRM2_T1531	TVARTPLGQR	-0.527	0.047	-0.388	0.000	-0.397	0.216
hsa:23524	SRRM2	SRRM2_S2581	RVPSPTPAPK	-0.004	0.864	-0.327	0.006	-0.278	0.230
hsa:23524	SRRM2	SRRM2_S876	RSCFESSPDPELK	-0.327	0.316	-0.260	0.035	-0.157	0.319

hsa:23524	SRRM2	SRRM2_S2100_T2104	NHSGSRTPPVALNSSR	-0.202	0.346	0.463	0.049	0.180	0.361
hsa:23524	SRRM2	SRRM2_S2020_T2022	SRTPPAIR	0.230	0.087	0.376	0.016	0.191	0.367
hsa:23524	SRRM2	SRRM2_T2034	TPLLPR	-0.137	0.052	-0.438	0.003	-0.150	0.447
hsa:23524	SRRM2	SRRM2_T1003	AQTPPGPSLSGSK	-0.103	0.005	-0.217	0.009	0.007	0.998
hsa:51593	SRRT	SRRT_S74	HELSPPQK	0.560	0.012	0.064	0.898	0.283	0.200
hsa:6426	SRSF1	SRSF1_S199_S201	VKVDGPRSPSYGR	0.101	0.641	0.413	0.020	0.381	0.158
hsa:10772	SRSF10	SRSF10_S131_S133	SRSFDYNYR	0.340	0.005	0.667	0.011	0.609	0.003
hsa:10772	SRSF10	SRSF10_S253	SWTSPK	0.342	0.207	0.466	0.034	0.525	0.043
hsa:10772	SRSF10	SRSF10_S133	SFDYNYR	0.232	0.054	0.232	0.032	0.215	0.088
hsa:6428	SRSF3	SRSF3_S108	RRSPPPR	0.817	0.215	1.905	0.071	2.859	0.017
hsa:6429	SRSF4	SRSF4_S431	GESENAGTNQETR	0.718	0.046	0.740	0.329	0.430	0.266
hsa:6429	SRSF4	SRSF4_S458	SKPNLPSESR	-0.261	0.026	0.152	0.146	-0.082	0.427
hsa:6432	SRSF7	SRSF7_S192_S194	YFQSPSR	1.689	0.018	1.496	0.078	1.883	0.062
hsa:8683	SRSF9	SRSF9_Y214	GSPHYFSPFRPY	0.339	0.072	0.288	0.121	0.478	0.044
hsa:6741	SSB	SSB_S94	SPSKPLPEVTDEYKNDVK	-0.455	0.276	-0.630	0.050	-0.788	0.013
hsa:54434	SSH1	SSH1_S897	SPPPFFYR	1.068	0.001	0.711	0.020	0.849	0.322
hsa:54961	SSH3	SSH3_S9	SPPGSGASTPVGPWDQAVQR	-0.133	0.424	0.091	0.390	-0.088	0.006
hsa:6749	SSRP1	SSRP1_S444	EGMNPSYDEYADSDEDQHDAYLER	3.016	0.030	2.960	0.271	3.353	0.041
hsa:6767	ST13	ST13_S76_S79	ADEPSSEESDLEIDKEGVIEPDTDAPQEMGDENAEITEEMMDQANDKK	-0.023	0.291	-0.143	0.318	-0.026	0.012
hsa:6786	STIM1	STIM1_S618	SHSPSSPDPDTPSPVGDSR	0.168	0.898	1.012	0.022	0.867	0.071
hsa:6786	STIM1	STIM1_S519	DLTHSDSESSLHMSDR	-0.149	0.290	-0.300	0.002	-0.192	0.162
hsa:57620	STIM2	STIM2_S719	ISSIPHDLCHNGEK	-0.157	0.226	-0.411	0.044	-0.412	0.130
hsa:30968	STOML2	STOML2_T327	APVPGTPDSLSSGSSR	-0.720	0.012	-0.672	0.001	-0.730	0.026
hsa:85369	STRIP1	STRIP1_S335	AASPPASASDLIEQQQK	0.033	0.949	-0.323	0.024	-0.199	0.065
hsa:6801	STRN	STRN_S245	FLESAAADFSDEDEDDDVDGR	1.099	0.029	0.766	0.093	0.325	0.417
hsa:64426	SUDS3	SUDS3_S236_S237	RPASPSSPEHLPATPAESPAQR	0.153	0.174	0.288	0.114	0.324	0.017
hsa:64426	SUDS3	SUDS3_S237	RPASPSSPEHLPATPAESPAQR	-0.284	0.339	0.232	0.526	0.320	0.019
hsa:23512	SUZ12	SUZ12_S546	ASMSEFLESEDGEVEQQR	-0.088	0.004	0.127	0.550	0.037	0.592
hsa:94056	SYAP1	SYAP1_T248	EQDLPLAEAVRPKTPPVVIK	-0.062	0.954	0.678	0.030	0.240	0.525

hsa:8189	SYMPK	SYMPK_S494	RLSAQGQAISVVGSLSSMSPLEEEAPQAK	0.416	0.254	0.266	0.258	0.468	0.011
hsa:23345	SYNE1	SYNE1_S8223	LPLPDDEHDLSDR	-0.446	0.348	-1.168	0.017	-0.713	0.185
hsa:8867	SYNJ1	SYNJ1_S1293	SRSSHSLPSEASSQPQVK	-0.801	0.238	-1.171	0.088	-4.394	0.002
hsa:11276	SYNRG	SYNRG_S855	HVMSDSSLDLPTVSGQHPPAADIEDLK	0.107	0.622	0.791	0.079	1.352	0.034
hsa:6883	TAF12	TAF12_S51	LSPENNQVLTK	-0.539	0.111	-0.560	0.043	-0.636	0.032
hsa:117289	TAGAP	TAGAP_S400	SEGDFPVPR	-0.138	0.426	0.467	0.036	0.076	0.878
hsa:26115	TANC2	TANC2_S1530	EYPSPPPSPLR	1.841	0.288	2.617	0.020	2.625	0.014
hsa:23216	TBC1D1	TBC1D1_S627	YHSVSTETPHER	-0.458	0.329	-0.980	0.130	-1.659	0.021
hsa:26000	TBC1D10B	TBC1D10B_S667_S678	SRGSRAAGGAPSPPPPVR	-0.362	0.046	-0.299	0.182	-0.520	0.009
hsa:26000	TBC1D10B	TBC1D10B_S22	RHGAPAAPSPPPR	-0.430	0.063	-0.868	0.026	-0.860	0.014
hsa:25771	TBC1D22A	TBC1D22A_S167	SQSLPHSATVTLGGTSDPSTLSSSALSER	0.586	0.154	0.909	0.048	0.717	0.129
hsa:55773	TBC1D23	TBC1D23_S571	GVKPVFSIGDEEEYDTDEIDSSSMSDDDRK	-0.519	0.251	-1.127	0.007	-1.808	0.006
hsa:4943	TBC1D25	TBC1D25_S506	QASLDGLQQLR	0.139	0.551	0.235	0.022	0.031	0.783
hsa:9779	TBC1D5	TBC1D5_S554	EFTGSPPSSATKK	-0.523	0.123	-0.883	0.037	-1.426	0.003
hsa:9779	TBC1D5	TBC1D5_S541	NISSSPSVESLPGGR	0.107	0.461	0.175	0.014	0.399	0.159
hsa:11138	TBC1D8	TBC1D8_S464	SPLMHPDALVTAFQQSGSQSPDSR	0.634	0.034	0.615	0.033	0.051	0.884
hsa:23061	TBC1D9B	TBC1D9B_S411	KASVVDPSTESSPAPQEGSEQPASPASPLSSR	-0.024	0.641	0.247	0.220	0.423	0.034
hsa:23061	TBC1D9B	TBC1D9B_S420_S427	KASVVDPSTESSPAPQEGSEQPASPASPLSSR	0.121	0.916	-1.522	0.010	-0.451	0.354
hsa:6949	TCOF1	TCOF1_S1378	LGAGEGGEASVSPEK	-0.923	0.003	-1.253	0.001	-1.479	0.005
hsa:6949	TCOF1	TCOF1_S1228	LDSSPSVSSTLAAK	-0.044	0.827	0.213	0.189	0.220	0.009
hsa:6949	TCOF1	TCOF1_S906	AALAPAKESPR	-0.401	0.130	-0.384	0.085	-0.385	0.019
hsa:55852	TEX2	TEX2_S732	SSGVSGGKPGLLPAHSR	0.121	0.203	0.350	0.017	0.260	0.260
hsa:7019	TFAM	TFAM_S195	NLSDSEKELYIQHAK	-0.337	0.045	-0.331	0.424	-0.634	0.301
hsa:7942	TFEB	TFEB_S122	FAAHISPAQGSPKPPPAASPGVR	0.143	0.468	0.394	0.016	0.433	0.181
hsa:24144	TFIP11	TFIP11_S98	GAAEEAELEDSDDEEKPVKQDDFPK	0.366	0.034	0.389	0.523	-0.255	0.444
hsa:29844	TFPT	TFPT_S180	RTPAPPEPGSPAPGEGPSGR	-0.909	0.054	-0.787	0.007	-1.017	0.112
hsa:7052	TGM2	TGM2_M227	VVSGMVNCNDDQGVLLGR	-0.107	0.244	-0.471	0.039	-0.080	0.741
hsa:9967	THRAP3	THRAP3_S698	HGLAHDEMKSPR	-1.826	0.110	-3.166	0.018	-7.885	0.000
hsa:9967	THRAP3	THRAP3_S672	NKKSPEIHR	-2.349	0.071	-5.212	0.004	-9.807	0.001

hsa:9967	THRAP3	THRAP3_S624	SPSELFAQHIVTIVHHVK	-0.839	0.034	-0.860	0.013	-0.959	0.044
hsa:9967	THRAP3	THRAP3_S535	SSSPPPR	1.108	0.104	1.763	0.031	1.693	0.137
hsa:9967	THRAP3	THRAP3_S682	RIDISPSTFRK	-0.034	0.685	-0.363	0.010	-0.144	0.148
hsa:9967	THRAP3	THRAP3_T327	SPPSTGSTYGSSQKEESAASGGAAYTKR	-0.307	0.158	-0.380	0.014	-0.490	0.196
hsa:9967	THRAP3	THRAP3_S320	SPPSTGSTYGSSQK	-0.243	0.050	0.139	0.524	0.176	0.220
hsa:9967	THRAP3	THRAP3_S53_S55	SYSPAHNR	1.095	0.355	0.921	0.005	0.400	0.577
hsa:55623	THUMPD1	THUMPD1_S88	FTDKDQQPSGSEGEDDDAEAALKK	-0.363	0.291	-0.513	0.162	-1.183	0.021
hsa:55623	THUMPD1	THUMPD1_S86_S88	FTDKDQQPSGSEGEDDDAEAALKK	0.723	0.026	0.557	0.122	0.264	0.555
hsa:7074	TIAM1	TIAM1_S231	ANSLGDLYAQK	-0.215	0.114	-0.246	0.037	-0.245	0.023
hsa:93643	TJAP1	TJAP1_S300	GSPEEELPLPAFEK	-0.265	0.043	-0.055	0.653	-0.158	0.218
hsa:7090	TLE3	TLE3_S289	DAPTSPASVASSSSTPSSK	-0.065	0.453	0.421	0.046	0.521	0.022
hsa:7090	TLE3	TLE3_S286	DAPTSPASVASSSSTPSSK	-0.166	0.334	0.224	0.269	0.324	0.040
hsa:7090	TLE3	TLE3_S203	ESSANNSVSPSESLR	-0.126	0.774	0.374	0.035	0.357	0.124
hsa:9874	TLK1	TLK1_S159	ISDYFEYQGGNGSSPVR	0.018	0.889	-0.249	0.230	-0.820	0.038
hsa:7094	TLN1	TLN1_S405	SKDHFGLEGDEESTMLEDSVSPK	-1.122	0.023	-0.938	0.057	-1.502	0.006
hsa:222865	TMEM130	TMEM130_T241	LQETLR	0.473	0.414	0.566	0.411	0.917	0.048
hsa:7112	TMPO	TMPO_S66_S67	GPPDFSSDEEREPTPVLGSGAAAAGR	0.798	0.084	1.293	0.042	0.716	0.237
hsa:7112	TMPO	TMPO_S184	QNGSNDSDRYSDNEEDSKIELK	0.407	0.028	0.120	0.712	-0.463	0.427
hsa:7112	TMPO	TMPO_S67_T74	GPPDFSSDEEREPTPVLGSGAAAAGR	-0.272	0.004	0.241	0.465	0.230	0.449
hsa:10318	TNIP1	TNIP1_S266	EGASGRPGSPK	4.611	0.109	4.939	0.003	4.964	0.103
hsa:85456	TNKS1BP1	TNKS1BP1_S836	SQEADVQDWEFR	-0.182	0.446	-0.248	0.305	-0.628	0.035
hsa:85456	TNKS1BP1	TNKS1BP1_S601	YESQEPLAGQESPLPLATR	-0.407	0.344	-0.621	0.109	-1.127	0.048
hsa:85456	TNKS1BP1	TNKS1BP1_S1385	HNGSLSPGLEAR	-0.242	0.039	-0.538	0.030	-0.651	0.271
hsa:23112	TNRC6B	TNRC6B_S879	DEEPSGWEEPSPQSISR	0.525	0.251	0.704	0.109	0.939	0.043
hsa:7145	TNS1	TNS1_S1297	HPGAHQGNLASGLHSNAIASPGSPSLGR	-0.652	0.303	-0.379	0.397	-6.104	0.000
hsa:7145	TNS1	TNS1_S1381	QGSPTPALPEK	1.350	0.198	2.274	0.103	2.997	0.022
hsa:7145	TNS1	TNS1_S1124	SGSLGQPSPSAQR	-0.301	0.202	-0.571	0.125	-0.554	0.045
hsa:64759	TNS3	TNS3_S874	HPPFSPPEPPLSSPASQHK	0.083	0.394	0.065	0.609	0.250	0.038
hsa:64759	TNS3	TNS3_S660	GVGSGPHPPDTQQPSPSK	-0.289	0.455	-0.336	0.014	-0.558	0.123

hsa:64759	TNS3	TNS3_S1441	VMIGSPK	-0.209	0.311	-0.403	0.047	-0.216	0.395
hsa:64759	TNS3	TNS3_S1154	ASEAASPLPDSPGDKLVIVK	0.198	0.176	-0.121	0.049	-0.113	0.636
hsa:10043	TOM1	TOM1_S160	KGLEFPMTDLDMLSPIHTPQR	-2.046	0.025	-0.045	0.690	-0.823	0.289
hsa:9868	TOMM70	TOMM70A_S91	ASPAPGSGHPEGPGAHLDMNSLDR	-0.257	0.004	0.280	0.203	0.218	0.277
hsa:7155	TOP2B	TOP2B_S1466	FDSNEEDSASVFSPSFGLK	0.887	0.008	0.500	0.166	0.004	0.954
hsa:10210	TOPORS	TOPORS_S194	NASVYSPSGPVNR	0.557	0.035	0.526	0.115	0.370	0.544
hsa:7158	TP53BP1	TP53BP1_S1114	MVIQGPSSPQGEAMVTDVLEDQK	0.291	0.511	0.227	0.221	0.622	0.004
hsa:7158	TP53BP1	TP53BP1_S294	SPEPEVLSTQEDLFDQSNK	2.175	0.182	2.342	0.175	1.898	0.031
hsa:7167	TPI1	TPI1_S58	QSLGELIGTLNAAK	0.321	0.657	-0.053	0.737	-0.496	0.044
hsa:7175	TPR	TPR_S379	GAILSEEELAAMSPTAAAVAK	0.810	0.361	1.000	0.005	0.292	0.663
hsa:286262	TPRN	TPRN_S369	GDLGPASPSQELGSQPVPGGDGAPALGK	0.277	0.140	0.682	0.077	0.569	0.029
hsa:29896	TRA2A	TRA2A_T202	AHTPTPGIYMGRPTHSGGGGGGGGGGGGGGGR	-0.509	0.015	-0.296	0.130	-0.458	0.099
hsa:29896	TRA2A	TRA2A_S86_T88	SYTPEYR	0.554	0.100	0.499	0.001	0.350	0.263
hsa:6434	TRA2B	TRA2B_S264_S266	SPSPYYSR	0.744	0.066	0.820	0.056	1.073	0.016
hsa:6434	TRA2B	TRA2B_S264	RRSPSPYYSR	-0.625	0.323	-0.509	0.442	-2.513	0.032
hsa:22906	TRAK1	TRAK1_S919	SFPTMVGSSMQMK	0.672	0.079	0.472	0.049	0.347	0.463
hsa:7109	TRAPPC10	TRAPPC10_S685	SPSDNSLNTTGIICR	-0.130	0.372	0.152	0.197	0.252	0.010
hsa:83696	TRAPPC9	TRAPPC9_S953	MAIQVDKFNFESFPESPGEK	-0.097	0.621	-0.213	0.388	-0.669	0.041
hsa:7706	TRIM25	TRIM25_S100	ASAPSPNAQVACDHCLK	-0.087	0.674	0.590	0.244	0.391	0.001
hsa:10155	TRIM28	TRIM28_S473	SRSGEGEVSGLMR	0.165	0.266	0.181	0.187	0.387	0.001
hsa:10155	TRIM28	TRIM28_S471	SRSGEGEVSGLMR	0.292	0.295	0.276	0.200	0.515	0.021
hsa:10155	TRIM28	TRIM28_S50	STAPSAAASASASAAASSPAGGGAEALELLEHCGVCR	-0.255	0.041	-0.034	0.851	0.045	0.789
hsa:9320	TRIP12	TRIP12_S310_S312	KRSESPPAELPSLR	0.638	0.156	0.580	0.005	0.746	0.098
hsa:55621	TRMT1	TRMT1_S625	GDQCCYSHSPPTPR	-0.723	0.022	0.435	0.564	0.550	0.346
hsa:145567	TTC7B	TTC7B_S160	LPISSSTSNLHVDR	-0.036	0.622	0.108	0.170	0.456	0.035
hsa:11334	TUSC2	TUSC2_S50	RGSMFYDEDGDLAHEFYEETIVTK	-1.062	0.022	-0.801	0.075	-1.367	0.012
hsa:5756	TWF1	TWF1_S143	YLLSQSSPAPLTAAEEELR	0.263	0.085	0.241	0.055	0.258	0.021
hsa:199746	U2AF1L4	U2AF4_S194	RRSPPR	1.835	0.184	3.972	0.018	3.488	0.008
hsa:7317	UBA1	UBA1_S810	IHVSDQELQSANASVDDSR	0.144	0.309	0.399	0.116	0.497	0.026

hsa:7317	UBA1	UBA1_S13	RVSGPDPKPGSNCSPAQSVLSEVPSVPTNGMAK	0.625	0.278	0.901	0.016	0.649	0.117
hsa:9898	UBAP2L	UBAP2L_S609	RYPSSISSSPQK	0.302	0.075	0.089	0.379	0.388	0.020
hsa:51465	UBE2J1	UBE2J1_S268	RLSTSPDVIQGHQPR	0.183	0.186	0.376	0.016	0.235	0.275
hsa:23352	UBR4	UBR4_T2724	SNTPMGDKDDDDDDDADEKMQSSGIPNGGHIR	-1.365	0.045	-0.905	0.048	-0.806	0.086
hsa:23352	UBR4	UBR4_S2719	RHVTLPSSPR	1.198	0.026	1.007	0.123	0.835	0.097
hsa:51366	UBR5	UBR5_S1549	RISQSQPVR	-0.080	0.561	-0.461	0.018	-0.257	0.178
hsa:23376	UFL1	UFL1_S458	KDDDSDDESQSSHTGK	5.820	0.321	8.464	0.069	8.787	0.001
hsa:81622	UNC93B1	UNC93B1_S550	YLEEDNSDESDAEGEHGDGAEEEAPPAGPRPGPEPAGLGR	-0.384	0.184	-1.865	0.033	-1.341	0.090
hsa:8615	USO1	USO1_S942	DLGHPVEEEDELESGDQEDEDDESEDPGKDLDHI	-0.087	0.030	-0.601	0.603	-1.147	0.155
hsa:9097	USP14	USP14_S143	YAGALRASGEMASAQYITAALR	-0.346	0.337	-0.161	0.707	-0.911	0.032
hsa:23358	USP24	USP24_S2604	HLQQGSESPMMIGELR	2.165	0.179	2.540	0.017	2.983	0.014
hsa:23358	USP24	USP24_S2047	VSDQNSPVLPK	0.447	0.010	0.000	0.831	0.185	0.711
hsa:8078	USP5	USP5_S785	SAADSISESVPVGPK	-0.787	0.030	-0.286	0.373	-0.039	0.822
hsa:8674	VAMP4	VAMP4_S30	RNLLEDDSDEEEDFFLR	-1.290	0.042	-0.481	0.350	-0.442	0.333
hsa:10493	VAT1	VAT1_S18	EVAEAATGEDASSPPPKTEAASDPQHPAASEGAAAAAASPPLLR	-2.349	0.039	-1.182	0.204	-1.248	0.139
hsa:80124	VCPIP1	VCPIP1_S1198	GNSVEELEEMDSQDAEMTNTTEPMDHS	0.820	0.817	1.897	0.011	-0.145	0.623
hsa:7416	VDAC1	VDAC1_S104	LTFDSSFSPNTGK	0.016	0.940	0.190	0.197	0.205	0.047
hsa:7417	VDAC2	VDAC2_S115	LTFDTTFSPNTGKK	0.146	0.347	0.233	0.076	0.358	0.002
hsa:7431	VIM	VIM_S29	SYVTTSTR	0.155	0.319	0.219	0.095	0.326	0.009
hsa:7431	VIM	VIM_S10	SVSSSSYR	0.259	0.200	0.182	0.384	0.505	0.031
hsa:7431	VIM	VIM_S73	SSVPGVR	0.221	0.009	0.154	0.266	0.334	0.095
hsa:25962	VIRMA	KIAA1429_S173	HADGEKEDQFNGSPPRPQPR	-1.143	0.035	-1.033	0.059	-1.247	0.093
hsa:55187	VPS13D	VPS13D_S1138	EKDDLSPQPLMTDFER	0.193	0.352	-0.391	0.110	-2.875	0.023
hsa:55187	VPS13D	VPS13D_S1727	SLPSHMEEAPNVFQLYQRPTSASR	-0.045	0.641	0.226	0.396	0.495	0.026
hsa:55187	VPS13D	VPS13D_S1724	SLPSHMEEAPNVFQLYQRPTSASR	0.166	0.449	0.297	0.443	0.506	0.032
hsa:27183	VPS4A	VPS4A_S97	GSDSDSEGDNPEK	2.244	0.052	2.628	0.050	2.979	0.036
hsa:9525	VPS4B	VPS4B_S102	GNDSDGEGESDDPEK	3.534	0.020	3.373	0.052	3.756	0.062
hsa:51322	WAC	WAC_S534_S535	SPSPGPNHTSNSSNASNATVVPQNSSAR	0.509	0.015	0.279	0.200	0.496	0.044
hsa:253725	WASHC2C	FAM21C_S158_S160	AGNSDSEEDDANGR	3.354	0.094	4.163	0.197	4.595	0.043

hsa:253725	WASHC2C	FAM21C_T331_S333	TPSDDEEDNLFAPPK	3.044	0.027	3.352	0.050	2.654	0.099
-	WASHC2D	FAM21D_S1180	SPMFPALGEASSDDDLFQSAKPKPAKK	0.629	0.011	0.405	0.624	0.308	0.768
hsa:57705	WDFY4	WDFY4_S2305	RLSPLEALSSGR	0.256	0.044	-0.294	0.387	-0.047	0.935
hsa:91833	WDR20	WDR20_S357	RNSTDSRPVSVTYR	0.462	0.043	0.566	0.058	0.631	0.086
hsa:55339	WDR33	WDR33_S1219	SSSLQGMDMASLPPR	0.797	0.086	0.981	0.018	0.260	0.409
hsa:10785	WDR4	WDR4_S391	RRSPPPGPDGHAK	-0.466	0.929	-1.591	0.417	-4.638	0.043
hsa:23335	WDR7	WDR7_S935	GSPPTSSNIVQGQIK	0.029	0.923	0.646	0.078	0.802	0.047
hsa:54663	WDR74	WDR74_S382	KRPGSTSP	1.982	0.085	2.788	0.006	3.153	0.004
hsa:54663	WDR74	WDR74_S361	KLSGLEQPQGALQTR	0.243	0.243	0.795	0.031	0.555	0.061
hsa:65125	WNK1	WNK1_S2011	KEKPELSEPSHLNGPSSDPEAAFLSR	-0.842	0.024	-0.671	0.048	-1.028	0.023
hsa:65125	WNK1	WNK1_S2012	KEKPELSEPSHLNGPSSDPEAAFLSR	0.274	0.011	0.117	0.565	0.381	0.714
hsa:56897	WRNIP1	WRNIP1_S65	AKGPSPPGAK	-0.326	0.013	0.137	0.291	0.214	0.096
hsa:9589	WTAP	WTAP_S341	GGSGYVNQLSAGYESVDSPTGSENSLTHQSNDTDSSHDPQEEK	-1.283	0.026	-0.767	0.224	-0.633	0.278
hsa:7508	XPC	XPC_S397_S398_S399	SKPSSSEEDEGPGDKQEK	0.922	0.048	0.655	0.559	-0.155	0.753
hsa:55702	YJU2	CCDC94_S211_S213	LLEDSDSEDEAAPSPLQPALRPNPTAILDEAPKPK	1.608	0.030	1.516	0.051	1.159	0.080
hsa:7528	YY1	YY1_S247	DIDHETVVEEQIIGENSPPDYSEYMTGK	-0.117	0.012	0.234	0.354	0.305	0.246
hsa:7709	ZBTB17	ZBTB17_T119	SLAEPATSPGGNAEALATEGGDKR	-0.485	0.050	-0.228	0.268	-0.709	0.001
hsa:7709	ZBTB17	ZBTB17_S120	SLAEPATSPGGNAEALATEGGDKR	-0.446	0.039	-0.033	0.870	0.022	0.881
hsa:49854	ZBTB21	ZBTB21_S435	IKTEPSSPLSDPSDIIR	1.383	0.225	0.985	0.093	1.112	0.031
hsa:49854	ZBTB21	ZBTB21_S348	SLSMDSQVPVYSPSIDLK	-0.490	0.003	-0.421	0.136	-0.385	0.065
hsa:23091	ZC3H13	ZC3H13_T263_S265	TPSPPPPIPEDIALGKK	2.177	0.055	2.697	0.050	0.921	0.166
hsa:124245	ZC3H18	ZC3H18_S534	KLGVSVSPSR	-0.371	0.232	-0.289	0.162	-0.437	0.025
hsa:124245	ZC3H18	ZC3H18_S487	SPQPPSR	0.846	0.119	0.903	0.079	1.100	0.027
hsa:23211	ZC3H4	ZC3H4_S159	EYSPPYAPSHQQYPPSHATPLPK	-0.640	0.012	-0.613	0.015	-0.711	0.005
hsa:23211	ZC3H4	ZC3H4_Y162	EYSPPYAPSHQQYPPSHATPLPK	-0.643	0.012	-0.620	0.017	-0.699	0.006
hsa:23211	ZC3H4	ZC3H4_S1275	TGSGSPFAGNSPAR	0.141	0.451	-0.099	0.595	0.408	0.046
hsa:23211	ZC3H4	ZC3H4_S1269_S1275	TGSGSPFAGNSPAR	0.938	0.032	0.744	0.127	0.965	0.101
hsa:56829	ZC3HAV1	ZC3HAV1_S284	ASLEDAPVDDLTR	0.804	0.032	0.251	0.290	0.868	0.021
hsa:56829	ZC3HAV1	ZC3HAV1_S257	FFQGSQEFLASASASAER	0.619	0.051	0.587	0.039	0.019	0.922

hsa:55596	ZCCHC8	ZCCHC8_T479	GTPPPVFTPPLPK	0.356	0.153	0.307	0.003	0.331	0.195
hsa:55596	ZCCHC8	ZCCHC8_S598	KSEAGHASSPDSEVTSLCQK	-0.534	0.017	-0.374	0.043	-0.221	0.289
hsa:25921	ZDHHC5	ZDHHC5_S429_T436	SSSLKSAQGTGFELGQLQSIR	-0.422	0.210	-0.157	0.576	-1.069	0.023
hsa:25921	ZDHHC5	ZDHHC5_S296	SKGSLEITESQSADAEPPPPPKPDLSR	-0.638	0.016	0.187	0.280	-0.015	0.973
hsa:9839	ZEB2	ZEB2_S359_S360	TGSSPNSVSSSPTNSAITQLR	-0.430	0.031	-0.130	0.104	-0.089	0.320
hsa:9839	ZEB2	ZEB2_S353	TGSSPNSVSSSPTNSAITQLR	0.378	0.195	0.791	0.034	0.299	0.434
hsa:196441	ZFC3H1	ZFC3H1_S352	RISTSDILSEK	-0.509	0.107	-0.434	0.133	-0.740	0.023
hsa:677	ZFP36L1	ZFP36L1_S54	RHSVTLPSSK	3.811	0.001	5.578	0.000	5.551	0.000
hsa:84936	ZFYVE19	ZFYVE19_S463	EHQTSAYSPPR	-0.280	0.541	0.174	0.178	0.370	0.034
hsa:84936	ZFYVE19	ZFYVE19_S144	WSPPQNYK	0.582	0.081	0.742	0.023	0.396	0.075
hsa:23503	ZFYVE26	ZFYVE26_S615	SPSESPQHIAHPER	-0.227	0.026	0.092	0.626	0.210	0.101
hsa:23613	ZMYND8	ZMYND8_S490_S495	TGQAGSLSGSPKPFSPQLSAPITTK	0.708	0.002	0.886	0.000	0.899	0.010
hsa:23613	ZMYND8	ZMYND8_S425	RISLSDMPR	0.306	0.228	0.627	0.109	0.481	0.034
hsa:64397	ZNF106	ZNF106_S1370	AAHVPENSDTEQDVLTVKPVR	0.170	0.396	0.441	0.025	0.509	0.213
hsa:7739	ZNF185	ZNF185_S465	RESCGSSVLTDFEGK	-0.108	0.475	0.192	0.180	0.228	0.019
hsa:7570	ZNF22	ZNF22_S49	SLDDKPYK	0.150	0.500	0.346	0.380	0.585	0.049
hsa:24149	ZNF318	ZNF318_S2243	SPPREQVIEDNMVPQGMPEQETTVGAIQDHTESSVHN	-0.718	0.012	-0.504	0.061	-0.251	0.034
hsa:163033	ZNF579	ZNF579_S483	AAALQALQAQAPTSPPPPPPPLK	0.885	0.006	0.501	0.036	0.731	0.036
hsa:163033	ZNF579	ZNF579_T482	AAALQALQAQAPTSPPPPPPPLKAEQEEEGLPLPLANIK	-0.734	0.011	-0.109	0.533	0.029	0.941
hsa:22834	ZNF652	ZNF652_S57	ESGSPYSVLVDTK	-0.430	0.047	-0.377	0.377	-0.189	0.449
hsa:57592	ZNF687	ZNF687_S519	NLLPAYRPNLSPPAEAGLALPPTGYR	1.834	0.204	1.895	0.024	1.579	0.059
hsa:283933	ZNF843	ZNF843_T111	EHTLAEALR	0.491	0.025	0.187	0.721	0.175	0.739
hsa:7791	ZYX	ZYX_S259	GPPASSPAPAPK	-0.021	0.776	-0.246	0.016	-0.432	0.025

Table S1: Differentially-phosphorylated peptides from phosphoproteomics analysis of human monocyte-derived macrophages treated with MCTR1, MCTR2, MCTR3, or vehicle.
